# A data-driven network model of primary myelofibrosis: transcriptional and post-transcriptional alterations in CD34+ cells

**DOI:** 10.1038/bcj.2016.47

**Published:** 2016-06-24

**Authors:** E Calura, S Pizzini, A Bisognin, A Coppe, G Sales, E Gaffo, T Fanelli, C Mannarelli, R Zini, R Norfo, V Pennucci, R Manfredini, C Romualdi, P Guglielmelli, A M Vannucchi, S Bortoluzzi

**Affiliations:** 1 Department of Biology, University of Padova, Padova, Italy; 2 Centre for Integrative Biology (CIBIO), University of Trento, Trento, Italy; 3 Department of Molecular Medicine, University of Padova, Padova, Italy; 4 Department of Experimental and Clinical Medicine, University of Florence, Florence, Italy; 5 Department of Life Sciences, University of Modena and Reggio Emilia, Modena, Italy

## Abstract

microRNAs (miRNAs) are relevant in the pathogenesis of primary myelofibrosis (PMF) but our understanding is limited to specific target genes and the overall systemic scenario islacking. By both knowledge-based and *ab initio* approaches for comparative analysis of CD34+ cells of PMF patients and healthy controls, we identified the deregulated pathways involving miRNAs and genes and new transcriptional and post-transcriptional regulatory circuits in PMF cells. These converge in a unique and integrated cellular process, in which the role of specific miRNAs is to wire, co-regulate and allow a fine crosstalk between the involved processes. The PMF pathway includes Akt signaling, linked to Rho GTPases, CDC42, PLD2, PTEN crosstalk with the hypoxia response and Calcium-linked cellular processes connected to cyclic AMP signaling. Nested on the depicted transcriptional scenario, predicted circuits are reported, opening new hypotheses. Links between miRNAs (miR-106a-5p, miR-20b-5p, miR-20a-5p, miR-17-5p, miR-19b-3p and let-7d-5p) and key transcription factors (MYCN, ATF, CEBPA, REL, IRF and FOXJ2) and their common target genes tantalizingly suggest new path to approach the disease. The study provides a global overview of transcriptional and post-transcriptional deregulations in PMF, and, unifying consolidated and predicted data, could be helpful to identify new combinatorial therapeutic strategy. Interactive PMF network model: http://compgen.bio.unipd.it/pmf-net/.

## Introduction

Primary myelofibrosis (PMF) is a chronic myeloproliferative neoplasm (MPN) that, with essential thrombocythemia and polycythemia vera, constitutes a heterogeneous group of Philadelphia-negative clonal hematopoietic stem cell (HSC) disorders associated with overproduction of mature myeloid cells. Typical traits of myelofibrosis are an increased proliferation of megakaryocytes, a deposition of fibrosis in the bone marrow, an abnormal stem cell trafficking and an extramedullary hematopoiesis (myeloid metaplasia). Moreover, PMF is associated with an increased risk of thrombosis and/or hemorrhage and a propensity to develop acute myeloid leukemia.^1, 2^

Important progresses in molecular characterization of MPNs pathogenesis have been done in the last years. Specifically, the discoveries of somatic mutations in JAK2, MPL and CALR genes have improved patients' stratification and molecular characterization highlighting the role of Jak-STAT signaling in MPN pathogenesis. However, several evidences indicate that these mutations are not sufficient for disease initiation and progression and that the phenotypes of the disease are highly heterogeneous, suggesting that other unknown genetic or epigenetic factors might be involved^3, 4, 5, 6, 7, 8, 9, 10, 11, 12, 13, 14, 15^ and also that the mutation order could matter:^16^ driver mutations can precede or follow additional somatic mutations in other myeloid genes. Interestingly, recently, it has been demonstrated that most of these genes are associated with age-related clonal hematopoiesis in normal elderly subjects, suggesting that pre-malignant clones may be present many years before disease develops and are required, but insufficient, to result in disease.^17, 18^

microRNAs (miRNAs) have an important role in the regulation of hematopoiesis.^19, 20, 21, 22^ Our group demonstrated that miR-16-2 contributes to the expansion of erythroid lineage in polycythemia vera^23^ and we showed that miR-155-5p is pathogenically associated to MK hyperplasia in PMF through JARID2 downregulation.^24^ Moreover, we recently characterized miRNA and microRNA offset RNAs (moRNA) expression in SET2 cells^25^ and in CD34+ stem cells using massive small RNA-seq. In the latter study, we observed specificities in small RNAs expression of PMF cells.^26^ Although these findings are supportive of the involvement of miRNAs in PMF pathobiology, our understanding of miRNAs involvement in MPNs is still limited.^27^ A deeper characterization of the miRNA-mediated pathogenesis processes^19, 28^ would be highly desirable in order to identify suitable concurrently targetable pathways amenable to therapeutic intervention.^28, 29^

Thereby, the aim of this study was to obtain an informative, composite and interactive data-driven picture of pathways and circuits deregulated in PMF. We used a composite pipeline exploiting both ‘knowledge-based' and *ab initio* approaches to discover mixed transcriptional and post-transcriptional deregulated circuits in PMF. This strategy allowed us to describe an unforeseen picture of miRNA and gene regulatory circuits linked to abnormal cellular functions and pathways.

## Methods

### Patient selection and expression data

For this study, we considered miRNA and gene expression data (series GSE41812 and GSE53482) obtained by analyzing CD34+ cells of 42 patients with a diagnosis of PMF and 16 peripheral blood (CTR PB) and 15 bone marrow (CTR BM) samples from normal donors. Primary myelofibrosis patients were in a typical fibrotic stage of the disease according to the WHO, and were molecularly characterized (*JAK2V617F, MPLW51* and *CALR* mutations). See Norfo *et al.*^24^ for additional clinical information about patients.

Gene expression profiling, obtained by Affymetrix HGU219, and annotated by using custom CDF,^30^ HGU219_Hs_ENTREZG version 14, was normalized by RMA procedure. A log_2_ expression matrix with 18 654 genes and 73 samples was obtained.

MiRNA expression profiling, obtained by Affymetrix miRNA 2.0 microarray, was analyzed by using a modified RMA procedure: the background subtraction was performed considering all species and controls of the chip, while normalization and summarization were calculated only on human probe sets, using a custom-designed CDF. MiRNA names were translated to updated miRBase names filtering out deprecated miRNAs, using the custom annotation package AffyIDs. A log_2_ expression matrix of 584 miRNAs and 73 samples was obtained after filtering miRNAs weakly expressed (lowest 25% of average expression level) and/or poorly variable across samples (Shannon Entropy<1.4).

### Identification of negatively correlated predicted targets of miRNAs

We identified the predicted regulatory relations supported by our expression data by integrating target predictions and expression profiles. Target predictions were computed with TargetScan-62 algorithm according sequence similarity with conservation (threshold at 0.8).

Pairwise Pearson correlations between miRNA and predicted target gene expression profiles were calculated. The relationships supported by significant anti-correlation (*r*<−0.5 with FDR<0.1) were considered for the following analyses.

The miRNA/target interactions involving genes annotated in KEGG pathways have been used in the following *Micrographite* pipeline.

### Pathways-derived circuits

Two comparisons were considered for the analysis: PMF vs CTR BM and PMF vs CTR PB. mRNA and miRNA relations involved in PMF were compared to CTR PB or CTR BM and have been identified using the *Micrographite* pathway analysis.^31^ In the following we will report a brief description of the main steps of the method; for details refers to Calura *et al.*^31^

KEGG^32^ pathways as stored in graphite Bioconductor package^33^ were enriched with validated and predicted miRNA-target interactions. We consider ‘validated interactions' those interactions extracted from miRTarBase^34^ and miRecords^35^ for which reporter assays have been provided in literature, while the ‘predicted/supported interactions' are the *in silico* predicted miRNA-mRNA couples filtered by anticorrelation of expressions as discussed above. In this step, only miRNAs targeting genes already annotated in the cell pathways have been added. Following the *Micrographite* pipeline, the pathways resulted to be significant at the whole pathway level (q-value for the mean and variance tests<=0.1) were used for the second step of the analysis that identifies, within each significant pathway, the subportion of the pathway (hereafter called path) mostly associated to the phenotype. The merge of the top 10 paths is called meta-pathway. The meta-pathway is then re-analyzed in order to find the top scored paths. Finally, the paths that, ranked by scores, belong to upper quartile are considered as the most involved and reported. The *Micrographite* analysis has been performed independently for each comparison (PMF vs CTR PB; PMF vs CTR BM) and the two final networks have been merged into a single network.

### Mixed miRNA-TF-gene circuit identification

In parallel, the expression data of genes that are present in the KEGG pathways were used with the miRNAs profile as input of Magia^2^. Magia^2^ (http://gencomp.bio.unipd.it/magia2)^36^ reconstructs circuits involving positive and negative correlations between miRNAs, transcription factors (TFs) and target genes. It uses experimentally validated TF–miRNA relations obtained by mirGen2.0 and TransmiR, and TF-gene relations obtained by the UCSC human genome annotation.

Target predictions were computed with TargetScan (score threshold at 0.8, top 25% of the predictions distribution) and Pearson's correlation coefficient was used as correlations measure (threshold at 0.5).

### Pathways visualization

Network visualization and annotation have been performed using Cytoscape^37^ (version 3.1).

### Validation of differentially expressed sRNAs

We performed individual miRNAs assay by Taqman quantitative real-time PCR for quantification of abnormally expressed miRNAs in PMF and control granulocytes and plasma. All samples were processed within 4 h after collection. Plasma, collected by peripheral blood centrifugation in tubes containing EDTA (4 °C, 3000 revolutions per minute for 15 min, then 6000 revolutions per minute for 5 min), was stored at -80 °C in RNase/DNase-free Eppendorf tubes. RNA was isolated and eluted in 20 μl of RNase-free water using a miRNeasy Serum/Plasma Kit (Qiagen). For miRNA expression assays 8 μl eluted RNA was used.^38^ cDNA was synthesized from total RNA using miRNA-specific RT primers contained in the TaqMan microRNA Human Assays (Applied Biosystems, Foster City, CA, USA). Briefly, single-stranded cDNA was synthesized from 10 ng total RNA in 15-μl reaction volume with the High-Capacity cDNA Archive Kit (Applied Biosystems) using 1 mM deoxyribonucleoside triphosphates, 50 U Multiscribe reverse transcriptase, 3.8 U RNase Inhibitor and 50 nM of miR-specific RT primers. The reaction was incubated at 16 °C for 30 min followed by 30 min at 42 °C, and inactivation at 85 °C for 5 min. Each generated cDNA was amplified by quantitative real-time-PCR with sequence-specific primers from the TaqMan microRNA Assays on an ABI Prism 7300 real-time PCR system (Applied Biosystems). PCR reactions included 10 μl 2 × Universal PCR Master Mix (No AmpErase UNG), 2 μl each 10 × TaqMan MicroRNA Assay Mix and 1.5 μl reverse-transcribed product; they were incubated in a 96-well plate at 95 °C for 10 min, followed by 40 cycles of 95 °C for 15 s and 60 °C for 1 min. Expression variations were calculated using the RQ method and a *t*-test *P*-value of 0.05 was used as threshold to identify differentially expressed elements.

## Results and discussion

### A systems biology pipeline identifies deregulated signaling pathways in PMF patients

**Figure 1:**
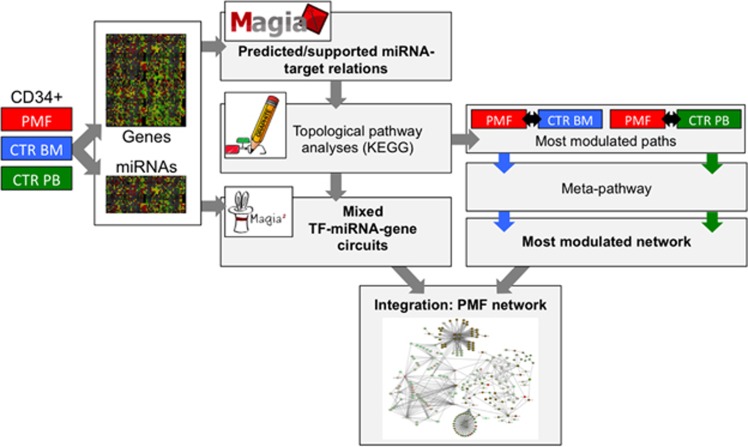
Experimental design and analysis procedures flow chart. miRNA and gene expression data in CD34+ cells of PMF patients and healthy controls were considered; CTR BM and CTR PB controls were considered separately in parallel analyses, and results were merged at the final steps. MAGIA integrated analysis outputted the subset of predicted miRNA-target relations supported by expression data; these were used to enrich KEGG pathway-derived miRNA-gene networks based on pathways annotations and on validated miRNA-target relations. Topological pathway analyses by *Micrographite* identified most modulated paths, showing significant gene/miRNA expression variations and changes in relations strength, then a non-redundant comprehensive meta-pathway was derived; iterative analysis of the meta-pathway identified the most modulated network for each comparison. Magia^2^ analysis identified mixed TF-miRNA-gene circuits. Results of parallel comparisons were merged in an integrated network.

We considered matched expression profiling data of 584 miRNAs and 18 654 genes in 73 CD34+ cells sampled from 42 PMF patients, and 31 sampled from normal subjects (15 from bone marrow (CTR BM) and 16 from peripheral blood (CTR PB). The study grounds on comparative analysis of CD34+ cells of PMF patients compared with control CD34+ from normal donors. In a previous study, specificities of both gene and miRNA expression profiles were observed comparing peripheral blood and bone marrow control samples,^24^ confirming known differences between these populations. Thus, peripheral blood and bone marrow control samples were considered separately, for parallel comparisons with PMF samples, to avoid biases due to control samples inhomogeneity, and to obtain more informative results. Then, a union of the comparisons gave a global picture. The flowchart in Figure 1 provides a summary of the procedure.

**Figure 2:**
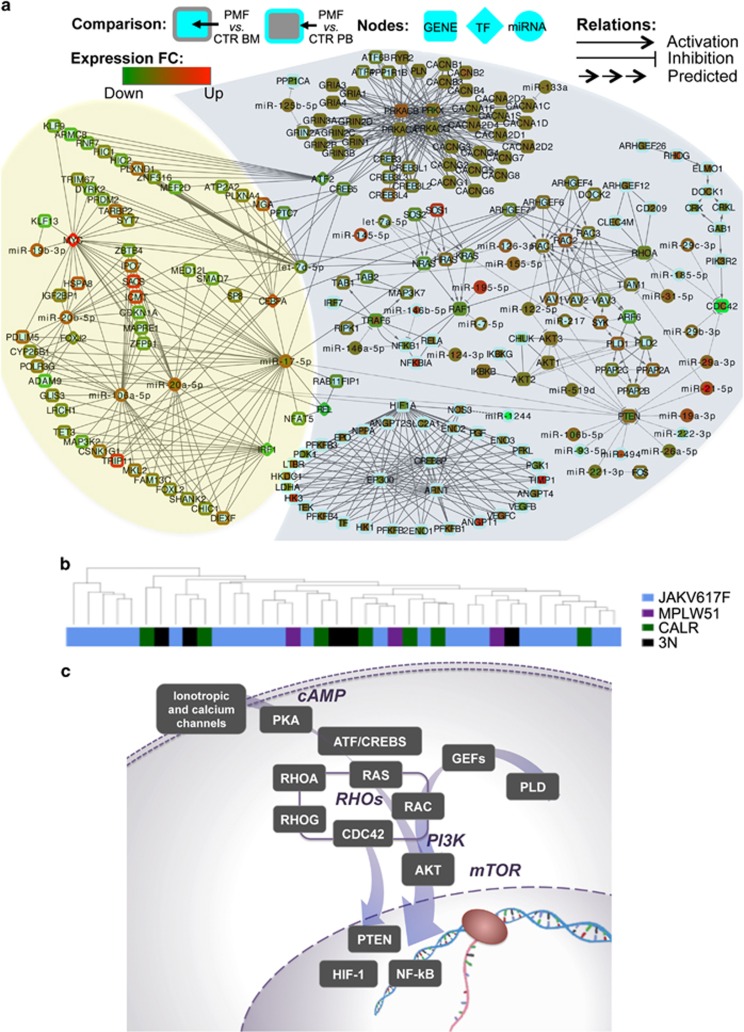
PMF network model. (**a**) PMF network integrating pathway-derived miRNA-gene networks deregulated in PMF and mixed TF-miRNA-gene circuits discovered by reverse engineering of expression data. The network (see Supplementary Figure 3 for a larger version) gives a non-redundant and comprehensive picture of most modulated paths in the two PMF vs CTR comparisons, of the impact of miRNAs on pathway genes, and of connected TF-miRNA-gene mixed circuits discovered in the study. Genes are reported as round rectangles, transcriptional factors as diamonds and miRNAs as triangles. Node colors represent the fold-change (FC) of the gene expressions in the PMF vs CTR BM (node inner color) and PMF vs CTR PB (node border color). The type of edges depends on the type of interaction: arrow for activation, T arrow in case of inhibition, and arrow line for miRNA-target predicted/supported interactions. The light blue shade indicates the part of the network resulting from miRNA and gene topological pathway analysis. The yellow shade indicates mixed TF-miRNA-gene circuits, inferred by Magia^2^ analysis, connected to the path-derived network. (**b**) Cluster analysis of expression profiles of miRNAs and genes included in the final PMF network do not show clustering of PMF patients by mutation. Samples are colored according to the carried mutation as shown in the legend (3N indicates triple negative). Sample clustering was obtained according to Euclidean distance and complete clustering. See Supplementary Figure 4 for the corresponding heatmap. (**c**) Summary of most deregulated pathways represented in the miRNA-gene network, and connections thereof. See http://compgen.bio.unipd.it/pmf-net/ for an interactive, searchable and zoomable version of the PMF network model.

We obtained 250 interactions in the PMF vs CTR BM (54 miRNAs and 236 gene targets involved) and 442 in the PMF vs CTR PB (43 miRNAs and 428 genes) using MAGIA^2^. Then, the miRNA and gene expression profiles of PMF patients have been independently compared with CTR BM and CTR PB health samples using *Micrographite*. Supplementary Tables 1-8 contain all the intermediate data of the two analyses whereas a brief comparison of results is summarized in Supplementary Figures 1 and 2. The resulting networks of the two analyses were merged achieving a set of 181 non-redundant genes and miRNAs and their interactions that represent the compendium of the deregulated pathway-guided interactions in PMF. The network contains a selection of 401 gene-gene consolidated interactions from KEGG pathways, 45 miRNA-gene connections already validated with gene reporter assays and only one in silico predicted anti-correlated miRNA-gene connection (Figure 2a). See http://compgen.bio.unipd.it/pmf-net/ for an interactive, searchable and zoomable version of the PMF network model.

### Mixed miRNA-TF circuits connect to altered pathways

miRNAs post-transcriptionally regulate gene expression, also by alteration of target mRNAs stability. Moreover, TFs modulate the expression of miRNAs and of target genes at transcriptional level, creating a regulatory network. Magia^2^ analysis identified mixed regulatory circuits composed of miRNAs, TFs and target genes, considering in parallel expression profiles in PMF and CTR BM samples and in PMF and CTR PB samples. For each type of control samples used, Supplementary Tables 9–12 report more details on observed correlation values, on the numbers of identified circuits, on predicted interactions and on involved genes and miRNAs. Inferred interplay of miRNAs and TFs in gene/transcripts expression regulation may comprise two types of mixed regulatory circuits: (i) a TF regulates both a miRNA and a gene promoter, and the same miRNA targets the gene mRNA exerting post-transcriptional regulation; (ii) a miRNA post-transcriptionally regulates both a gene encoding a TF and another gene, that on turn is a transcriptional target of the TF. For both mixed circuits topologies, different combinations of positive and negative pairwise correlations should correspond to activatory and inhibitory interactions giving rise to coherent or incoherent feed forward loops.^36, 39^ Since circuits based on strongest interactions are particularly trustable and may highlight key regulatory relations, we focused on circuits that had absolute correlation at least 0.5 for all the three types of interactions (miRNA-target gene, TF-miRNA, TF-target-gene). PMF vs CTR PB comparison identified 169 interactions, involving 63 nodes, 6 miRNAs and 57 genes. No significant circuits were selected for PMF vs CTR BM comparison.

Pathway annotations are progressively manually updated but, even if enriched with miRNA validated interactions, they are still incomplete. Thus, we enriched the pathway-based networks with nodes and relations coming from selected Magia^2^ circuits, that shared with the first network at least one gene or one miRNA. In this way, we obtained the network, consisting of 620 edges and 220 nodes (of which 34 are miRNAs; Table 1); they are reported, with different details and annotation, in Supplementary Figure 3 and are included in the yellow area of the circuit in Figure 2a.

Table 1 summarizes miRNAs and TFs involvement. Magia^2^ circuits showed that six miRNAs and six TFs seem to play critical roles in the regulation of transcription in PMF (Figure 2a and Table 1). Almost all of the miRNAs are components of miR-17 family (miR-106a-5p, miR-20b-5p, miR-20a-5p, miR-17-5p and miR-19b-3p). The upregulation of the latter miRNA in PMF CD34+ cells has been previously validated.^24^ Only let-7d-5p is a component of another family of miRNAs, the let-7 family, which is present also in *Micrographite* results. The TFs (MYCN, ATF, CEBPA, REL, IRF and FOXJ2) are important regulators of several target-genes. Relevant contribution in PMF vs CTR PB comparison is done by MYCN gene, in particular the upregulation of the miR-17 family components and the downregulation of let-7d-5p. The bridge elements between the pathway network and predicted circuits are two miRNAs (miR-17-5p and miR-20a-5p) and four genes (ATF, CREB5, NRAS and ARHGEF7).

Different genetic lesions characterizing PMF patients (28 JAKV617F, 7 CALR, 4 MPLW51 and five triple negative, 3N, for the former mutations) included in this study are representative of the molecular alterations described in PMF. Given such patient heterogeneity, we checked if some of the genes/miRNAs modulated in PMF were deregulated only, or mainly, in a subgroup of patients. As shown in Figure 2b, and in Supplementary Figure 4, cluster analysis of samples based on expression profiles of miRNAs and genes included in the PMF network of Figure 2a did not highlight any correlation with the presence of a specific mutation. Therefore, results of cluster analysis suggest that our results provide a comprehensive picture of deregulated elements and relations thereof, that are common to all PMF patients, independent of the underlying driver mutation. Accordingly, fold-changes against control cells have been recalculated stratifying patients by driver mutations; these data do not show mutation-specific contributions in the here presented network (Supplementary Figure 5).

### Confirmation of miRNA and gene differential expression in PMF

Genes and miRNAs are included in the PMF network only if they have shown coordinated expressions across patients and opposite behaviors if compared to healthy donors. Specifically, modulated genes and miRNA are included in the PMF network if they are either linked according to validated relations (pathway-derived part of the network and TF-gene or TF-miRNA relations in the part containing MAGIA2 circuits) or linked by predicted miRNA-target relations that are anyway significantly supported by expression data analysis.

Even if the multistep approach we used for the PMF network reconstruction was specifically designed to go beyond the differential expression of single genes or miRNAs, giving a more informative picture of the impact of miRNAs deregulated in PMF to pathways and to mixed connected regulatory circuits it is worth noting that we obtained confirmation of the differential expression in PMF samples compared to controls for 13 miRNA and 5 genes included in the network, giving further support to our results. As detailed in Table 2, in addition to the confirmation of differential expression in CD34+ previously obtained by array and quantitative real-time-PCR^24^ and RNA-seq,^26^ we also conducted new experiments in granulocytes and plasma (see Methods) to obtain new insights of direct translational relevance. These confirmed a significant upregulation (*P*-value<0.05) of miR-195-5p, miR-106a-5p, miR-145-5p, miR-17-5p, miR-185-5p, miR-195-5p in PMF patients granulocytes (GR; considering 50 PMF patients and 10 healthy controls), miR-19b-3p both in PMF granulocytes and plasma (25 PMF patients and 6 controls) and of miR-29b-3p in plasma.

### Candidate transcriptional deregulated pathways of PMF

**Figure 3:**
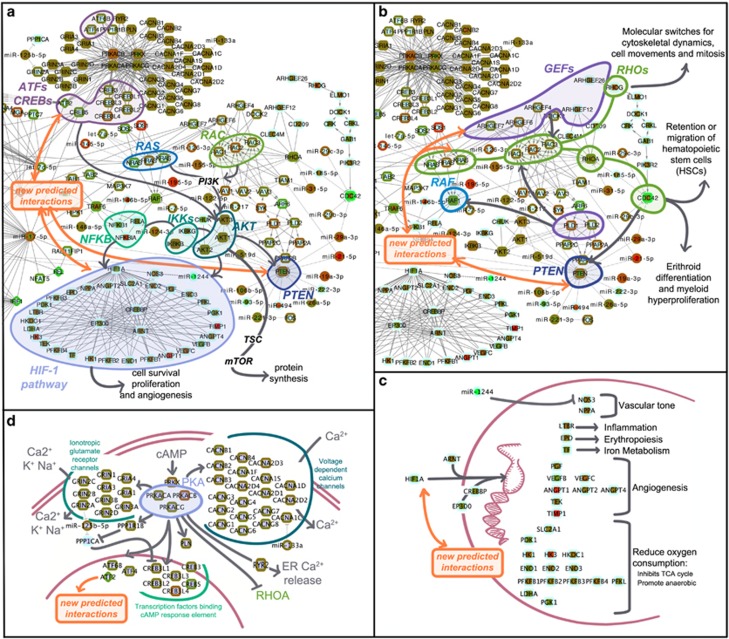
Details of the PMF network model showing deregulated miRNAs and genes that participates to specific connected pathways, linked in turn to biological processes and functions germane to the disease. (**a**) Akt signaling; (**b**) Rho GTPases, CDC42, PLD2 and PTEN; (**c**) HIF-1a pathway; (**d**) Calcium signaling.

Figure 2c summarizes the main alterations of the transcript levels observed in PMF patients, involving key pathways and connected circuits, as discussed below in more detail with the support of Figure 3.

#### Akt signaling

One of most deregulated pathways is the Akt signaling, a part of the Phosphoinositide-3 kinase/protein kinase-B/mammalian target of Rapamycin (PI3K/Akt/mTOR) pathway (Figure 3a). As the Jak/STAT signaling path, the Akt signaling is triggered by receptor tyrosine kinases that increase PI3K levels, activating AKT, phosphorylating several targets including mTOR. Several tumor suppressor genes, as PTEN, interact with this path. PI3K/AKT/mTOR is a central signaling module and several other pathways converge on it regulating cell survival and proliferation.^28^ It is constitutively activated in MPNs and also in other cancers.^28^ Small molecular inhibitors of this pathway are available in clinics,^23^ and evidence of activity of an mTOR inhibitor (Everolimus) in myelofibrosis patients has been provided.^40^ The contribution of PI3K/mTOR pathway to MPN pathogenesis has received strong experimental support.^28, 41, 42^

#### Rho GTPases, CDC42, PLD2 and PTEN

The PMF network shows deregulation of Rho GTPases (Figure 3b), intracellular signal transducers, members of the RAC subfamily of Rho GTPases. Deregulation of GEFs (guanine nucleotide exchange factors), recharging Rho GTPases and determining their spatial-temporal activity is also displayed. Interaction of stem cell factors and adhesion molecules with their receptors (c-Kit, CXCR4 and Integrins) expressed by the HSCs triggers the activation of GEFs.^43^ Rho GTPases could affect also cancer progression,^44^ both as oncogene and oncosuppressor. Specifically, CDC42 and RAC2 control homeostasis of blood cell production by hematopoietic stem/progenitor (HSC/P) cells balancing HSC/P retention within the bone marrow and migration in the blood.^45^

CDC42, highly downregulated in PMF,^20^ regulates cell polarity and cell cycle progression. Its deletion affects the number of early myeloid progenitors while suppressing erythroid differentiation.^46^ CDC2-deficient mice develop a fatal myeloproliferative disorder manifested by significant leukocytosis with neutrophilia, myeloid hyperproliferation and myeloid cell infiltration into distal organs.^46^ Downregulation of RhoA activity, as observed in PMF, resulted in increased HSC engraftment and self-renewal. In the network, CDC42 is connected with the upregulated miR-29a-3p, which we previously demonstrated is able to suppress their expression^24^ and which is connected also to the phosphatase PTEN, regulated by RhoGTPases.^47^ In turn, PTEN, down-regulated in PMF (and linked to several miRNAs including the upregulated miR-19a-3p, miR-29a-3p and miR-21-5p), modulates intracellular levels of PI3K in cells, functioning as a tumor suppressor. The upregulation in PMF of miRNAs interacting with PTEN was previously established.^24^ CDC42 is also connected with miR-29b-3p, miR-29c-3p and miR-185-5p, the latter linked also with the downregulated RHOA.

Another element markedly deregulated in the PMF network is Phospholipases D, PLD1 and PLD2 (Figure 3b). Phospholipase D and its product PA (Phosphatidic Acid) have been implicated in many physiological and pathological functions, such as cancer cell invasion and metastasis.^48, 49, 50, 51, 52^ PLD2 is dual activity enzyme with both lipase and GEF activities for the Rac2 and RhoA GTPases. As demonstrated by Mahankali *et al*,^53^ in the context of leukocyte chemotaxis, both activities are triggered after cell stimulation, but then, the accumulation of RAC2-GTP, due to GEF action, negatively feeds back on PLD2-lipase activity, and the JAK3 tyrosine kinase restores lipase activity while ultimately inhibiting GEF activity.^54^ This lipase-GEF duality has important biochemical and cellular implications and new PLD inhibitors have been recently described.^52, 54^ PA is one of the major lipid second messengers that in turn triggers many signaling pathways such as PI3K/Akt/mTOR, sphingosine kinase 1, Raf-1 protein kinase^53^ and small GTPases like Ras and Rac.^55^

#### HIF-1a pathway

In connection with previously mentioned pathways, we found evidence of modulation of the HIF-1a hypoxia pathway (Figure 3c). As known, both growth factors and oncogenic signaling can trigger an HIF-1a response, with the PI3K/Akt and Ras/Raf/MAPK pathways mediating upregulation of HIF-1a by increasing HIF-1a protein synthesis or stability, resulting in a modified transcriptional activity of HIF-1. In our network, the predicted/supported relation of the still poorly characterized miR-1244 (downregulated in PMF) with NOS3 (nitric oxide synthase 3, endothelial cell) is connected with HIF-1a while the upregulated miR-20a-5p connects HIF-1a to PTEN. NOS3 synthesizes nitric oxide, that is involved in several processes such as muscle relaxation, inhibition of platelet aggregation and leukocyte adhesion, with both vasodilatatory and antithrombotic properties. A recent study showed that NOS3 Glu298Asp polymorphism in homozygosis increased the risk of thrombosis in a cohort of patients with essential thrombocythemia or prefibrotic PMF.^56, 57^

A link between hypoxia and the activation of the HGF/Met axis, through the oxygen sensor HIF-1a in MPNs, has recently been proposed by Boissinot and colleagues.^58^ Oxygen tension is as low as 1% in normal bone marrow^59^ and the hypoxic condition may result even more stringent because of the bone marrow hypercellularity in myelofibrosis. In accordance with our results, showing activation of the HIF-1a downstream genes (Figure 3c) and of NF-κB signaling (Figure 3a), the authors proposed that the activation of the HGF/Met axis, due to bone marrow or/and exposure to inflammation cytokines (NF-κB-mediated activation), leads to enhanced survival and proliferation of mutated myeloid progenitors and increased production of various cytokines responsible for inflammation, neo-angiogenesis and fibrosis. Thus, molecules efficient at blocking NF-κB and HIF-mediated activation might represent an efficient therapy for MPNs by simultaneously blocking the cascade of inflammation cytokines, their signaling and the hematopoietic clone.

#### Calcium and cyclic AMP signaling

The upper part of the network in Figure 2a includes genes, belonging to Calcium signaling path (Figure 3d). More specifically, this part of the network includes genes encoding several subunits of calcium channel voltage-dependent, and other genes, including ATF/CREB subunits activated by cyclic AMP-dependent protein kinases, tightly related to MAPK and Ras signaling pathways, Chemokine signaling and Apoptosis. ATF/CREB genes participate also to PI3K/Akt signaling pathway and to TNF signaling pathway. The Calcium, and c-AMP networks include, as possible modulators, miR-125b-5p (that might target the downregulated PPP1CA and GRIN2A) and miR-133a (that might target CACNA1C).

CALR mutations are found in PMF and essential thrombocythemia patients. Calreticulin is a multifunctional protein that acts as a major Ca^2+^-binding (storage) protein and chaperone in the lumen of the endoplasmic reticulum and intervene in transcription regulation.^60^ CALR has been implicated in the development of different cancers and its effect on tumor formation and progression may depend on cell types and clinical stages.^60^ CALR is not differentially expressed in PMF compared to controls and not present in the network (PMF equal to CTR PB controls and only slightly upregulated in PMF in comparison to CTR BM controls) evidencing that genetic alterations of PMF patients do not affect significantly the transcriptional behavior of the CALR gene. However, many CALR interactors and genes participating in CALR-involving pathways are represented in the PMF network: ATF4 and ATF6 participate to unfolded proteins response (Protein processing in endoplasmic reticulum), and transduce to the nucleus ER stress, eventually inducing apoptosis; CD209 and RAC1 participate to the Phagosome; several genes, including FOS/JUN and NFKB are in the Chagas disease path; ATF2/CREB, NFAT, EP300 and RAS genes share with CALR the HTLV-1 infection path.

The Ca^2+^-related part of the net is connected to the TFs ATF2 and CREB. Several circuits show cross-talk of ATF2 and let-7d-5p, both downregulated in PMF, to 12 correlated genes, mostly downregulated in PMF (RNF7, HIC1, HIC2, PRKACG, PRKX, PRKACA, PRKACB, MEF2D, ARMC8, KLF9, ZNF516). Among them *HIC1/2* are tumor suppressor genes,^61^ known to be hypermethylated in cancer and in high-risk myelodisplastic syndromes;^62^ ZNF516 is a zinc-finger protein that interacts with epigenetic regulation protein such as HDAC1 and KDM1A. The upregulated PLXND1 is one of Semaforin receptors, often highly expressed in cancer,^63^ that regulates tumor cell survival by suppressing an apoptotic pathway^64^ and seems to controls angiogenesis in cancer.^65^ let-7d-5p has also correlated expression with MYC with which its shares KLF13 as putative target. CREB connects to miR-20a 5p and to MYC, and in turn to their common targets.

### Predicted circuits open new hypotheses

All the deregulated pathways contain links to novel *in silico* predicted interactions that could help in the candidate selection in research. In support of the results consistency, a subset of *de novo* predicted interactions is known and validated, that is, the upregulated miR-17-5p, miR-106a-5p and miR-20b-5p with the downregulated CDKN1A. CDKN1A is an important intermediate by which p53 blocks proliferation in response to DNA damage, and it inhibits CDK activity, blocking cell cycle progression. The validated post-transcriptional regulatory relations are also linked to MYC (highly upregulated in PMF) transcriptional activity, which is known to stimulate the expression of onco-miR-17 family. These, in turn suppress IRF1, a tumor suppressor TF that is downregulated in PMF and has important supported targets, as MAP3K2, GLIS3 and LRCH1, all downregulated.

Four upregulated miRNAs (miR-17-5p, miR-106a-5p miR-20a-5p and miR-20b-5p) are negatively correlated with the downregulated TF FOXJ2 (Forkhead box protein J2), forming triangular circuits with four genes including the upregulated PDZ and LIM domain 5 (PDLIM5), recently identified as the main signaling molecule that, in the context of augmented AMPK activity and in connection with Rac1-mediated acting cytoskeleton reorganization,^66^ regulates cell migration, a key process in MPNs.

## Conclusions

The network inferred in this work is a unique and integrated signaling pathway of PMF, in which the role of miRNA is to wire, co-regulate and allow a fine crosstalk between all the involved processes. Most deregulated pathways include Akt signaling, linked to Rho GTPases, CDC42, PLD2 and PTEN crosstalk with the hypoxia pathway, which highlights the extreme hypoxic status in PMF bone marrow. Novel hints on cyclic AMP signaling involvement jointly connected with Calcium-linked cellular processes were given. Moreover, mixed circuits involving miRNAs (miR-106a-5p, miR-20b-5p, miR-20a-5p, miR-17-5p, miR-19b-3p and let-7d-5p) connections with key TFs (MYCN, ATF, CEBPA, REL, IRF and FOXJ2) and their common target genes were reported, opening new hypotheses. The comprehensive picture of transcriptional deregulation can be used to improve current understanding of the molecular pathways involved in the disease and delineate new combinatorial therapeutic strategies.

## Figures and Tables

**Table 1:** miRNAs impacting in pathway-derived miRNA-gene networks deregulated in PMF and participating to mixed TF-miRNA-gene circuits

*Main pathways and genes*	*miRNAs*
Ca(2+) signaling	miR-125b-5p
	miR-133a
CDC42	miR-185-5p
	miR-29b-3p
	miR-29c-3p
HIF-1 alpha hypoxia pathway	miR-1244
NF-kappaB signaling	miR-124-3p
	miR-146a-5p
	miR-146b-5p
PTEN signaling	miR-106b-5p
	miR-19a-3p
	miR-20a-5p
	miR-21-5p
	miR-221-3p
	miR-222-3p
	miR-26a-5p
	miR-494
	miR-519d
	miR-93-5p
PTEN-GEF crosstalk	miR-17-5p
PTEN-CDC42 crosstalk	miR-29a-3p
RAS-PTEN crosstalk	miR-217
RAS/RAS signaling	miR-195-5p
	miR-7-5p
	let-7a-5p
	miR-122-5p
	miR-126-3p
	miR-145-5p
RAS/RAF signaling, GEF activity	let-7d-5p
RHOA	miR-31-5p
RHOA, MAPK and NF-kappaB signaling	miR-155-5p
	
*Transcription factors*	*miRNAs*
MYC	miR-19b-3p
ATF2, CEBPA, MYC	let-7d-5p
FOXJ2, MYC, IRF1	miR-106a-5p
FOXJ2, MYC	miR-20b-5p
REL, CEBPA, FOXJ2, MYC, IRF1	miR-17-5p

Abbreviations: miRNA, microRNA; PMF, primary myelofibrosis; TF, transcription factor.

The upper part of the table indicates the pathways or genes targeted by miRNAs in PMF; the lower part of the table indicates the transcription factors for which the interplay with miRNAs has been *in silico* inferred.

**Table 2:** miRNAs and genes included in the network for which differential expression in PMF patients compared to healthy controls has been confirmed

*PMF* vs *CTR*	*CD34+*			*GR and Plasma*
	*Arrays*	*RNA-seq*	*RT-PCR*	*RT-PCR (PMF* vs *CTR; RQ mean±dev)*
miR-106a-5p	UP			UP GR (1.9±1.2 vs 1.08±1.0)
miR-145-5p	UP			UP GR (1.7±0.9 vs 1.0±0.5)
miR-146b-5p	UP		UP	
miR-155-5p	UP		UP	
miR-17-5p	UP			UP GR (1.5±0.7 vs 1.1±0.9)
miR-185-5p	UP			UP GR (3.0±3.1 vs 1.5±1.2)
miR-195-5p	UP		UP	UP GR (15.3±10.5 vs 1.8±2.1) UP Plasma (12.4±17.5 vs 1.37±0.95)
miR-19a-3p	UP		UP	UP GR (15.3±10.5 vs 1.8±2.1) UP Plasma (12.4±17.5 vs 1.37±0.95)
miR-19b-3p	UP	UP	UP	
miR-21-5p	UP		UP	
miR-29a-3p	UP	UP	UP	UP Plasma (3.0±3.1 vs 1.5±1.2)
miR-29c-3p	UP		UP	
miR-494-3p	UP	UP	UP	
ANGPT1	UP		UP	
ARHGEF7	DOWN		DOWN	
CDC42	DOWN		DOWN	
MEF2D	DOWN		DOWN	
SMAD7	DOWN		DOWN	

Abbreviations: miRNA, microRNA; PMF, primary myelofibrosis.

The second column indicates if miRNAs and genes resulted up- or down-regulated according to array data used for network reconstruction. The following columns indicate for which elements a significant differential expression has been previously confirmed using RNA-seq in CD34+ cells,^22^ and using RT-PCR in CD34+ cells,^20^ in granulocytes (GR) and in plasma samples.

## Notes

The funders had no role in study design, data collection and analysis, decision to publish, or preparation of the manuscript. The authors declare no conflict of interest.

## References

[bib1] Tefferi A, Vardiman JW. Classification and diagnosis of myeloproliferative neoplasms: the 2008 World Health Organization criteria and point-of-care diagnostic algorithms. Leukemia 2007; 22: 14–22. 17882280 10.1038/sj.leu.2404955

[bib2] Vannucchi AM, Guglielmelli P, Tefferi A. Advances in understanding and management of myeloproliferative neoplasms. CA Cancer J Clin 2009; 59: 171–191. 19369682 10.3322/caac.20009

[bib3] Baxter EJ, Scott LM, Campbell PJ, East C, Fourouclas N, Swanton S et al. Acquired mutation of the tyrosine kinase JAK2 in human myeloproliferative disorders. Lancet Lond Engl 2005; 365: 1054–1061. 10.1016/S0140-6736(05)71142-9 15781101

[bib4] Jones AV, Kreil S, Zoi K, Waghorn K, Curtis C, Zhang L et al. Widespread occurrence of the JAK2 V617F mutation in chronic myeloproliferative disorders. Blood 2005; 106: 2162–2168. 15920007 10.1182/blood-2005-03-1320

[bib5] Lau WWY, Hannah R, Green AR, Göttgens B. The JAK-STAT signaling pathway is differentially activated in CALR-positive compared with JAK2V617F-positive ET patients. Blood 2015; 125: 1679–1681. 25745188 10.1182/blood-2014-12-618074 PMC4471770

[bib6] Mascarenhas J, Roper N, Chaurasia P, Hoffman R. Epigenetic abnormalities in myeloproliferative neoplasms: a target for novel therapeutic strategies. Clin Epigenetics 2011; 2: 197–212. 22704337 10.1007/s13148-011-0050-6 PMC3365400

[bib7] Cross NCP. Genetic and epigenetic complexity in myeloproliferative neoplasms. Hematol Am Soc Hematol Educ Program 2011; 2011: 208–214. 10.1182/asheducation-2011.1.208 22160036

[bib8] James C, Ugo V, Le Couédic J-P, Staerk J, Delhommeau F, Lacout C et al. A unique clonal JAK2 mutation leading to constitutive signalling causes polycythaemia vera. Nature 2005; 434: 1144–1148. 15793561 10.1038/nature03546

[bib9] Kralovics R, Passamonti F, Buser AS, Teo S-S, Tiedt R, Passweg JR et al. A gain-of-function mutation of JAK2 in myeloproliferative disorders. N Engl J Med 2005; 352: 1779–1790. 15858187 10.1056/NEJMoa051113

[bib10] Zhao R, Xing S, Li Z, Fu X, Li Q, Krantz SB et al. Identification of an acquired JAK2 mutation in polycythemia vera. J Biol Chem 2005; 280: 22788–22792. 15863514 10.1074/jbc.C500138200 PMC1201515

[bib11] Klampfl T, Harutyunyan A, Berg T, Gisslinger B, Schalling M, Bagienski K et al. Genome integrity of myeloproliferative neoplasms in chronic phase and during disease progression. Blood 2011; 118: 167–176. 21531982 10.1182/blood-2011-01-331678

[bib12] Vannucchi AM, Biamonte F. Epigenetics and mutations in chronic myeloproliferative neoplasms. Haematologica 2011; 96: 1398–1402. 21972209 10.3324/haematol.2011.052068 PMC3186298

[bib13] Nangalia J, Massie CE, Baxter EJ, Nice FL, Gundem G, Wedge DC et al. Somatic CALR mutations in myeloproliferative neoplasms with nonmutated JAK2. N Engl J Med 2013; 369: 2391–2405. 24325359 10.1056/NEJMoa1312542 PMC3966280

[bib14] Klampfl T, Gisslinger H, Harutyunyan AS, Nivarthi H, Rumi E, Milosevic JD et al. Somatic mutations of calreticulin in myeloproliferative neoplasms. N Engl J Med 2013; 369: 2379–2390. 24325356 10.1056/NEJMoa1311347

[bib15] Tefferi A. Novel mutations and their functional and clinical relevance in myeloproliferative neoplasms: JAK2, MPL, TET2, ASXL1, CBL, IDH and IKZF1. Leukemia 2010; 24: 1128–1138. 20428194 10.1038/leu.2010.69 PMC3035972

[bib16] Ortmann CA, Kent DG, Nangalia J, Silber Y, Wedge DC, Grinfeld J et al. Effect of mutation order on myeloproliferative neoplasms. N Engl J Med 2015; 372: 601–612. 25671252 10.1056/NEJMoa1412098 PMC4660033

[bib17] Jaiswal S, Fontanillas P, Flannick J, Manning A, Grauman PV, Mar BG et al. Age-related clonal hematopoiesis associated with adverse outcomes. N Engl J Med 2014; 371: 2488–2498. 25426837 10.1056/NEJMoa1408617 PMC4306669

[bib18] Xie M, Lu C, Wang J, McLellan MD, Johnson KJ, Wendl MC et al. Age-related mutations associated with clonal hematopoietic expansion and malignancies. Nat Med 2014; 20: 1472–1478. 25326804 10.1038/nm.3733 PMC4313872

[bib19] Zhan H, Cardozo C, Raza A. MicroRNAs in myeloproliferative neoplasms. Br J Haematol 2013; 161: 471–483. 23432162 10.1111/bjh.12276 PMC5181108

[bib20] Zhang L, Sankaran VG, Lodish HF. MicroRNAs in erythroid and megakaryocytic differentiation and megakaryocyte-erythroid progenitor lineage commitment. Leukemia 2012; 26: 2310–2316. 22617791 10.1038/leu.2012.137 PMC3739046

[bib21] Báez A, Martín-Antonio B, Piruat JI, Barbado MV, Prats C, Álvarez-Laderas I et al. Gene and miRNA expression profiles of hematopoietic progenitor cells vary depending on their origin. Biol Blood Marrow Transplant J Am Soc Blood Marrow Transplant 2014; 20: 630–639. 10.1016/j.bbmt.2014.01.022 24462744

[bib22] Raghavachari N, Liu P, Barb JJ, Yang Y, Wang R, Nguyen QT et al. Integrated analysis of miRNA and mRNA during differentiation of human CD34+ cells delineates the regulatory roles of microRNA in hematopoiesis. Exp Hematol 2014; 42: 14–27–2. 24139908 10.1016/j.exphem.2013.10.003 PMC3878057

[bib23] Guglielmelli P, Tozzi L, Bogani C, Iacobucci I, Ponziani V, Martinelli G et al. Overexpression of microRNA-16-2 contributes to the abnormal erythropoiesis in polycythemia vera. Blood 2011; 117: 6923–6927. 21527532 10.1182/blood-2010-09-306506

[bib24] Norfo R, Zini R, Pennucci V, Bianchi E, Salati S, Guglielmelli P et al. miRNA-mRNA integrative analysis in primary myelofibrosis CD34+ cells: role of miR-155/JARID2 axis in abnormal megakaryopoiesis. Blood 2014; 124: e21–e32. 25097177 10.1182/blood-2013-12-544197 PMC4186546

[bib25] Bortoluzzi S, Bisognin A, Biasiolo M, Guglielmelli P, Biamonte F, Norfo R et al. Characterization and discovery of novel miRNAs and moRNAs in JAK2V617F-mutated SET2 cells. Blood 2012; 119: e120–e130. 22223824 10.1182/blood-2011-07-368001

[bib26] Guglielmelli P, Bisognin A, Saccoman C, Mannarelli C, Coppe A, Vannucchi AM et al. Small RNA sequencing uncovers new miRNAs and moRNAs differentially expressed in normal and primary myelofibrosis CD34+ cells. PloS One 2015; 10: e0140445. 26468945 10.1371/journal.pone.0140445 PMC4607157

[bib27] Ha J-S, Jung H-R. Up-regulation of microRNA 146b is associated with myelofibrosis in myeloproliferative neoplasms. Ann Clin Lab Sci 2015; 45: 308–314. 26116595

[bib28] Bartalucci N, Guglielmelli P, Vannucchi AM. Rationale for targeting the PI3K/Akt/mTOR pathway in myeloproliferative neoplasms. Clin Lymphoma Myeloma Leuk 2013; 13(Suppl 2): S307–S309. 24290217 10.1016/j.clml.2013.07.011

[bib29] Mascarenhas J. Rationale for combination therapy in myelofibrosis. Best Pract Res Clin Haematol 2014; 27: 197–208. 25189730 10.1016/j.beha.2014.07.009

[bib30] Scherer A, Dai M, Meng F. Impact of experimental noise and annotation imprecision on data quality in microarray experiments. Methods Mol Biol Clifton NJ 2013; 972: 155–176. 10.1007/978-1-60327-337-4_10 23385537

[bib31] Calura E, Martini P, Sales G, Beltrame L, Chiorino G, D'Incalci M et al. Wiring miRNAs to pathways: a topological approach to integrate miRNA and mRNA expression profiles. Nucleic Acids Res 2014; 42: e96. 24803669 10.1093/nar/gku354 PMC4066781

[bib32] Kanehisa M, Goto S. KEGG: kyoto encyclopedia of genes and genomes. Nucleic Acids Res 2000; 28: 27–30. 10592173 10.1093/nar/28.1.27 PMC102409

[bib33] Sales G, Calura E, Cavalieri D, Romualdi C. graphite—a Bioconductor package to convert pathway topology to gene network. BMC Bioinformatics 2012; 13: 20. 22292714 10.1186/1471-2105-13-20 PMC3296647

[bib34] Hsu S-D, Tseng Y-T, Shrestha S, Lin Y-L, Khaleel A, Chou C-H et al. miRTarBase update 2014: an information resource for experimentally validated miRNA-target interactions. Nucleic Acids Res 2014 42: D78–D85. 10.1093/nar/gkt1266 PMC3965058 24304892

[bib35] Xiao F, Zuo Z, Cai G, Kang S, Gao X, Li T. miRecords: an integrated resource for microRNA-target interactions. Nucleic Acids Res 2009; 37: D105–D110. 18996891 10.1093/nar/gkn851 PMC2686554

[bib36] Bisognin A, Sales G, Coppe A, Bortoluzzi S, Romualdi C. MAGIA2: from miRNA and genes expression data integrative analysis to microRNA-transcription factor mixed regulatory circuits (2012 update). Nucleic Acids Res 2012; 40: W13–W21. 22618880 10.1093/nar/gks460 PMC3394337

[bib37] Shannon P, Markiel A, Ozier O, Baliga NS, Wang JT, Ramage D et al. Cytoscape: a software environment for integrated models of biomolecular interaction networks. Genome Res 2003; 13: 2498–2504. 14597658 10.1101/gr.1239303 PMC403769

[bib38] MacLellan SA, Lawson J, Baik J, Guillaud M, CF-Y Poh, Garnis C. Differential expression of miRNAs in the serum of patients with high-risk oral lesions. Cancer Med 2012; 1: 268–274. 23342275 10.1002/cam4.17 PMC3544450

[bib39] Inui M, Martello G, Piccolo S. MicroRNA control of signal transduction. Nat Rev Mol Cell Biol 2010; 11: 252–263. 20216554 10.1038/nrm2868

[bib40] Guglielmelli P, Barosi G, Rambaldi A, Marchioli R, Masciulli A, Tozzi L et al. Safety and efficacy of everolimus, a mTOR inhibitor, as single agent in a phase 1/2 study in patients with myelofibrosis. Blood 2011; 118: 2069–2076. 21725052 10.1182/blood-2011-01-330563 PMC3365876

[bib41] Bogani C, Bartalucci N, Martinelli S, Tozzi L, Guglielmelli P, Bosi A et al. mTOR inhibitors alone and in combination with JAK2 inhibitors effectively inhibit cells of myeloproliferative neoplasms. PloS One 2013; 8: e54826. 23382981 10.1371/journal.pone.0054826 PMC3561413

[bib42] Bartalucci N, Tozzi L, Bogani C, Martinelli S, Rotunno G, Villeval J-L et al. Co-targeting the PI3K/mTOR and JAK2 signalling pathways produces synergistic activity against myeloproliferative neoplasms. J Cell Mol Med 2013; 17: 1385–1396. 24237791 10.1111/jcmm.12162 PMC4117551

[bib43] Nayak RC, Chang K-H, Vaitinadin N-S, Cancelas JA. Rho GTPases control specific cytoskeleton-dependent functions of hematopoietic stem cells. Immunol Rev 2013; 256: 255–268. 24117826 10.1111/imr.12119 PMC3830525

[bib44] Vega FM, Ridley AJ. Rho GTPases in cancer cell biology. FEBS Lett 2008; 582: 2093–2101. 18460342 10.1016/j.febslet.2008.04.039

[bib45] Yang FC, Atkinson SJ, Gu Y, Borneo JB, Roberts AW, Zheng Y et al. Rac and Cdc42 GTPases control hematopoietic stem cell shape, adhesion, migration, and mobilization. Proc Natl Acad Sci USA 2001; 98: 5614–5618. 11320224 10.1073/pnas.101546898 PMC33261

[bib46] Yang L, Wang L, Kalfa TA, Cancelas JA, Shang X, Pushkaran S et al. Cdc42 critically regulates the balance between myelopoiesis and erythropoiesis. Blood 2007; 110: 3853–3861. 17702896 10.1182/blood-2007-03-079582 PMC2190607

[bib47] Li Z, Dong X, Dong X, Wang Z, Liu W, Deng N et al. Regulation of PTEN by Rho small GTPases. Nat Cell Biol 2005; 7: 399–404. 15793569 10.1038/ncb1236

[bib48] Jang YH, Min DS. The hydrophobic amino acids involved in the interdomain association of phospholipase D1 regulate the shuttling of phospholipase D1 from vesicular organelles into the nucleus. Exp Mol Med 2012; 44: 571–577. 22824913 10.3858/emm.2012.44.10.065 PMC3490078

[bib49] Jang YH, Min DS. Intermolecular association between caspase-mediated cleavage fragments of phospholipase D1 protects against apoptosis. Int J Biochem Cell Biol 2012; 44: 358–365. 22108201 10.1016/j.biocel.2011.11.010

[bib50] Jang J-H, Lee CS, Hwang D, Ryu SH. Understanding of the roles of phospholipase D and phosphatidic acid through their binding partners. Prog Lipid Res 2012; 51: 71–81. 22212660 10.1016/j.plipres.2011.12.003

[bib51] Norton LJ, Zhang Q, Saqib KM, Schrewe H, Macura K, Anderson KE et al. PLD1 rather than PLD2 regulates phorbol-ester-, adhesion-dependent and Fc{gamma}-receptor-stimulated ROS production in neutrophils. J Cell Sci 2011; 124: 1973–1983. 21610093 10.1242/jcs.082008 PMC3104032

[bib52] Ganesan R, Mahankali M, Alter G, Gomez-Cambronero J. Two sites of action for PLD2 inhibitors: the enzyme catalytic center and an allosteric, phosphoinositide biding pocket. Biochim Biophys Acta BBA - Mol Cell Biol Lipids 2015; 1851: 261–272. 10.1016/j.bbalip.2014.12.007 PMC4312721 25532944

[bib53] Mahankali M, Henkels KM, Gomez-Cambronero J. A GEF-to-phospholipase molecular switch caused by phosphatidic acid, Rac and JAK tyrosine kinase that explains leukocyte cell migration. J Cell Sci 2013; 126: 1416–1428. 23378025 10.1242/jcs.117960 PMC3644142

[bib54] Gomez-Cambronero J. Biochemical and cellular implications of a dual lipase-GEF function of phospholipase D2 (PLD2). J Leukoc Biol 2012; 92: 461–467. 22750546 10.1189/jlb.0212073 PMC3427609

[bib55] Zhang Y, Du G. Phosphatidic acid signaling regulation of Ras superfamily of small guanosine triphosphatases. Biochim Biophys Acta 2009; 1791: 850–855. 19540930 10.1016/j.bbalip.2009.05.013 PMC2739575

[bib56] Buxhofer-Ausch V, Olcaydu D, Gisslinger B, Schalling M, Frantal S, Thiele J et al. Decanucleotide insertion polymorphism of F7 significantly influences the risk of thrombosis in patients with essential thrombocythemia. Eur J Haematol 2014; 93: 103–111. 24617727 10.1111/ejh.12307

[bib57] He H, Xu Y-J, Yin J-Y, Li X, Qu J, Xu X-J et al. Association of nitric oxide synthase 3 (NOS3) 894 G>T polymorphism with prognostic outcomes of anthracycline in Chinese patients with acute myeloid leukaemia. Clin Exp Pharmacol Physiol 2014; 41: 400–407. 24684492 10.1111/1440-1681.12235

[bib58] Boissinot M, Vilaine M, Hermouet S. The hepatocyte growth factor (HGF)/met axis: a neglected target in the treatment of chronic myeloproliferative neoplasms? Cancers 2014; 6: 1631–1669. 25119536 10.3390/cancers6031631 PMC4190560

[bib59] Spencer JA, Ferraro F, Roussakis E, Klein A, Wu J, Runnels JM et al. Direct measurement of local oxygen concentration in the bone marrow of live animals. Nature 2014; 508: 269–273. 24590072 10.1038/nature13034 PMC3984353

[bib60] Lu Y-C, Weng W-C, Lee H. Functional roles of calreticulin in cancer biology. BioMed Res Int 2015; 2015: 526524. 25918716 10.1155/2015/526524 PMC4396016

[bib61] Britschgi C, Jenal M, Rizzi M, Mueller BU, Torbett BE, Andres A-C et al. HIC1 tumour suppressor gene is suppressed in acute myeloid leukaemia and induced during granulocytic differentiation. Br J Haematol 2008; 141: 179–187. 18318772 10.1111/j.1365-2141.2008.06992.x

[bib62] Melki JR, Vincent PC, Clark SJ. Cancer-specific region of hypermethylation identified within the HIC1 putative tumour suppressor gene in acute myeloid leukaemia. Leukemia 1999; 13: 877–883. 10360376 10.1038/sj.leu.2401401

[bib63] Roodink I, Verrijp K, Raats J, Leenders WPJ. Plexin D1 is ubiquitously expressed on tumor vessels and tumor cells in solid malignancies. BMC Cancer 2009; 9: 297. 19703316 10.1186/1471-2407-9-297 PMC2739226

[bib64] Luchino J, Hocine M, Amoureux M-C, Gibert B, Bernet A, Royet A et al. Semaphorin 3E suppresses tumor cell death triggered by the plexin D1 dependence receptor in metastatic breast cancers. Cancer Cell 2013; 24: 673–685. 24139859 10.1016/j.ccr.2013.09.010

[bib65] Tamagnone L, Rehman M. To die or not to die: Sema3E rules the game. Cancer Cell 2013; 24: 564–566. 24229706 10.1016/j.ccr.2013.10.010

[bib66] Yan Y, Tsukamoto O, Nakano A, Kato H, Kioka H, Ito N et al. Augmented AMPK activity inhibits cell migration by phosphorylating the novel substrate Pdlim5. Nat Commun 2015; 6: 6137. 25635515 10.1038/ncomms7137 PMC4317497

