# Supplementary material for: A data-driven network model of primary myelofibrosis: transcriptional and post-transcriptional alterations in CD34+ cells
Source: Blood Cancer J. 2016 Jun 24;6(6):e439–. doi: 10.1038/bcj.2016.47 (PMC5141361; doi:10.1038/bcj.2016.47)

**A data-driven network model of PMF: transcriptional and post-transcriptional alterations in PMF CD34+ cells**

**SUPPLEMENTARY RESULTS**

1. **Micrographite results of Primary Myelofibrosis samples compared with bone marrow form healthy controls.**

**Table S1. Micrographite whole pathway analysis results**

| **Pathway Name** | **p-value mean test** | **p-value variance test** | **adjusted**  **p-value mean test** | **adjusted**  **p-value variance test** |
| --- | --- | --- | --- | --- |
| Acute myeloid leukemia | 0 | 0 | 0 | 0 |
| Adherens junction | 0 | 0 | 0 | 0 |
| Adipocytokine signaling pathway | 0 | 0 | 0 | 0 |
| African trypanosomiasis | 0 | 0 | 0 | 0 |
| Alanine, aspartate and glutamate metabolism | 0 | 0 | 0 | 0 |
| Alcoholism | 0 | 0 | 0 | 0 |
| Aldosterone-regulated sodium reabsorption | 0 | 0 | 0 | 0 |
| Allograft rejection | 0 | 0 | 0 | 0 |
| Alzheimer's disease | 0 | 0 | 0 | 0 |
| Amino sugar and nucleotide sugar metabolism | 0 | 0 | 0 | 0 |
| Aminoacyl-tRNA biosynthesis | 0 | 0 | 0 | 0 |
| Amoebiasis | 0 | 0 | 0 | 0 |
| Amphetamine addiction | 0 | 0 | 0 | 0 |
| Amyotrophic lateral sclerosis (ALS) | 0 | 0 | 0 | 0 |
| Antigen processing and presentation | 0 | 0 | 0 | 0 |
| Apoptosis | 0 | 0 | 0 | 0 |
| Arginine and proline metabolism | 0 | 0 | 0 | 0 |
| Arrhythmogenic right ventricular cardiomyopathy (ARVC) | 0 | 0 | 0 | 0 |
| Asthma | 0 | 0 | 0 | 0 |
| Autoimmune thyroid disease | 0 | 0 | 0 | 0 |
| Axon guidance | 0 | 0 | 0 | 0 |
| B cell receptor signaling pathway | 0 | 0 | 0 | 0 |
| Bacterial invasion of epithelial cells | 0 | 0 | 0 | 0 |
| Basal cell carcinoma | 0 | 0 | 0 | 0 |
| Bile secretion | 0 | 0 | 0 | 0 |
| Bladder cancer | 0 | 0 | 0 | 0 |
| Butirosin and neomycin biosynthesis | 0 | 0 | 0 | 0 |
| Cardiac muscle contraction | 0 | 0 | 0 | 0 |
| Cell adhesion molecules (CAMs) | 0 | 0 | 0 | 0 |
| Chemical carcinogenesis | 0 | 0 | 0 | 0 |
| Chemokine signaling pathway | 0 | 0 | 0 | 0 |
| Cholinergic synapse | 0 | 0 | 0 | 0 |
| Chronic myeloid leukemia | 0 | 0 | 0 | 0 |
| Circadian entrainment | 0 | 0 | 0 | 0 |
| Circadian rhythm | 0 | 0 | 0 | 0 |
| Colorectal cancer | 0 | 0 | 0 | 0 |
| Complement and coagulation cascades | 0 | 0 | 0 | 0 |
| Cyanoamino acid metabolism | 0 | 0 | 0 | 0 |
| Cysteine and methionine metabolism | 0 | 0 | 0 | 0 |
| Cytosolic DNA-sensing pathway | 0 | 0 | 0 | 0 |
| Dilated cardiomyopathy | 0 | 0 | 0 | 0 |
| Dopaminergic synapse | 0 | 0 | 0 | 0 |
| Dorso-ventral axis formation | 0 | 0 | 0 | 0 |
| Drug metabolism - cytochrome P450 | 0 | 0 | 0 | 0 |
| ECM-receptor interaction | 0 | 0 | 0 | 0 |
| Endocrine and other factor-regulated calcium reabsorption | 0 | 0 | 0 | 0 |
| Endometrial cancer | 0 | 0 | 0 | 0 |
| Epithelial cell signaling in Helicobacter pylori infection | 0 | 0 | 0 | 0 |
| Epstein-Barr virus infection | 0 | 0 | 0 | 0 |
| ErbB signaling pathway | 0 | 0 | 0 | 0 |
| Ether lipid metabolism | 0 | 0 | 0 | 0 |
| Fatty acid elongation | 0 | 0 | 0 | 0 |
| Fatty acid metabolism | 0 | 0 | 0 | 0 |
| Fc epsilon RI signaling pathway | 0 | 0 | 0 | 0 |
| Fc gamma R-mediated phagocytosis | 0 | 0 | 0 | 0 |
| Fructose and mannose metabolism | 0 | 0 | 0 | 0 |
| GABAergic synapse | 0 | 0 | 0 | 0 |
| Galactose metabolism | 0 | 0 | 0 | 0 |
| Gap junction | 0 | 0 | 0 | 0 |
| Gastric acid secretion | 0 | 0 | 0 | 0 |
| Glioma | 0 | 0 | 0 | 0 |
| Glutamatergic synapse | 0 | 0 | 0 | 0 |
| Glutathione metabolism | 0 | 0 | 0 | 0 |
| Glycerolipid metabolism | 0 | 0 | 0 | 0 |
| Glycerophospholipid metabolism | 0 | 0 | 0 | 0 |
| Glycine, serine and threonine metabolism | 0 | 0 | 0 | 0 |
| Glycolysis / Gluconeogenesis | 0 | 0 | 0 | 0 |
| Glycosaminoglycan biosynthesis - chondroitin sulfate / dermatan sulfate | 0 | 0 | 0 | 0 |
| Glycosaminoglycan degradation | 0 | 0 | 0 | 0 |
| Glycosylphosphatidylinositol(GPI)-anchor biosynthesis | 0 | 0 | 0 | 0 |
| GnRH signaling pathway | 0 | 0 | 0 | 0 |
| Graft-versus-host disease | 0 | 0 | 0 | 0 |
| HIF-1 signaling pathway | 0 | 0 | 0 | 0 |
| Hedgehog signaling pathway | 0 | 0 | 0 | 0 |
| Hepatitis C | 0 | 0 | 0 | 0 |
| Herpes simplex infection | 0 | 0 | 0 | 0 |
| Hippo signaling pathway | 0 | 0 | 0 | 0 |
| Histidine metabolism | 0 | 0 | 0 | 0 |
| Hypertrophic cardiomyopathy (HCM) | 0 | 0 | 0 | 0 |
| Influenza A | 0 | 0 | 0 | 0 |
| Inositol phosphate metabolism | 0 | 0 | 0 | 0 |
| Insulin signaling pathway | 0 | 0 | 0 | 0 |
| Intestinal immune network for IgA production | 0 | 0 | 0 | 0 |
| Jak-STAT signaling pathway | 0 | 0 | 0 | 0 |
| Legionellosis | 0 | 0 | 0 | 0 |
| Leishmaniasis | 0 | 0 | 0 | 0 |
| Long-term depression | 0 | 0 | 0 | 0 |
| Long-term potentiation | 0 | 0 | 0 | 0 |
| Lysine biosynthesis | 0 | 0 | 0 | 0 |
| Lysine degradation | 0 | 0 | 0 | 0 |
| Malaria | 0 | 0 | 0 | 0 |
| Measles | 0 | 0 | 0 | 0 |
| Melanogenesis | 0 | 0 | 0 | 0 |
| Melanoma | 0 | 0 | 0 | 0 |
| Mineral absorption | 0 | 0 | 0 | 0 |
| Morphine addiction | 0 | 0 | 0 | 0 |
| Mucin type O-Glycan biosynthesis | 0 | 0 | 0 | 0 |
| N-Glycan biosynthesis | 0 | 0 | 0 | 0 |
| NF-kappa B signaling pathway | 0 | 0 | 0 | 0 |
| NOD-like receptor signaling pathway | 0 | 0 | 0 | 0 |
| Natural killer cell mediated cytotoxicity | 0 | 0 | 0 | 0 |
| Neuroactive ligand-receptor interaction | 0 | 0 | 0 | 0 |
| Neurotrophin signaling pathway | 0 | 0 | 0 | 0 |
| Nicotinate and nicotinamide metabolism | 0 | 0 | 0 | 0 |
| Non-small cell lung cancer | 0 | 0 | 0 | 0 |
| Notch signaling pathway | 0 | 0 | 0 | 0 |
| Oocyte meiosis | 0 | 0 | 0 | 0 |
| Osteoclast differentiation | 0 | 0 | 0 | 0 |
| Oxidative phosphorylation | 0 | 0 | 0 | 0 |
| PPAR signaling pathway | 0 | 0 | 0 | 0 |
| Pancreatic cancer | 0 | 0 | 0 | 0 |
| Pancreatic secretion | 0 | 0 | 0 | 0 |
| Parkinson's disease | 0 | 0 | 0 | 0 |
| Pathogenic Escherichia coli infection | 0 | 0 | 0 | 0 |
| Pentose phosphate pathway | 0 | 0 | 0 | 0 |
| Pertussis | 0 | 0 | 0 | 0 |
| Phenylalanine metabolism | 0 | 0 | 0 | 0 |
| Phosphatidylinositol signaling system | 0 | 0 | 0 | 0 |
| Phototransduction | 0 | 0 | 0 | 0 |
| Porphyrin and chlorophyll metabolism | 0 | 0 | 0 | 0 |
| Prion diseases | 0 | 0 | 0 | 0 |
| Progesterone-mediated oocyte maturation | 0 | 0 | 0 | 0 |
| Prostate cancer | 0 | 0 | 0 | 0 |
| Proximal tubule bicarbonate reclamation | 0 | 0 | 0 | 0 |
| RIG-I-like receptor signaling pathway | 0 | 0 | 0 | 0 |
| Renal cell carcinoma | 0 | 0 | 0 | 0 |
| Retinol metabolism | 0 | 0 | 0 | 0 |
| Retrograde endocannabinoid signaling | 0 | 0 | 0 | 0 |
| Rheumatoid arthritis | 0 | 0 | 0 | 0 |
| Riboflavin metabolism | 0 | 0 | 0 | 0 |
| Salivary secretion | 0 | 0 | 0 | 0 |
| Salmonella infection | 0 | 0 | 0 | 0 |
| Selenocompound metabolism | 0 | 0 | 0 | 0 |
| Serotonergic synapse | 0 | 0 | 0 | 0 |
| Small cell lung cancer | 0 | 0 | 0 | 0 |
| Sphingolipid metabolism | 0 | 0 | 0 | 0 |
| Staphylococcus aureus infection | 0 | 0 | 0 | 0 |
| Starch and sucrose metabolism | 0 | 0 | 0 | 0 |
| Sulfur metabolism | 0 | 0 | 0 | 0 |
| Synaptic vesicle cycle | 0 | 0 | 0 | 0 |
| Synthesis and degradation of ketone bodies | 0 | 0 | 0 | 0 |
| Systemic lupus erythematosus | 0 | 0 | 0 | 0 |
| T cell receptor signaling pathway | 0 | 0 | 0 | 0 |
| TGF-beta signaling pathway | 0 | 0 | 0 | 0 |
| Terpenoid backbone biosynthesis | 0 | 0 | 0 | 0 |
| Thyroid cancer | 0 | 0 | 0 | 0 |
| Tight junction | 0 | 0 | 0 | 0 |
| Toll-like receptor signaling pathway | 0 | 0 | 0 | 0 |
| Toxoplasmosis | 0 | 0 | 0 | 0 |
| Transcriptional misregulation in cancer | 0 | 0 | 0 | 0 |
| Tryptophan metabolism | 0 | 0 | 0 | 0 |
| Tuberculosis | 0 | 0 | 0 | 0 |
| Type I diabetes mellitus | 0 | 0 | 0 | 0 |
| Type II diabetes mellitus | 0 | 0 | 0 | 0 |
| Tyrosine metabolism | 0 | 0 | 0 | 0 |
| Ubiquinone and other terpenoid-quinone biosynthesis | 0 | 0 | 0 | 0 |
| VEGF signaling pathway | 0 | 0 | 0 | 0 |
| Valine, leucine and isoleucine degradation | 0 | 0 | 0 | 0 |
| Vascular smooth muscle contraction | 0 | 0 | 0 | 0 |
| Vasopressin-regulated water reabsorption | 0 | 0 | 0 | 0 |
| Vibrio cholerae infection | 0 | 0 | 0 | 0 |
| Viral carcinogenesis | 0 | 0 | 0 | 0 |
| Viral myocarditis | 0 | 0 | 0 | 0 |
| Wnt signaling pathway | 0 | 0 | 0 | 0 |
| beta-Alanine metabolism | 0 | 0 | 0 | 0 |
| mTOR signaling pathway | 0 | 0 | 0 | 0 |
| p53 signaling pathway | 0 | 0 | 0 | 0 |
| Arachidonic acid metabolism | 0 | 0.01 | 0 | 0.011396648 |
| Citrate cycle (TCA cycle) | 0 | 0.01 | 0 | 0.011396648 |
| Glycosphingolipid biosynthesis - globo series | 0 | 0.01 | 0 | 0.011396648 |
| Glycosphingolipid biosynthesis - lacto and neolacto series | 0 | 0.01 | 0 | 0.011396648 |
| Glyoxylate and dicarboxylate metabolism | 0 | 0.01 | 0 | 0.011396648 |
| Huntington's disease | 0 | 0.01 | 0 | 0.011396648 |
| Metabolism of xenobiotics by cytochrome P450 | 0 | 0.01 | 0 | 0.011396648 |
| One carbon pool by folate | 0 | 0.01 | 0 | 0.011396648 |
| Pantothenate and CoA biosynthesis | 0 | 0.01 | 0 | 0.011396648 |
| Pyruvate metabolism | 0 | 0.01 | 0 | 0.011396648 |
| Regulation of actin cytoskeleton | 0 | 0.01 | 0 | 0.011396648 |
| Butanoate metabolism | 0 | 0.02 | 0 | 0.021935484 |
| D-Glutamine and D-glutamate metabolism | 0 | 0.02 | 0 | 0.021935484 |
| Fat digestion and absorption | 0 | 0.02 | 0 | 0.021935484 |
| Steroid biosynthesis | 0 | 0.02 | 0 | 0.021935484 |
| Steroid hormone biosynthesis | 0 | 0.02 | 0 | 0.021935484 |
| Taste transduction | 0 | 0.02 | 0 | 0.021935484 |
| Vitamin B6 metabolism | 0 | 0.02 | 0 | 0.021935484 |
| Carbohydrate digestion and absorption | 0 | 0.03 | 0 | 0.032380952 |
| Primary bile acid biosynthesis | 0 | 0.03 | 0 | 0.032380952 |
| Folate biosynthesis | 0 | 0.04 | 0 | 0.042722513 |
| Lipoic acid metabolism | 0 | 0.04 | 0 | 0.042722513 |
| Pyrimidine metabolism | 0 | 0.05 | 0 | 0.053125 |
| Phenylalanine, tyrosine and tryptophan biosynthesis | 0 | 0.06 | 0 | 0.063419689 |
| Purine metabolism | 0 | 0.07 | 0 | 0.073230769 |
| alpha-Linolenic acid metabolism | 0 | 0.07 | 0 | 0.073230769 |
| Caffeine metabolism | 0 | 0.09 | 0 | 0.093673469 |
| Biotin metabolism | 0.03 | 0.03 | 0.030447761 | 0.032380952 |

**Table S2. Results of the Micrographite analysis of the paths in the pathways.** The paths presented in the following table have been selected to compose the meta-pathway.

| **Name of the pathway** | **Max Score** | **Involved Genes and miRNAs** |
| --- | --- | --- |
| HIF-1 signaling pathway | 192.7 | 1536, 27035, 50508, 5335, 5336, 5335, 5336, 5578, 5579, 5582, 10000, 112399, 207, 208, 2475, 54583, 5578, 5579, 5582, hsa-miR-122-5p, hsa-miR-20a-5p, hsa-miR-373-3p, hsa-miR-99a-5p, 10000, 112399, 1956, 2064, 207, 208, 2475, 3480, 3643, 54583, hsa-miR-122-5p, hsa-miR-145-5p, hsa-miR-20a-5p, hsa-miR-28-5p, hsa-miR-34a-5p, hsa-miR-373-3p, hsa-miR-424-5p, hsa-miR-99a-5p, 10000, 112399, 1956, 2064, 207, 208, 3480, 3643, 54583, hsa-miR-122-5p, hsa-miR-126-3p, hsa-miR-145-5p, hsa-miR-20a-5p, hsa-miR-221-3p, hsa-miR-28-5p, hsa-miR-29a-3p, hsa-miR-29b-3p, hsa-miR-29c-3p, hsa-miR-34a-5p, hsa-miR-373-3p, hsa-miR-424-5p, hsa-miR-519d, hsa-miR-99a-5p, 112399, 1387, 1956, 2033, 2064, 3091, 3480, 3643, 405, 54583, hsa-miR-107, hsa-miR-122-5p, hsa-miR-125b-5p, hsa-miR-126-3p, hsa-miR-145-5p, hsa-miR-146a-5p, hsa-miR-146b-5p, hsa-miR-20a-5p, hsa-miR-21-5p, hsa-miR-221-3p, hsa-miR-28-5p, hsa-miR-29a-3p, hsa-miR-29b-3p, hsa-miR-29c-3p, hsa-miR-34a-5p, hsa-miR-373-3p, hsa-miR-424-5p, hsa-miR-519d, hsa-miR-99a-5p, 1387, 1956, 2033, 2064, 3091, 3480, 3643, 405, hsa-miR-107, hsa-miR-122-5p, hsa-miR-125a-5p, hsa-miR-125b-5p, hsa-miR-126-3p, hsa-miR-145-5p, hsa-miR-146a-5p, hsa-miR-146b-5p, hsa-miR-15a-5p, hsa-miR-15b-5p, hsa-miR-16-5p, hsa-miR-17-5p, hsa-miR-195-5p, hsa-miR-20a-5p, hsa-miR-21-5p, hsa-miR-221-3p, hsa-miR-28-5p, hsa-miR-29a-3p, hsa-miR-29b-3p, hsa-miR-29c-3p, hsa-miR-34a-5p, hsa-miR-34b-5p, hsa-miR-373-3p, hsa-miR-519d, hsa-miR-99a-5p, 1387, 1956, 2033, 2064, 3091, 3480, 3643, 405, hsa-miR-122-5p, hsa-miR-125a-5p, hsa-miR-125b-5p, hsa-miR-145-5p, hsa-miR-146a-5p, hsa-miR-15a-5p, hsa-miR-15b-5p, hsa-miR-16-5p, hsa-miR-17-5p, hsa-miR-181a-5p, hsa-miR-192-5p, hsa-miR-195-5p, hsa-miR-20a-5p, hsa-miR-21-5p, hsa-miR-221-3p, hsa-miR-29a-3p, hsa-miR-29b-3p, hsa-miR-29c-3p, hsa-miR-34a-5p, hsa-miR-34b-5p, hsa-miR-99a-5p, 1387, 2033, 3091, 405, hsa-miR-125b-5p, hsa-miR-143-3p, hsa-miR-145-5p, hsa-miR-15a-5p, hsa-miR-15b-5p, hsa-miR-16-5p, hsa-miR-17-5p, hsa-miR-181a-5p, hsa-miR-192-5p, hsa-miR-195-5p, hsa-miR-20a-5p, hsa-miR-21-5p, hsa-miR-29a-3p, hsa-miR-29b-3p, hsa-miR-29c-3p, hsa-miR-34a-5p, hsa-miR-34b-5p, hsa-miR-99a-5p, 1387, 2033, 3091, 405, 5054, hsa-miR-143-3p, hsa-miR-145-5p, hsa-miR-301a-3p, hsa-miR-30c-5p, hsa-miR-99a-5p, 1387, 2033, 3091, 3099, 405, hsa-miR-143-3p, 1387, 2033, 3091, 405, 7037, hsa-miR-22-3p, hsa-miR-320a, 1387, 2033, 3091, 405, 4846, hsa-miR-1244, 1387, 2033, 3091, 405, 5163, hsa-miR-375, 1387, 2033, 3091, 405, 815, 816, 817, 818, hsa-miR-324-3p, 1387, 2023, 2033, 3091, 405, 1387, 2026, 2033, 3091, 405, 1387, 2027, 2033, 3091, 405, 1387, 2033, 2056, 3091, 405, 1387, 2033, 284, 3091, 405, 1387, 2033, 285, 3091, 405, 1387, 2033, 3091, 3098, 405, 1387, 2033, 3091, 3101, 405, 1387, 2033, 3091, 3939, 405, 1387, 2033, 3091, 405, 4055, 1387, 2033, 3091, 405, 4843, 1387, 2033, 3091, 405, 4878, 1387, 2033, 3091, 405, 51378, 1387, 2033, 3091, 405, 5207, 1387, 2033, 3091, 405, 5208, 1387, 2033, 3091, 405, 5209, 1387, 2033, 3091, 405, 5210, 1387, 2033, 3091, 405, 5211, 1387, 2033, 3091, 405, 5228, 1387, 2033, 3091, 405, 5230, 1387, 2033, 3091, 405, 6513, 1387, 2033, 3091, 405, 7010, 1387, 2033, 3091, 405, 7018, 1387, 2033, 3091, 405, 7076, 1387, 2033, 3091, 405, 7423, 1387, 2033, 3091, 405, 7424, 1387, 2033, 3091, 405, 80201 |
| PPAR signaling pathway | 144.5 | 5465, 6256, 6257, 6258, hsa-miR-10b-5p, hsa-miR-21-5p, hsa-miR-22-3p, hsa-miR-519d, 4023, 5465, 5467, 5468, 6256, 6257, 6258, hsa-miR-29a-3p, 1374, 5465, 5467, 5468, 6256, 6257, 6258, hsa-miR-370, 126129, 5465, 5467, 5468, 6256, 6257, 6258, 51129, 5465, 5467, 5468, 6256, 6257, 6258, 5465, 5467, 5468, 6256, 6257, 6258, 7350, 10062, 5465, 5468, 6256, 6257, 6258, hsa-miR-613, 5465, 5468, 6256, 6257, 6258, 6319, hsa-miR-1244, 10999, 5465, 5468, 6256, 6257, 6258, 2180, 5465, 5468, 6256, 6257, 6258, 2181, 5465, 5468, 6256, 6257, 6258, 2182, 5465, 5468, 6256, 6257, 6258, 23205, 5465, 5468, 6256, 6257, 6258, 23305, 5465, 5468, 6256, 6257, 6258, 376497, 5465, 5468, 6256, 6257, 6258, 51703, 5465, 5468, 6256, 6257, 6258, 5465, 5468, 6256, 6257, 6258, 79966, 5465, 5468, 6256, 6257, 6258, 81616, 4312, 5468, 6256, 6257, 6258, hsa-miR-222-3p, 5468, 6256, 6257, 6258, hsa-miR-130a-3p, hsa-miR-130b-3p, hsa-miR-20b-5p, hsa-miR-27b-3p, 2167, 5468, 6256, 6257, 6258, hsa-miR-369-5p, 10580, 5468, 6256, 6257, 6258, 1593, 5468, 6256, 6257, 6258, 2710, 5468, 6256, 6257, 6258, 2712, 5468, 6256, 6257, 6258, 364, 5468, 6256, 6257, 6258, 4973, 5468, 6256, 6257, 6258, 5105, 5468, 6256, 6257, 6258, 5106, 5468, 6256, 6257, 6258, 5346, 5468, 6256, 6257, 6258, 5468, 6256, 6257, 6258, 9370, 5468, 6256, 6257, 6258, 948 |
| Tuberculosis | 137.4 | 3458, 3459, hsa-miR-155-5p, 10332, 23365, 30835, 387, hsa-miR-122-5p, hsa-miR-155-5p, hsa-miR-185-5p, hsa-miR-31-5p, 10332, 30835, 387, 5894, hsa-miR-122-5p, hsa-miR-155-5p, hsa-miR-195-5p, 10332, 30835, 5600, 572, 5894, 7189, 8767, hsa-miR-122-5p, hsa-miR-155-5p, hsa-miR-195-5p, 10332, 1432, 30835, 5600, 572, 5894, 7189, 8767, hsa-miR-155-5p, hsa-miR-195-5p, hsa-miR-29b-3p, 1432, 4790, 5600, 572, 5894, 5970, 6772, 7189, 8767, hsa-miR-155-5p, hsa-miR-195-5p, hsa-miR-29b-3p, hsa-miR-34a-5p, hsa-miR-34b-5p, 1432, 4790, 5600, 572, 5894, 596, 5970, 6772, 7189, 8767, hsa-miR-125b-5p, hsa-miR-17-5p, hsa-miR-195-5p, hsa-miR-20a-5p, hsa-miR-21-5p, hsa-miR-29b-3p, hsa-miR-34a-5p, hsa-miR-34b-5p, 1432, 4790, 5600, 5894, 596, 5970, 6772, 7124, 7189, 8767, hsa-let-7a-5p, hsa-miR-125b-5p, hsa-miR-17-5p, hsa-miR-20a-5p, hsa-miR-21-5p, hsa-miR-34a-5p, 114609, 4615, 4790, 5894, 596, 5970, 6772, 7124, 7189, 8767, hsa-let-7a-5p, hsa-miR-125b-5p, hsa-miR-17-5p, hsa-miR-21-5p, hsa-miR-34a-5p, 114609, 4615, 4790, 5894, 596, 5970, 6772, 7124, 7189, 8767, hsa-let-7a-5p, hsa-miR-146a-5p, hsa-miR-17-5p, hsa-miR-21-5p, hsa-miR-34a-5p, 114609, 4615, 6772, 808, hsa-miR-146a-5p, hsa-miR-17-5p, hsa-miR-34a-5p, 4615, 6772, 808, hsa-miR-145-5p, hsa-miR-146a-5p, hsa-miR-17-5p, hsa-miR-34a-5p, 6772, 808, 815, 816, 817, 818, hsa-miR-145-5p, hsa-miR-146a-5p, hsa-miR-17-5p, 163688, 51806, 801, 805, 808, 810, 815, 816, 817, 818, hsa-miR-145-5p, 5289, 5868, 5869, 5878, 815, 816, 817, 818, 5289, 5868, 5869, 5878, 8411, hsa-miR-101-3p, 5868, 5869, 5878, 7879, 8411, 10312, 7879, 1509, 7879, 23545, 7879, 245972, 7879, 3916, 7879, 3920, 7879, 50617, 7879, 51606, 7879, 527, 7879, 533, 7879, 535, 7879, 537, 7879, 7879, 9114 |
| Dilated cardiomyopathy | 119.0 | 153, 2778, 112, 2778, hsa-miR-182-5p, hsa-miR-96-5p, 107, 108, 109, 111, 112, 113, 114, 115, 196883, 2778, 107, 108, 109, 111, 112, 113, 114, 115, 196883, 5566, 5567, 5568, 5613, 5566, 5567, 5568, 5613, 775, hsa-miR-133a, 5350, 5566, 5567, 5568, 5613, 10368, 5566, 5567, 5568, 5613, 10369, 5566, 5567, 5568, 5613, 27091, 5566, 5567, 5568, 5613, 27092, 5566, 5567, 5568, 5613, 5566, 5567, 5568, 55799, 5613, 5566, 5567, 5568, 5613, 59283, 5566, 5567, 5568, 5613, 59284, 5566, 5567, 5568, 5613, 59285, 5566, 5567, 5568, 5613, 6262, 5566, 5567, 5568, 5613, 776, 5566, 5567, 5568, 5613, 778, 5566, 5567, 5568, 5613, 779, 5566, 5567, 5568, 5613, 781, 5566, 5567, 5568, 5613, 782, 5566, 5567, 5568, 5613, 783, 5566, 5567, 5568, 5613, 784, 5566, 5567, 5568, 5613, 785, 5566, 5567, 5568, 5613, 786, 5566, 5567, 5568, 5613, 9254, 5566, 5567, 5568, 5613, 93589 |
| Tuberculosis | 109.1 | 3458, 3459, hsa-miR-155-5p, 10332, 23365, 30835, 387, hsa-miR-122-5p, hsa-miR-155-5p, hsa-miR-185-5p, hsa-miR-31-5p, 10332, 30835, 387, 5894, hsa-miR-122-5p, hsa-miR-155-5p, hsa-miR-195-5p, 10332, 30835, 5600, 572, 5894, 7189, 8767, hsa-miR-122-5p, hsa-miR-155-5p, hsa-miR-195-5p, 10332, 1432, 30835, 5600, 572, 5894, 7189, 8767, hsa-miR-155-5p, hsa-miR-195-5p, hsa-miR-29b-3p, 1432, 4790, 5600, 572, 5894, 5970, 6772, 7189, 8767, hsa-miR-155-5p, hsa-miR-195-5p, hsa-miR-29b-3p, hsa-miR-34a-5p, hsa-miR-34b-5p, 1432, 4790, 5600, 572, 5894, 596, 5970, 6772, 7189, 8767, hsa-miR-125b-5p, hsa-miR-17-5p, hsa-miR-195-5p, hsa-miR-20a-5p, hsa-miR-21-5p, hsa-miR-29b-3p, hsa-miR-34a-5p, hsa-miR-34b-5p, 1432, 4790, 5600, 5894, 596, 5970, 6772, 7124, 7189, 8767, hsa-let-7a-5p, hsa-miR-125b-5p, hsa-miR-17-5p, hsa-miR-20a-5p, hsa-miR-21-5p, hsa-miR-34a-5p, 114609, 4615, 4790, 5894, 596, 5970, 6772, 7124, 7189, 8767, hsa-let-7a-5p, hsa-miR-125b-5p, hsa-miR-17-5p, hsa-miR-21-5p, hsa-miR-34a-5p, 114609, 4615, 4790, 5894, 596, 5970, 6772, 7124, 7189, 8767, hsa-let-7a-5p, hsa-miR-146a-5p, hsa-miR-17-5p, hsa-miR-21-5p, hsa-miR-34a-5p, 114609, 4615, 6772, 808, hsa-miR-146a-5p, hsa-miR-17-5p, hsa-miR-34a-5p, 10333, 114609, 4615, 7096, 7097, 7099, 808, hsa-miR-146a-5p, 10333, 114609, 4615, 54106, 7096, 7097, 7099, 114609, 1594, 4615, 3440, 4615, 3441, 4615, 3442, 4615, 3443, 4615, 3444, 4615, 3445, 4615, 3446, 4615, 3448, 4615, 3449, 4615, 3452, 4615 |
| Toll-like receptor signaling pathway | 105.9 | 23643, 7099, 929, hsa-let-7i-5p, hsa-miR-146a-5p, 114609, 4615, 7097, 7099, hsa-miR-146a-5p, 114609, 4615, 5600, 5606, 5608, 7097, hsa-miR-146a-5p, hsa-miR-221-3p, 114609, 1432, 4615, 5600, 5603, 5606, 5608, 6300, hsa-miR-146a-5p, hsa-miR-221-3p, 114609, 1147, 1326, 1432, 4615, 5594, 5595, 5600, 5603, 5606, 6300, 7189, hsa-miR-146a-5p, hsa-miR-146b-5p, hsa-miR-155-5p, hsa-miR-221-3p, 114609, 1147, 1326, 148022, 4615, 5594, 5595, 7189, hsa-miR-146a-5p, hsa-miR-146b-5p, hsa-miR-155-5p, hsa-miR-221-3p, 114609, 1147, 1326, 148022, 3654, 4615, 5594, 5595, 7189, hsa-miR-146a-5p, hsa-miR-146b-5p, hsa-miR-155-5p, 1147, 1326, 148022, 29110, 3654, 5594, 5595, 7189, 9641, hsa-miR-146a-5p, hsa-miR-155-5p, 1147, 1326, 29110, 3654, 3665, 5594, 5595, 7189, 9641, hsa-miR-146a-5p, 1326, 29110, 3665, 5594, 5595, 9641, hsa-miR-146a-5p, hsa-miR-34a-5p, 29110, 3661, 3665, 9641, hsa-miR-146a-5p, hsa-miR-34a-5p, 3456, 3661, 3665, hsa-miR-145-5p, hsa-miR-146a-5p, hsa-miR-34a-5p, 3456, 3661, 3665, hsa-let-7b-5p, hsa-miR-145-5p, hsa-miR-26a-5p, hsa-miR-34a-5p, 3440, 3665, 3441, 3665, 3442, 3665, 3443, 3665, 3444, 3665, 3445, 3665, 3446, 3665, 3448, 3665, 3449, 3665, 3452, 3665 |
| Tuberculosis | 101.3 | 3458, 3459, hsa-miR-155-5p, 10332, 23365, 30835, 387, hsa-miR-122-5p, hsa-miR-155-5p, hsa-miR-185-5p, hsa-miR-31-5p, 10332, 30835, 387, 5894, hsa-miR-122-5p, hsa-miR-155-5p, hsa-miR-195-5p, 10332, 30835, 5600, 572, 5894, 7189, 8767, hsa-miR-122-5p, hsa-miR-155-5p, hsa-miR-195-5p, 10332, 1432, 30835, 5600, 572, 5894, 7189, 8767, hsa-miR-155-5p, hsa-miR-195-5p, hsa-miR-29b-3p, 1432, 4790, 5600, 572, 5894, 5970, 6772, 7189, 8767, hsa-miR-155-5p, hsa-miR-195-5p, hsa-miR-29b-3p, hsa-miR-34a-5p, hsa-miR-34b-5p, 4261, 4790, 5970, 6772, hsa-miR-155-5p, hsa-miR-34a-5p, hsa-miR-34b-5p, 1385, 4261, hsa-miR-103a-3p, hsa-miR-182-5p, hsa-miR-203a, hsa-miR-34b-5p, 1385, 4261, 4800, 4801, 4802, 5993, 5994, 8625, 1385, 3108, 4800, 4801, 4802, 5993, 5994, 8625, hsa-miR-638, 1385, 3115, 4800, 4801, 4802, 5993, 5994, 8625, hsa-miR-2861, 1385, 3109, 4800, 4801, 4802, 5993, 5994, 8625, 1385, 3111, 4800, 4801, 4802, 5993, 5994, 8625, 1385, 3112, 4800, 4801, 4802, 5993, 5994, 8625, 1385, 3113, 4800, 4801, 4802, 5993, 5994, 8625, 1385, 3117, 4800, 4801, 4802, 5993, 5994, 8625, 1385, 3118, 4800, 4801, 4802, 5993, 5994, 8625, 1385, 3119, 4800, 4801, 4802, 5993, 5994, 8625, 1385, 3122, 4800, 4801, 4802, 5993, 5994, 8625, 1385, 3123, 4800, 4801, 4802, 5993, 5994, 8625, 1385, 3127, 4800, 4801, 4802, 5993, 5994, 8625, 1385, 4800, 4801, 4802, 5993, 5994, 8625, 972 |
| Small cell lung cancer | 99.2 | 317, 54205, 842, hsa-miR-133a, 317, hsa-miR-21-5p, 5728, hsa-miR-106b-5p, hsa-miR-141-3p, hsa-miR-17-5p, hsa-miR-18a-5p, hsa-miR-19a-3p, hsa-miR-19b-3p, hsa-miR-20a-5p, hsa-miR-214-3p, hsa-miR-21-5p, hsa-miR-216a-5p, hsa-miR-217, hsa-miR-221-3p, hsa-miR-222-3p, hsa-miR-26a-5p, hsa-miR-29a-3p, hsa-miR-494, hsa-miR-519a-3p, hsa-miR-519d, hsa-miR-93-5p, 1869, 5728, hsa-miR-106a-5p, hsa-miR-106b-5p, hsa-miR-149-3p, hsa-miR-17-5p, hsa-miR-19a-3p, hsa-miR-203a, hsa-miR-20a-5p, hsa-miR-21-5p, hsa-miR-221-3p, hsa-miR-222-3p, hsa-miR-223-3p, hsa-miR-26a-5p, hsa-miR-29a-3p, hsa-miR-494, hsa-miR-519d, hsa-miR-93-5p, hsa-miR-98-5p, 1017, 1019, 1021, 1027, 1869, 4149, 4609, 5728, 595, 898, 9134, hsa-miR-106a-5p, hsa-miR-106b-5p, hsa-miR-149-3p, hsa-miR-17-5p, hsa-miR-195-5p, hsa-miR-19a-3p, hsa-miR-203a, hsa-miR-20a-5p, hsa-miR-218-5p, hsa-miR-221-3p, hsa-miR-222-3p, hsa-miR-223-3p, hsa-miR-24-3p, hsa-miR-26a-5p, hsa-miR-29a-3p, hsa-miR-34a-5p, hsa-miR-34c-5p, hsa-miR-494, hsa-miR-519d, hsa-miR-98-5p, 1017, 1019, 1021, 1027, 2335, 4149, 4609, 4790, 5728, 595, 5970, 898, 9134, hsa-miR-106b-5p, hsa-miR-124-3p, hsa-miR-149-3p, hsa-miR-15a-5p, hsa-miR-16-5p, hsa-miR-17-5p, hsa-miR-195-5p, hsa-miR-19a-3p, hsa-miR-203a, hsa-miR-20a-5p, hsa-miR-218-5p, hsa-miR-221-3p, hsa-miR-223-3p, hsa-miR-24-3p, hsa-miR-26a-5p, hsa-miR-29a-3p, hsa-miR-29b-3p, hsa-miR-29c-3p, hsa-miR-34a-5p, hsa-miR-34b-5p, hsa-miR-34c-5p, hsa-miR-449a, hsa-miR-494, hsa-miR-503-5p, hsa-miR-519d, hsa-miR-98-5p, 1017, 1027, 2335, 4790, 5728, 5970, 898, 9134, hsa-miR-124-3p, hsa-miR-126-3p, hsa-miR-149-3p, hsa-miR-15a-5p, hsa-miR-16-5p, hsa-miR-218-5p, hsa-miR-221-3p, hsa-miR-223-3p, hsa-miR-26a-5p, hsa-miR-29a-3p, hsa-miR-29b-3p, hsa-miR-29c-3p, hsa-miR-34a-5p, hsa-miR-34b-5p, hsa-miR-34c-5p, hsa-miR-449a, hsa-miR-503-5p, hsa-miR-519d, 2335, 3655, 3673, 3674, 3675, 3685, 3688, 4790, 5728, 5970, hsa-miR-124-3p, hsa-miR-126-3p, hsa-miR-149-3p, hsa-miR-15a-5p, hsa-miR-16-5p, hsa-miR-218-5p, hsa-miR-221-3p, hsa-miR-223-3p, hsa-miR-29a-3p, hsa-miR-29b-3p, hsa-miR-29c-3p, hsa-miR-519d, 10000, 207, 208, 3655, 3673, 3674, 3675, 3685, 3688, 4790, 5728, 5970, hsa-miR-124-3p, hsa-miR-126-3p, hsa-miR-149-3p, hsa-miR-15a-5p, hsa-miR-16-5p, hsa-miR-218-5p, hsa-miR-221-3p, hsa-miR-223-3p, hsa-miR-29a-3p, hsa-miR-29b-3p, hsa-miR-29c-3p, hsa-miR-519d, 10000, 1147, 207, 208, 4790, 5970, hsa-miR-124-3p, hsa-miR-15a-5p, hsa-miR-16-5p, hsa-miR-218-5p, hsa-miR-223-3p, 10000, 1147, 207, 208, 3551, 4790, 5970, hsa-miR-124-3p, hsa-miR-218-5p, 10000, 1147, 207, 208, 3551, 4790, 5970, 8517, hsa-miR-124-3p, 1147, 3551, 4790, 4792, 5970, 8517, hsa-miR-124-3p, 4790, 4792, 5970, hsa-miR-146a-5p, hsa-miR-146b-5p, 4790, 5970, 7189, hsa-miR-146a-5p, hsa-miR-146b-5p, 331, 4790, 5970, hsa-miR-34a-3p, 329, 4790, 5970, hsa-miR-204-5p, 112401, 4790, 5970, 330, 4790, 5970, 4790, 4843, 5970, 4790, 5970, 7185, 4790, 5970, 7186, 4790, 5970, 7187, 4790, 5970, 7188, 4790, 5970, 79444, 4790, 5970, 9618 |
| Bacterial invasion of epithelial cells | 99.1 | 26084, 391, 391, 9844, 1398, 1399, 1793, 9844, 1398, 1399, 1793, 2549, 1398, 1793, 2549, 5296, 10163, 1793, 2549, 5296, 8936, hsa-miR-122-5p, 10163, 2549, 387, 5296, 8936, hsa-miR-122-5p, hsa-miR-185-5p, hsa-miR-31-5p, 10163, 2549, 387, 5296, 8936, 998, hsa-miR-1, hsa-miR-185-5p, hsa-miR-31-5p, 10092, 10093, 10094, 10095, 10109, 10163, 10552, 387, 81873, 8936, 998, hsa-miR-1, hsa-miR-31-5p, 10092, 10093, 10094, 10095, 10109, 10552, 387, 5829, 79658, 81873, hsa-miR-1, hsa-miR-31-5p, 2335, 5829, 79658, hsa-miR-1, hsa-miR-31-5p, 2335, 3678, 5829, 79658, hsa-miR-31-5p, 2335, 3678, 3688, 5829, 3611, 3678, 3688, 5829, 3611, 5829, 9564, 5747, 6714, 9564, 5747, 6714, hsa-miR-138-5p, hsa-miR-193a-3p, 2017, 3059, 6714, 10059, 2017, 3059, 1759, 2017, 3059, 1785, 2017, 3059, 2017, 26052, 3059 |
| Amphetamine addiction | 98.3 | 163688, 51806, 801, 805, 808, 810, 818, hsa-miR-219-5p, 163688, 51806, 801, 805, 808, 810, 814, 815, 816, 817, 818, 1385, 5499, 5500, 5501, 814, 815, 816, 817, 818, hsa-miR-103a-3p, hsa-miR-182-5p, hsa-miR-203a, hsa-miR-34b-5p, 10488, 1385, 1386, 1388, 148327, 468, 5499, 5500, 5501, 64764, 814, 815, 816, 817, 818, 84699, 90993, 9586, 10488, 1385, 1386, 1388, 148327, 23411, 2354, 3065, 468, 5499, 5500, 5501, 64764, 815, 816, 817, 818, 84699, 90993, 9586, 23411, 2354, 3065, 5499, 5500, 5501, 815, 816, 817, 818, hsa-miR-34a-5p, 23411, 2354, 3065, 5499, 5500, 5501, hsa-miR-181b-5p, hsa-miR-34a-5p, 5499, 5500, 5501, hsa-miR-125b-5p, hsa-miR-181b-5p, 5566, 5567, 5568, 5613, hsa-miR-125b-5p, hsa-miR-181b-5p, 2903, 5566, 5567, 5568, 5613, hsa-miR-125b-5p, 5566, 5567, 5568, 5613, 775, hsa-miR-133a, 111, 5566, 5567, 5568, 5613, 116443, 5566, 5567, 5568, 5613, 116444, 5566, 5567, 5568, 5613, 2890, 5566, 5567, 5568, 5613, 2892, 5566, 5567, 5568, 5613, 2893, 5566, 5567, 5568, 5613, 2902, 5566, 5567, 5568, 5613, 2904, 5566, 5567, 5568, 5613, 2905, 5566, 5567, 5568, 5613, 2906, 5566, 5567, 5568, 5613, 5566, 5567, 5568, 5613, 776 |

**Table S3. Re-analysis of the meta-pathway.** The first two paths have been considered to compose the network in Supplementary Figure 3.

| **Max Score** | **Involved Genes and miRNAs** |
| --- | --- |
| 211.20 | 26084, 391,391, 9844,1398, 1399, 1793, 9844,1398, 1399, 2549, 5296,5296, 998, hsa-miR-185-5p,10332, 23365, 30835, 387, hsa-miR-122-5p, hsa-miR-155-5p, hsa-miR-185-5p, hsa-miR-31-5p,387, 5894, hsa-miR-122-5p, hsa-miR-155-5p, hsa-miR-195-5p, hsa-miR-31-5p,5894, hsa-miR-122-5p, hsa-miR-124-3p, hsa-miR-155-5p, hsa-miR-195-5p, hsa-miR-31-5p,4790, 4792, 5894, 5970, 7189, hsa-miR-122-5p, hsa-miR-124-3p, hsa-miR-146a-5p, hsa-miR-146b-5p, hsa-miR-155-5p, hsa-miR-195-5p,1147, 4790, 4792, 5970, 7189, hsa-miR-122-5p, hsa-miR-124-3p, hsa-miR-145-5p, hsa-miR-146a-5p, hsa-miR-146b-5p, hsa-miR-155-5p, hsa-miR-195-5p, hsa-miR-221-3p, hsa-miR-26a-5p, hsa-miR-34a-5p,1147, 1387, 2033, 3091, 405, 4790, 4792, 5970, hsa-miR-122-5p, hsa-miR-124-3p, hsa-miR-145-5p, hsa-miR-146a-5p, hsa-miR-17-5p, hsa-miR-195-5p, hsa-miR-203a, hsa-miR-20a-5p, hsa-miR-21-5p, hsa-miR-221-3p, hsa-miR-26a-5p, hsa-miR-29b-3p, hsa-miR-34a-5p, hsa-miR-34b-5p,1387, 2033, 3091, 405, 5499, hsa-miR-145-5p, hsa-miR-146a-5p, hsa-miR-17-5p, hsa-miR-182-5p, hsa-miR-195-5p, hsa-miR-203a, hsa-miR-20a-5p, hsa-miR-21-5p, hsa-miR-29b-3p, hsa-miR-34a-5p, hsa-miR-34b-5p,5499, 5566, 5567, 5568, 5613, hsa-miR-17-5p, hsa-miR-182-5p, hsa-miR-195-5p, hsa-miR-20a-5p, hsa-miR-21-5p, hsa-miR-29b-3p, hsa-miR-34a-5p, hsa-miR-34b-5p,5499, 5566, 5567, 5568, 5613, hsa-miR-125b-5p, hsa-miR-17-5p, hsa-miR-195-5p, hsa-miR-20a-5p, hsa-miR-21-5p, hsa-miR-29b-3p, hsa-miR-34a-5p, hsa-miR-34b-5p,5566, 5567, 5568, 5613, hsa-miR-133a, hsa-miR-21-5p,5566, 5567, 5568, 5613, 775, hsa-miR-133a,10368, 5566, 5567, 5568, 5613,10369, 5566, 5567, 5568, 5613,116443, 5566, 5567, 5568, 5613,116444, 5566, 5567, 5568, 5613,27091, 5566, 5567, 5568, 5613,27092, 5566, 5567, 5568, 5613,2890, 5566, 5567, 5568, 5613,2892, 5566, 5567, 5568, 5613,2893, 5566, 5567, 5568, 5613,2902, 5566, 5567, 5568, 5613,2904, 5566, 5567, 5568, 5613,2905, 5566, 5567, 5568, 5613,2906, 5566, 5567, 5568, 5613,5350, 5566, 5567, 5568, 5613,5566, 5567, 5568, 55799, 5613,5566, 5567, 5568, 5613, 59283,5566, 5567, 5568, 5613, 59284,5566, 5567, 5568, 5613, 59285,5566, 5567, 5568, 5613, 6262,5566, 5567, 5568, 5613, 776,5566, 5567, 5568, 5613, 778,5566, 5567, 5568, 5613, 779,5566, 5567, 5568, 5613, 781,5566, 5567, 5568, 5613, 782,5566, 5567, 5568, 5613, 783,5566, 5567, 5568, 5613, 784,5566, 5567, 5568, 5613, 785,5566, 5567, 5568, 5613, 786,5566, 5567, 5568, 5613, 9254,5566, 5567, 5568, 5613, 93589 |
| 198.02 | 26084, 391,391, 9844,1398, 1399, 1793, 9844,1398, 1399, 2549, 5296,5296, 998, hsa-miR-185-5p,10332, 23365, 30835, 387, hsa-miR-122-5p, hsa-miR-155-5p, hsa-miR-185-5p, hsa-miR-31-5p,387, 5894, hsa-miR-122-5p, hsa-miR-155-5p, hsa-miR-195-5p, hsa-miR-31-5p,5894, hsa-miR-122-5p, hsa-miR-124-3p, hsa-miR-155-5p, hsa-miR-195-5p, hsa-miR-31-5p,4790, 4792, 5894, 5970, 7189, hsa-miR-122-5p, hsa-miR-124-3p, hsa-miR-146a-5p, hsa-miR-146b-5p, hsa-miR-155-5p, hsa-miR-195-5p,1147, 4790, 4792, 5970, 7189, hsa-miR-122-5p, hsa-miR-124-3p, hsa-miR-145-5p, hsa-miR-146a-5p, hsa-miR-146b-5p, hsa-miR-155-5p, hsa-miR-195-5p, hsa-miR-221-3p, hsa-miR-26a-5p, hsa-miR-34a-5p,1147, 1387, 2033, 3091, 405, 4790, 4792, 5970, hsa-miR-122-5p, hsa-miR-124-3p, hsa-miR-145-5p, hsa-miR-146a-5p, hsa-miR-17-5p, hsa-miR-195-5p, hsa-miR-203a, hsa-miR-20a-5p, hsa-miR-21-5p, hsa-miR-221-3p, hsa-miR-26a-5p, hsa-miR-29b-3p, hsa-miR-34a-5p, hsa-miR-34b-5p,10000, 1147, 1387, 2033, 207, 208, 3091, 405, 4790, 4792, 5970, hsa-miR-122-5p, hsa-miR-124-3p, hsa-miR-145-5p, hsa-miR-17-5p, hsa-miR-195-5p, hsa-miR-203a, hsa-miR-20a-5p, hsa-miR-21-5p, hsa-miR-221-3p, hsa-miR-26a-5p, hsa-miR-29b-3p, hsa-miR-34a-5p, hsa-miR-34b-5p,10000, 1147, 1387, 2033, 207, 208, 3091, 405, 4790, 4792, 5728, 5970, hsa-miR-106b-5p, hsa-miR-122-5p, hsa-miR-124-3p, hsa-miR-17-5p, hsa-miR-195-5p, hsa-miR-19a-3p, hsa-miR-203a, hsa-miR-20a-5p, hsa-miR-21-5p, hsa-miR-221-3p, hsa-miR-222-3p, hsa-miR-26a-5p, hsa-miR-29a-3p, hsa-miR-29b-3p, hsa-miR-34a-5p, hsa-miR-34b-5p, hsa-miR-494, hsa-miR-93-5p,10000, 1387, 2033, 207, 208, 3091, 405, 5728, hsa-miR-106b-5p, hsa-miR-122-5p, hsa-miR-17-5p, hsa-miR-19a-3p, hsa-miR-20a-5p, hsa-miR-21-5p, hsa-miR-221-3p, hsa-miR-222-3p, hsa-miR-26a-5p, hsa-miR-29a-3p, hsa-miR-494, hsa-miR-519d, hsa-miR-93-5p,1387, 2033, 3091, 405, hsa-miR-1244, hsa-miR-21-5p, hsa-miR-222-3p, hsa-miR-22-3p, hsa-miR-29a-3p, hsa-miR-519d,1387, 2033, 3091, 405, 4846, hsa-miR-1244,1387, 2033, 3091, 405, 5163, hsa-miR-375,1387, 2023, 2033, 3091, 405,1387, 2026, 2033, 3091, 405,1387, 2027, 2033, 3091, 405,1387, 2033, 2056, 3091, 405,1387, 2033, 284, 3091, 405,1387, 2033, 285, 3091, 405,1387, 2033, 3091, 3098, 405,1387, 2033, 3091, 3101, 405,1387, 2033, 3091, 3939, 405,1387, 2033, 3091, 405, 4055,1387, 2033, 3091, 405, 4878,1387, 2033, 3091, 405, 51378,1387, 2033, 3091, 405, 5207,1387, 2033, 3091, 405, 5208,1387, 2033, 3091, 405, 5209,1387, 2033, 3091, 405, 5210,1387, 2033, 3091, 405, 5211,1387, 2033, 3091, 405, 5228,1387, 2033, 3091, 405, 5230,1387, 2033, 3091, 405, 6513,1387, 2033, 3091, 405, 7010,1387, 2033, 3091, 405, 7018,1387, 2033, 3091, 405, 7076,1387, 2033, 3091, 405, 7423,1387, 2033, 3091, 405, 7424,1387, 2033, 3091, 405, 80201 |
| 151.69 | 26084, 391,391, 9844,1398, 1399, 1793, 9844,1398, 1399, 2549, 5296,5296, 998, hsa-miR-185-5p,10332, 23365, 30835, 387, hsa-miR-122-5p, hsa-miR-155-5p, hsa-miR-185-5p, hsa-miR-31-5p,387, 5894, hsa-miR-122-5p, hsa-miR-155-5p, hsa-miR-195-5p, hsa-miR-31-5p,5894, hsa-miR-122-5p, hsa-miR-124-3p, hsa-miR-155-5p, hsa-miR-195-5p, hsa-miR-31-5p,4790, 4792, 5894, 5970, 7189, hsa-miR-122-5p, hsa-miR-124-3p, hsa-miR-146a-5p, hsa-miR-146b-5p, hsa-miR-155-5p, hsa-miR-195-5p,1147, 4790, 4792, 5970, 7189, hsa-miR-122-5p, hsa-miR-124-3p, hsa-miR-145-5p, hsa-miR-146a-5p, hsa-miR-146b-5p, hsa-miR-155-5p, hsa-miR-195-5p, hsa-miR-221-3p, hsa-miR-26a-5p, hsa-miR-34a-5p,1147, 1387, 2033, 3091, 405, 4790, 4792, 5970, hsa-miR-122-5p, hsa-miR-124-3p, hsa-miR-145-5p, hsa-miR-146a-5p, hsa-miR-17-5p, hsa-miR-195-5p, hsa-miR-203a, hsa-miR-20a-5p, hsa-miR-21-5p, hsa-miR-221-3p, hsa-miR-26a-5p, hsa-miR-29b-3p, hsa-miR-34a-5p, hsa-miR-34b-5p,10000, 1147, 1387, 2033, 207, 208, 3091, 405, 4790, 4792, 5970, hsa-miR-122-5p, hsa-miR-124-3p, hsa-miR-145-5p, hsa-miR-17-5p, hsa-miR-195-5p, hsa-miR-203a, hsa-miR-20a-5p, hsa-miR-21-5p, hsa-miR-221-3p, hsa-miR-26a-5p, hsa-miR-29b-3p, hsa-miR-34a-5p, hsa-miR-34b-5p,10000, 1147, 1387, 2033, 207, 208, 3091, 405, 4790, 4792, 5728, 5970, hsa-miR-106b-5p, hsa-miR-122-5p, hsa-miR-124-3p, hsa-miR-17-5p, hsa-miR-195-5p, hsa-miR-19a-3p, hsa-miR-203a, hsa-miR-20a-5p, hsa-miR-21-5p, hsa-miR-221-3p, hsa-miR-222-3p, hsa-miR-26a-5p, hsa-miR-29a-3p, hsa-miR-29b-3p, hsa-miR-34a-5p, hsa-miR-34b-5p, hsa-miR-494, hsa-miR-93-5p,10000, 1387, 2033, 207, 208, 3091, 405, 5728, hsa-miR-106b-5p, hsa-miR-122-5p, hsa-miR-17-5p, hsa-miR-19a-3p, hsa-miR-20a-5p, hsa-miR-21-5p, hsa-miR-221-3p, hsa-miR-222-3p, hsa-miR-26a-5p, hsa-miR-29a-3p, hsa-miR-494, hsa-miR-519d, hsa-miR-93-5p,1387, 2033, 3091, 405, hsa-miR-1244, hsa-miR-21-5p, hsa-miR-222-3p, hsa-miR-22-3p, hsa-miR-29a-3p, hsa-miR-519d,5465, 6256, 6257, 6258, hsa-miR-1244, hsa-miR-21-5p, hsa-miR-222-3p, hsa-miR-22-3p, hsa-miR-29a-3p, hsa-miR-519d,5465, 5468, 6256, 6257, 6258, hsa-miR-1244, hsa-miR-222-3p, hsa-miR-29a-3p,4023, 5465, 5467, 5468, 6256, 6257, 6258, hsa-miR-29a-3p,1374, 5465, 5467, 5468, 6256, 6257, 6258, hsa-miR-370,126129, 5465, 5467, 5468, 6256, 6257, 6258,51129, 5465, 5467, 5468, 6256, 6257, 6258,5465, 5467, 5468, 6256, 6257, 6258, 7350,10062, 5465, 5468, 6256, 6257, 6258, hsa-miR-613,10999, 5465, 5468, 6256, 6257, 6258,2180, 5465, 5468, 6256, 6257, 6258,2181, 5465, 5468, 6256, 6257, 6258,2182, 5465, 5468, 6256, 6257, 6258,23205, 5465, 5468, 6256, 6257, 6258,23305, 5465, 5468, 6256, 6257, 6258,376497, 5465, 5468, 6256, 6257, 6258,51703, 5465, 5468, 6256, 6257, 6258,5465, 5468, 6256, 6257, 6258, 79966,5465, 5468, 6256, 6257, 6258, 81616 |
| 144.38 | 26084, 391,391, 9844,1398, 1399, 1793, 9844,1398, 1399, 2549, 5296,5296, 998, hsa-miR-185-5p,10332, 23365, 30835, 387, hsa-miR-122-5p, hsa-miR-155-5p, hsa-miR-185-5p, hsa-miR-31-5p,387, 5894, hsa-miR-122-5p, hsa-miR-155-5p, hsa-miR-195-5p, hsa-miR-31-5p,5894, hsa-miR-122-5p, hsa-miR-124-3p, hsa-miR-155-5p, hsa-miR-195-5p, hsa-miR-31-5p,4790, 4792, 5894, 5970, 7189, hsa-miR-122-5p, hsa-miR-124-3p, hsa-miR-146a-5p, hsa-miR-146b-5p, hsa-miR-155-5p, hsa-miR-195-5p,1147, 4790, 4792, 5970, 7189, hsa-miR-122-5p, hsa-miR-124-3p, hsa-miR-145-5p, hsa-miR-146a-5p, hsa-miR-146b-5p, hsa-miR-155-5p, hsa-miR-195-5p, hsa-miR-221-3p, hsa-miR-26a-5p, hsa-miR-34a-5p,1147, 1387, 2033, 3091, 405, 4790, 4792, 5970, hsa-miR-122-5p, hsa-miR-124-3p, hsa-miR-145-5p, hsa-miR-146a-5p, hsa-miR-17-5p, hsa-miR-195-5p, hsa-miR-203a, hsa-miR-20a-5p, hsa-miR-21-5p, hsa-miR-221-3p, hsa-miR-26a-5p, hsa-miR-29b-3p, hsa-miR-34a-5p, hsa-miR-34b-5p,1387, 2033, 3091, 405, 5499, hsa-miR-145-5p, hsa-miR-146a-5p, hsa-miR-17-5p, hsa-miR-182-5p, hsa-miR-195-5p, hsa-miR-203a, hsa-miR-20a-5p, hsa-miR-21-5p, hsa-miR-29b-3p, hsa-miR-34a-5p, hsa-miR-34b-5p,1387, 2033, 3091, 405, 5499, 815, 816, 817, 818, hsa-miR-145-5p, hsa-miR-146a-5p, hsa-miR-182-5p, hsa-miR-203a, hsa-miR-34a-5p, hsa-miR-34b-5p,1385, 5499, 5500, 5501, 814, 815, 816, 817, 818, hsa-miR-145-5p, hsa-miR-146a-5p, hsa-miR-182-5p, hsa-miR-203a, hsa-miR-34a-5p, hsa-miR-34b-5p,10488, 1385, 1386, 1388, 148327, 468, 5499, 5500, 5501, 64764, 814, 815, 816, 817, 818, 84699, 90993, 9586, hsa-miR-34a-5p,163688, 51806, 801, 805, 808, 810, 814, 815, 816, 817, 818,5289, 5868, 5869, 5878, 815, 816, 817, 818,5289, 5868, 5869, 5878, 8411, hsa-miR-101-3p,5868, 5869, 5878, 7879, 8411,10312, 7879,1509, 7879,23545, 7879,245972, 7879,3916, 7879,3920, 7879,50617, 7879,51606, 7879,527, 7879,533, 7879,535, 7879,537, 7879,7879, 9114 |
| 142.76 | 26084, 391,391, 9844,1398, 1399, 1793, 9844,1398, 1399, 2549, 5296,5296, 998, hsa-miR-185-5p,10332, 23365, 30835, 387, hsa-miR-122-5p, hsa-miR-155-5p, hsa-miR-185-5p, hsa-miR-31-5p,387, 5894, hsa-miR-122-5p, hsa-miR-155-5p, hsa-miR-195-5p, hsa-miR-31-5p,5894, hsa-miR-122-5p, hsa-miR-124-3p, hsa-miR-155-5p, hsa-miR-195-5p, hsa-miR-31-5p,4790, 4792, 5894, 5970, 7189, hsa-miR-122-5p, hsa-miR-124-3p, hsa-miR-146a-5p, hsa-miR-146b-5p, hsa-miR-155-5p, hsa-miR-195-5p,1147, 4790, 4792, 5970, 7189, hsa-miR-122-5p, hsa-miR-124-3p, hsa-miR-145-5p, hsa-miR-146a-5p, hsa-miR-146b-5p, hsa-miR-155-5p, hsa-miR-195-5p, hsa-miR-221-3p, hsa-miR-26a-5p, hsa-miR-34a-5p,1147, 1387, 2033, 3091, 405, 4790, 4792, 5970, hsa-miR-122-5p, hsa-miR-124-3p, hsa-miR-145-5p, hsa-miR-146a-5p, hsa-miR-17-5p, hsa-miR-195-5p, hsa-miR-203a, hsa-miR-20a-5p, hsa-miR-21-5p, hsa-miR-221-3p, hsa-miR-26a-5p, hsa-miR-29b-3p, hsa-miR-34a-5p, hsa-miR-34b-5p,10000, 1147, 1387, 2033, 207, 208, 3091, 405, 4790, 4792, 5970, hsa-miR-122-5p, hsa-miR-124-3p, hsa-miR-145-5p, hsa-miR-17-5p, hsa-miR-195-5p, hsa-miR-203a, hsa-miR-20a-5p, hsa-miR-21-5p, hsa-miR-221-3p, hsa-miR-26a-5p, hsa-miR-29b-3p, hsa-miR-34a-5p, hsa-miR-34b-5p,10000, 1147, 1387, 2033, 207, 208, 3091, 405, 4790, 4792, 5728, 5970, hsa-miR-106b-5p, hsa-miR-122-5p, hsa-miR-124-3p, hsa-miR-17-5p, hsa-miR-195-5p, hsa-miR-19a-3p, hsa-miR-203a, hsa-miR-20a-5p, hsa-miR-21-5p, hsa-miR-221-3p, hsa-miR-222-3p, hsa-miR-26a-5p, hsa-miR-29a-3p, hsa-miR-29b-3p, hsa-miR-34a-5p, hsa-miR-34b-5p, hsa-miR-494, hsa-miR-93-5p,10000, 1387, 2033, 207, 208, 3091, 405, 5728, hsa-miR-106b-5p, hsa-miR-122-5p, hsa-miR-17-5p, hsa-miR-19a-3p, hsa-miR-20a-5p, hsa-miR-21-5p, hsa-miR-221-3p, hsa-miR-222-3p, hsa-miR-26a-5p, hsa-miR-29a-3p, hsa-miR-494, hsa-miR-519d, hsa-miR-93-5p,1387, 2033, 3091, 405, hsa-miR-1244, hsa-miR-21-5p, hsa-miR-222-3p, hsa-miR-22-3p, hsa-miR-29a-3p, hsa-miR-519d,5465, 6256, 6257, 6258, hsa-miR-1244, hsa-miR-21-5p, hsa-miR-222-3p, hsa-miR-22-3p, hsa-miR-29a-3p, hsa-miR-519d,5465, 5468, 6256, 6257, 6258, hsa-miR-1244, hsa-miR-222-3p, hsa-miR-29a-3p,4312, 5468, 6256, 6257, 6258, hsa-miR-222-3p,5468, 6256, 6257, 6258, hsa-miR-130a-3p, hsa-miR-130b-3p, hsa-miR-20b-5p, hsa-miR-27b-3p,2167, 5468, 6256, 6257, 6258, hsa-miR-369-5p,10580, 5468, 6256, 6257, 6258,1593, 5468, 6256, 6257, 6258,2710, 5468, 6256, 6257, 6258,2712, 5468, 6256, 6257, 6258,364, 5468, 6256, 6257, 6258,4973, 5468, 6256, 6257, 6258,5105, 5468, 6256, 6257, 6258,5106, 5468, 6256, 6257, 6258,5346, 5468, 6256, 6257, 6258,5468, 6256, 6257, 6258, 9370,5468, 6256, 6257, 6258, 948 |
| 138.16 | 26084, 391,391, 9844,1398, 1399, 1793, 9844,1398, 1399, 2549, 5296,5296, 998, hsa-miR-185-5p,10332, 23365, 30835, 387, hsa-miR-122-5p, hsa-miR-155-5p, hsa-miR-185-5p, hsa-miR-31-5p,387, 5894, hsa-miR-122-5p, hsa-miR-155-5p, hsa-miR-195-5p, hsa-miR-31-5p,5894, hsa-miR-122-5p, hsa-miR-124-3p, hsa-miR-155-5p, hsa-miR-195-5p, hsa-miR-31-5p,4790, 4792, 5894, 5970, 7189, hsa-miR-122-5p, hsa-miR-124-3p, hsa-miR-146a-5p, hsa-miR-146b-5p, hsa-miR-155-5p, hsa-miR-195-5p,1147, 4790, 4792, 5970, 7189, hsa-miR-122-5p, hsa-miR-124-3p, hsa-miR-145-5p, hsa-miR-146a-5p, hsa-miR-146b-5p, hsa-miR-155-5p, hsa-miR-195-5p, hsa-miR-221-3p, hsa-miR-26a-5p, hsa-miR-34a-5p,1147, 1387, 2033, 3091, 405, 4790, 4792, 5970, hsa-miR-122-5p, hsa-miR-124-3p, hsa-miR-145-5p, hsa-miR-146a-5p, hsa-miR-17-5p, hsa-miR-195-5p, hsa-miR-203a, hsa-miR-20a-5p, hsa-miR-21-5p, hsa-miR-221-3p, hsa-miR-26a-5p, hsa-miR-29b-3p, hsa-miR-34a-5p, hsa-miR-34b-5p,1387, 2033, 3091, 405, 5499, hsa-miR-145-5p, hsa-miR-146a-5p, hsa-miR-17-5p, hsa-miR-182-5p, hsa-miR-195-5p, hsa-miR-203a, hsa-miR-20a-5p, hsa-miR-21-5p, hsa-miR-29b-3p, hsa-miR-34a-5p, hsa-miR-34b-5p,1387, 2033, 3091, 405, 5499, 815, 816, 817, 818, hsa-miR-145-5p, hsa-miR-146a-5p, hsa-miR-182-5p, hsa-miR-203a, hsa-miR-34a-5p, hsa-miR-34b-5p,1385, 5499, 5500, 5501, 814, 815, 816, 817, 818, hsa-miR-145-5p, hsa-miR-146a-5p, hsa-miR-182-5p, hsa-miR-203a, hsa-miR-34a-5p, hsa-miR-34b-5p,1385, 4261, 5499, 5500, 5501, 814, 815, 816, 817, 818, hsa-miR-145-5p, hsa-miR-146a-5p, hsa-miR-182-5p, hsa-miR-203a, hsa-miR-34b-5p,1385, 4261, 5499, 5500, 5501, 814, 815, 816, 817, 818, hsa-miR-103a-3p, hsa-miR-182-5p, hsa-miR-203a, hsa-miR-34b-5p,1385, 4261, 4800, 4801, 4802, 5993, 5994, 8625,1385, 3115, 4800, 4801, 4802, 5993, 5994, 8625, hsa-miR-2861,1385, 3108, 4800, 4801, 4802, 5993, 5994, 8625, hsa-miR-638,1385, 3109, 4800, 4801, 4802, 5993, 5994, 8625,1385, 3111, 4800, 4801, 4802, 5993, 5994, 8625,1385, 3112, 4800, 4801, 4802, 5993, 5994, 8625,1385, 3113, 4800, 4801, 4802, 5993, 5994, 8625,1385, 3117, 4800, 4801, 4802, 5993, 5994, 8625,1385, 3118, 4800, 4801, 4802, 5993, 5994, 8625,1385, 3119, 4800, 4801, 4802, 5993, 5994, 8625,1385, 3122, 4800, 4801, 4802, 5993, 5994, 8625,1385, 3123, 4800, 4801, 4802, 5993, 5994, 8625,1385, 3127, 4800, 4801, 4802, 5993, 5994, 8625,1385, 4800, 4801, 4802, 5993, 5994, 8625, 972 |
| 101.31 | 26084, 391,391, 9844,1398, 1399, 1793, 9844,1398, 1399, 2549, 5296,5296, 998, hsa-miR-185-5p,10332, 23365, 30835, 387, hsa-miR-122-5p, hsa-miR-155-5p, hsa-miR-185-5p, hsa-miR-31-5p,387, 5894, hsa-miR-122-5p, hsa-miR-155-5p, hsa-miR-195-5p, hsa-miR-31-5p,5894, hsa-miR-122-5p, hsa-miR-124-3p, hsa-miR-155-5p, hsa-miR-195-5p, hsa-miR-31-5p,4790, 4792, 5894, 5970, 7189, hsa-miR-122-5p, hsa-miR-124-3p, hsa-miR-146a-5p, hsa-miR-146b-5p, hsa-miR-155-5p, hsa-miR-195-5p,1147, 4790, 4792, 5970, 7189, hsa-miR-122-5p, hsa-miR-124-3p, hsa-miR-145-5p, hsa-miR-146a-5p, hsa-miR-146b-5p, hsa-miR-155-5p, hsa-miR-195-5p, hsa-miR-221-3p, hsa-miR-26a-5p, hsa-miR-34a-5p,1147, 3654, 4615, 4790, 5970, 7189, hsa-miR-145-5p, hsa-miR-146a-5p, hsa-miR-146b-5p, hsa-miR-155-5p, hsa-miR-221-3p, hsa-miR-26a-5p, hsa-miR-34a-5p,1147, 3654, 3665, 4615, 7189, hsa-miR-145-5p, hsa-miR-155-5p, hsa-miR-26a-5p, hsa-miR-34a-5p,3446, 3665, 4615,3448, 3665, 4615,3449, 3665, 4615,3452, 3665, 4615,3440, 3665, 4615,3441, 3665, 4615,3442, 3665, 4615,3443, 3665, 4615,3444, 3665, 4615,3445, 3665, 4615 |
| 101.31 | 26084, 391,391, 9844,1398, 1399, 1793, 9844,1398, 1399, 2549, 5296,5296, 998, hsa-miR-185-5p,10332, 23365, 30835, 387, hsa-miR-122-5p, hsa-miR-155-5p, hsa-miR-185-5p, hsa-miR-31-5p,387, 5894, hsa-miR-122-5p, hsa-miR-155-5p, hsa-miR-195-5p, hsa-miR-31-5p,5894, hsa-miR-122-5p, hsa-miR-124-3p, hsa-miR-155-5p, hsa-miR-195-5p, hsa-miR-31-5p,4790, 4792, 5894, 5970, 7189, hsa-miR-122-5p, hsa-miR-124-3p, hsa-miR-146a-5p, hsa-miR-146b-5p, hsa-miR-155-5p, hsa-miR-195-5p,4790, 4792, 5894, 5970, 7189, 8767, hsa-miR-122-5p, hsa-miR-124-3p, hsa-miR-146a-5p, hsa-miR-146b-5p,1432, 4790, 5600, 5970, 7189, 8767, hsa-miR-122-5p,1432, 4790, 5600, 5970, 7124,112401, 4790, 5970,329, 4790, 5970, hsa-miR-204-5p,331, 4790, 5970, hsa-miR-34a-3p,4790, 5970, 7186,4790, 5970, 7187,4790, 5970, 7188,4790, 5970, 79444,4790, 5970, 9618,330, 4790, 5970,4790, 5970, 7185 |
| 87.50 | 26084, 391,391, 9844,1398, 1399, 1793, 9844,1398, 1399, 2549, 5296,5296, 998, hsa-miR-185-5p,10332, 23365, 30835, 387, hsa-miR-122-5p, hsa-miR-155-5p, hsa-miR-185-5p, hsa-miR-31-5p,387, 5894, hsa-miR-122-5p, hsa-miR-155-5p, hsa-miR-195-5p, hsa-miR-31-5p,5894, hsa-miR-122-5p, hsa-miR-124-3p, hsa-miR-155-5p, hsa-miR-195-5p, hsa-miR-31-5p,4790, 4792, 5894, 5970, 7189, hsa-miR-122-5p, hsa-miR-124-3p, hsa-miR-146a-5p, hsa-miR-146b-5p, hsa-miR-155-5p, hsa-miR-195-5p,1147, 4790, 4792, 5970, 7189, hsa-miR-122-5p, hsa-miR-124-3p, hsa-miR-145-5p, hsa-miR-146a-5p, hsa-miR-146b-5p, hsa-miR-155-5p, hsa-miR-195-5p, hsa-miR-221-3p, hsa-miR-26a-5p, hsa-miR-34a-5p,1147, 1387, 2033, 3091, 405, 4790, 4792, 5970, hsa-miR-122-5p, hsa-miR-124-3p, hsa-miR-145-5p, hsa-miR-146a-5p, hsa-miR-17-5p, hsa-miR-195-5p, hsa-miR-203a, hsa-miR-20a-5p, hsa-miR-21-5p, hsa-miR-221-3p, hsa-miR-26a-5p, hsa-miR-29b-3p, hsa-miR-34a-5p, hsa-miR-34b-5p,10000, 1147, 1387, 2033, 207, 208, 3091, 405, 4790, 4792, 5970, hsa-miR-122-5p, hsa-miR-124-3p, hsa-miR-145-5p, hsa-miR-17-5p, hsa-miR-195-5p, hsa-miR-203a, hsa-miR-20a-5p, hsa-miR-21-5p, hsa-miR-221-3p, hsa-miR-26a-5p, hsa-miR-29b-3p, hsa-miR-34a-5p, hsa-miR-34b-5p,10000, 1147, 1387, 2033, 207, 208, 3091, 405, 4790, 4792, 5728, 5970, hsa-miR-106b-5p, hsa-miR-122-5p, hsa-miR-124-3p, hsa-miR-17-5p, hsa-miR-195-5p, hsa-miR-19a-3p, hsa-miR-203a, hsa-miR-20a-5p, hsa-miR-21-5p, hsa-miR-221-3p, hsa-miR-222-3p, hsa-miR-26a-5p, hsa-miR-29a-3p, hsa-miR-29b-3p, hsa-miR-34a-5p, hsa-miR-34b-5p, hsa-miR-494, hsa-miR-93-5p,10000, 1147, 207, 208, 4790, 4792, 5728, 5970, hsa-miR-106b-5p, hsa-miR-124-3p, hsa-miR-15a-5p, hsa-miR-16-5p, hsa-miR-17-5p, hsa-miR-195-5p, hsa-miR-19a-3p, hsa-miR-203a, hsa-miR-20a-5p, hsa-miR-21-5p, hsa-miR-221-3p, hsa-miR-222-3p, hsa-miR-24-3p, hsa-miR-26a-5p, hsa-miR-29a-3p, hsa-miR-29b-3p, hsa-miR-34a-5p, hsa-miR-34b-5p, hsa-miR-34c-5p, hsa-miR-449a, hsa-miR-494, hsa-miR-503-5p, hsa-miR-93-5p,10000, 1147, 207, 208, 4149, 4609, 4790, 5728, 5970, hsa-miR-106b-5p, hsa-miR-124-3p, hsa-miR-15a-5p, hsa-miR-16-5p, hsa-miR-17-5p, hsa-miR-195-5p, hsa-miR-19a-3p, hsa-miR-203a, hsa-miR-20a-5p, hsa-miR-21-5p, hsa-miR-24-3p, hsa-miR-26a-5p, hsa-miR-29a-3p, hsa-miR-29b-3p, hsa-miR-34a-5p, hsa-miR-34b-5p, hsa-miR-34c-5p, hsa-miR-449a, hsa-miR-494, hsa-miR-503-5p, hsa-miR-93-5p,10000, 1147, 207, 208, 4149, 4609, 5728, hsa-miR-106b-5p, hsa-miR-15a-5p, hsa-miR-16-5p, hsa-miR-17-5p, hsa-miR-203a, hsa-miR-20a-5p, hsa-miR-21-5p, hsa-miR-24-3p, hsa-miR-34a-5p, hsa-miR-34b-5p, hsa-miR-34c-5p, hsa-miR-93-5p, hsa-miR-98-5p,10000, 1147, 207, 208, 5728, hsa-miR-106b-5p, hsa-miR-15a-5p, hsa-miR-16-5p, hsa-miR-17-5p, hsa-miR-203a, hsa-miR-20a-5p, hsa-miR-21-5p, hsa-miR-223-3p, hsa-miR-93-5p, hsa-miR-98-5p,10000, 207, 208, 5728, hsa-miR-106b-5p, hsa-miR-149-3p, hsa-miR-17-5p, hsa-miR-203a, hsa-miR-20a-5p, hsa-miR-21-5p, hsa-miR-223-3p, hsa-miR-93-5p, hsa-miR-98-5p,1869, hsa-miR-106a-5p, hsa-miR-106b-5p, hsa-miR-149-3p, hsa-miR-17-5p, hsa-miR-203a, hsa-miR-20a-5p, hsa-miR-21-5p, hsa-miR-223-3p, hsa-miR-93-5p, hsa-miR-98-5p |
| 82.89 | 26084, 391,391, 9844,1398, 1399, 1793, 9844,1398, 1399, 2549, 5296,5296, 998, hsa-miR-185-5p,10332, 23365, 30835, 387, hsa-miR-122-5p, hsa-miR-155-5p, hsa-miR-185-5p, hsa-miR-31-5p,387, 5894, hsa-miR-122-5p, hsa-miR-155-5p, hsa-miR-195-5p, hsa-miR-31-5p,5894, hsa-miR-122-5p, hsa-miR-124-3p, hsa-miR-155-5p, hsa-miR-195-5p, hsa-miR-31-5p,4790, 4792, 5894, 5970, 7189, hsa-miR-122-5p, hsa-miR-124-3p, hsa-miR-146a-5p, hsa-miR-146b-5p, hsa-miR-155-5p, hsa-miR-195-5p,1147, 4790, 4792, 5970, 7189, hsa-miR-122-5p, hsa-miR-124-3p, hsa-miR-145-5p, hsa-miR-146a-5p, hsa-miR-146b-5p, hsa-miR-155-5p, hsa-miR-195-5p, hsa-miR-221-3p, hsa-miR-26a-5p, hsa-miR-34a-5p,1147, 1387, 2033, 3091, 405, 4790, 4792, 5970, hsa-miR-122-5p, hsa-miR-124-3p, hsa-miR-145-5p, hsa-miR-146a-5p, hsa-miR-17-5p, hsa-miR-195-5p, hsa-miR-203a, hsa-miR-20a-5p, hsa-miR-21-5p, hsa-miR-221-3p, hsa-miR-26a-5p, hsa-miR-29b-3p, hsa-miR-34a-5p, hsa-miR-34b-5p,10000, 1147, 1387, 2033, 207, 208, 3091, 405, 4790, 4792, 5970, hsa-miR-122-5p, hsa-miR-124-3p, hsa-miR-145-5p, hsa-miR-17-5p, hsa-miR-195-5p, hsa-miR-203a, hsa-miR-20a-5p, hsa-miR-21-5p, hsa-miR-221-3p, hsa-miR-26a-5p, hsa-miR-29b-3p, hsa-miR-34a-5p, hsa-miR-34b-5p,10000, 1147, 1387, 2033, 207, 208, 3091, 405, 4790, 4792, 5728, 5970, hsa-miR-106b-5p, hsa-miR-122-5p, hsa-miR-124-3p, hsa-miR-17-5p, hsa-miR-195-5p, hsa-miR-19a-3p, hsa-miR-203a, hsa-miR-20a-5p, hsa-miR-21-5p, hsa-miR-221-3p, hsa-miR-222-3p, hsa-miR-26a-5p, hsa-miR-29a-3p, hsa-miR-29b-3p, hsa-miR-34a-5p, hsa-miR-34b-5p, hsa-miR-494, hsa-miR-93-5p,10000, 1147, 207, 208, 4790, 4792, 5728, 5970, hsa-miR-106b-5p, hsa-miR-124-3p, hsa-miR-15a-5p, hsa-miR-16-5p, hsa-miR-17-5p, hsa-miR-195-5p, hsa-miR-19a-3p, hsa-miR-203a, hsa-miR-20a-5p, hsa-miR-21-5p, hsa-miR-221-3p, hsa-miR-222-3p, hsa-miR-24-3p, hsa-miR-26a-5p, hsa-miR-29a-3p, hsa-miR-29b-3p, hsa-miR-34a-5p, hsa-miR-34b-5p, hsa-miR-34c-5p, hsa-miR-449a, hsa-miR-494, hsa-miR-503-5p, hsa-miR-93-5p,10000, 1027, 1147, 207, 208, 4792, hsa-miR-124-3p, hsa-miR-15a-5p, hsa-miR-16-5p, hsa-miR-221-3p, hsa-miR-222-3p, hsa-miR-24-3p, hsa-miR-26a-5p, hsa-miR-34a-5p, hsa-miR-34b-5p, hsa-miR-34c-5p, hsa-miR-449a, hsa-miR-503-5p,10000, 1027, 1147, 207, 208, 4792, hsa-miR-218-5p, hsa-miR-221-3p, hsa-miR-222-3p, hsa-miR-24-3p,10000, 1147, 207, 208, 3551, 4792, hsa-miR-218-5p,10000, 1147, 207, 208, 3551, 4792, 8517 |

**Table S4. 122 Deregulated Pathway elements of Primary Myelofibrosis compared to bone marrow samples.**

| **Entrez Gene** | **Gene Symbol** |
| --- | --- |
| 10000 | AKT3 |
| 10332 | CLEC4M |
| 10368 | CACNG3 |
| 10369 | CACNG2 |
| 1147 | CHUK |
| 116443 | GRIN3A |
| 116444 | GRIN3B |
| 1387 | CREBBP |
| 1398 | CRK |
| 1399 | CRKL |
| 1793 | DOCK1 |
| 2023 | ENO1 |
| 2026 | ENO2 |
| 2027 | ENO3 |
| 2033 | EP300 |
| 2056 | EPO |
| 207 | AKT1 |
| 208 | AKT2 |
| 23365 | ARHGEF12 |
| 2549 | GAB1 |
| 26084 | ARHGEF26 |
| 27091 | CACNG5 |
| 27092 | CACNG4 |
| 284 | ANGPT1 |
| 285 | ANGPT2 |
| 2890 | GRIA1 |
| 2892 | GRIA3 |
| 2893 | GRIA4 |
| 2902 | GRIN1 |
| 2904 | GRIN2B |
| 2905 | GRIN2C |
| 2906 | GRIN2D |
| 30835 | CD209 |
| 3091 | HIF1A |
| 3098 | HK1 |
| 3101 | HK3 |
| 387 | RHOA |
| 391 | RHOG |
| 3939 | LDHA |
| 405 | ARNT |
| 4055 | LTBR |
| 4790 | NFKB1 |
| 4792 | NFKBIA |
| 4846 | NOS3 |
| 4878 | NPPA |
| 51378 | ANGPT4 |
| 5163 | PDK1 |
| 5207 | PFKFB1 |
| 5208 | PFKFB2 |
| 5209 | PFKFB3 |
| 5210 | PFKFB4 |
| 5211 | PFKL |
| 5228 | PGF |
| 5230 | PGK1 |
| 5296 | PIK3R2 |
| 5350 | PLN |
| 5499 | PPP1CA |
| 5566 | PRKACA |
| 5567 | PRKACB |
| 5568 | PRKACG |
| 55799 | CACNA2D3 |
| 5613 | PRKX |
| 5728 | PTEN |
| 5894 | RAF1 |
| 59283 | CACNG8 |
| 59284 | CACNG7 |
| 59285 | CACNG6 |
| 5970 | RELA |
| 6262 | RYR2 |
| 6513 | SLC2A1 |
| 7010 | TEK |
| 7018 | TF |
| 7076 | TIMP1 |
| 7189 | TRAF6 |
| 7423 | VEGFB |
| 7424 | VEGFC |
| 775 | CACNA1C |
| 776 | CACNA1D |
| 778 | CACNA1F |
| 779 | CACNA1S |
| 781 | CACNA2D1 |
| 782 | CACNB1 |
| 783 | CACNB2 |
| 784 | CACNB3 |
| 785 | CACNB4 |
| 786 | CACNG1 |
| 80201 | HKDC1 |
| 9254 | CACNA2D2 |
| 93589 | CACNA2D4 |
| 9844 | ELMO1 |
| 998 | CDC42 |
| hsa-miR-106b-5p | hsa-miR-106b-5p |
| hsa-miR-122-5p | hsa-miR-122-5p |
| hsa-miR-124-3p | hsa-miR-124-3p |
| hsa-miR-1244 | hsa-miR-1244 |
| hsa-miR-125b-5p | hsa-miR-125b-5p |
| hsa-miR-133a | hsa-miR-133a |
| hsa-miR-145-5p | hsa-miR-145-5p |
| hsa-miR-146a-5p | hsa-miR-146a-5p |
| hsa-miR-146b-5p | hsa-miR-146b-5p |
| hsa-miR-155-5p | hsa-miR-155-5p |
| hsa-miR-17-5p | hsa-miR-17-5p |
| hsa-miR-182-5p | hsa-miR-182-5p |
| hsa-miR-185-5p | hsa-miR-185-5p |
| hsa-miR-195-5p | hsa-miR-195-5p |
| hsa-miR-19a-3p | hsa-miR-19a-3p |
| hsa-miR-203a | hsa-miR-203a |
| hsa-miR-20a-5p | hsa-miR-20a-5p |
| hsa-miR-21-5p | hsa-miR-21-5p |
| hsa-miR-22-3p | hsa-miR-22-3p |
| hsa-miR-221-3p | hsa-miR-221-3p |
| hsa-miR-222-3p | hsa-miR-222-3p |
| hsa-miR-26a-5p | hsa-miR-26a-5p |
| hsa-miR-29a-3p | hsa-miR-29a-3p |
| hsa-miR-29b-3p | hsa-miR-29b-3p |
| hsa-miR-31-5p | hsa-miR-31-5p |
| hsa-miR-34a-5p | hsa-miR-34a-5p |
| hsa-miR-34b-5p | hsa-miR-34b-5p |
| hsa-miR-375 | hsa-miR-375 |
| hsa-miR-494 | hsa-miR-494 |
| hsa-miR-519d | hsa-miR-519d |
| hsa-miR-93-5p | hsa-miR-93-5p |

**2) Micrographite results of Primary Myelofibrosis samples compared with peripheral blood of healthy donors**

**Table S5. Micrographite whole pathway analysis results.**

| **Pathway Name** | **p-value mean test** | **p-value variance test** | **adjusted**  **p-value mean test** | **adjusted**  **p-value variance test** |
| --- | --- | --- | --- | --- |
| Adipocytokine signaling pathway | 0 | 0 | 0 | 0 |
| African trypanosomiasis | 0 | 0 | 0 | 0 |
| Alcoholism | 0 | 0 | 0 | 0 |
| Aldosterone-regulated sodium reabsorption | 0 | 0 | 0 | 0 |
| Allograft rejection | 0 | 0 | 0 | 0 |
| Alzheimer's disease | 0 | 0 | 0 | 0 |
| Amino sugar and nucleotide sugar metabolism | 0 | 0 | 0 | 0 |
| Aminoacyl-tRNA biosynthesis | 0 | 0 | 0 | 0 |
| Amoebiasis | 0 | 0 | 0 | 0 |
| Amyotrophic lateral sclerosis (ALS) | 0 | 0 | 0 | 0 |
| Antigen processing and presentation | 0 | 0 | 0 | 0 |
| Apoptosis | 0 | 0 | 0 | 0 |
| Arginine and proline metabolism | 0 | 0 | 0 | 0 |
| Arrhythmogenic right ventricular cardiomyopathy (ARVC) | 0 | 0 | 0 | 0 |
| Asthma | 0 | 0 | 0 | 0 |
| Autoimmune thyroid disease | 0 | 0 | 0 | 0 |
| Axon guidance | 0 | 0 | 0 | 0 |
| B cell receptor signaling pathway | 0 | 0 | 0 | 0 |
| Bacterial invasion of epithelial cells | 0 | 0 | 0 | 0 |
| Bile secretion | 0 | 0 | 0 | 0 |
| Biotin metabolism | 0 | 0 | 0 | 0 |
| Bladder cancer | 0 | 0 | 0 | 0 |
| Butirosin and neomycin biosynthesis | 0 | 0 | 0 | 0 |
| Carbohydrate digestion and absorption | 0 | 0 | 0 | 0 |
| Cell adhesion molecules (CAMs) | 0 | 0 | 0 | 0 |
| Chagas disease (American trypanosomiasis) | 0 | 0 | 0 | 0 |
| Cholinergic synapse | 0 | 0 | 0 | 0 |
| Cocaine addiction | 0 | 0 | 0 | 0 |
| Colorectal cancer | 0 | 0 | 0 | 0 |
| Complement and coagulation cascades | 0 | 0 | 0 | 0 |
| Cyanoamino acid metabolism | 0 | 0 | 0 | 0 |
| Cysteine and methionine metabolism | 0 | 0 | 0 | 0 |
| Cytosolic DNA-sensing pathway | 0 | 0 | 0 | 0 |
| Dilated cardiomyopathy | 0 | 0 | 0 | 0 |
| Dorso-ventral axis formation | 0 | 0 | 0 | 0 |
| Drug metabolism - other enzymes | 0 | 0 | 0 | 0 |
| Endocrine and other factor-regulated calcium reabsorption | 0 | 0 | 0 | 0 |
| Endometrial cancer | 0 | 0 | 0 | 0 |
| Epithelial cell signaling in Helicobacter pylori infection | 0 | 0 | 0 | 0 |
| ErbB signaling pathway | 0 | 0 | 0 | 0 |
| Fatty acid elongation | 0 | 0 | 0 | 0 |
| Fc gamma R-mediated phagocytosis | 0 | 0 | 0 | 0 |
| Folate biosynthesis | 0 | 0 | 0 | 0 |
| Fructose and mannose metabolism | 0 | 0 | 0 | 0 |
| Galactose metabolism | 0 | 0 | 0 | 0 |
| Gap junction | 0 | 0 | 0 | 0 |
| Gastric acid secretion | 0 | 0 | 0 | 0 |
| Glioma | 0 | 0 | 0 | 0 |
| Glycine, serine and threonine metabolism | 0 | 0 | 0 | 0 |
| Glycolysis / Gluconeogenesis | 0 | 0 | 0 | 0 |
| Glycosaminoglycan biosynthesis - chondroitin sulfate / dermatan sulfate | 0 | 0 | 0 | 0 |
| Glycosaminoglycan degradation | 0 | 0 | 0 | 0 |
| Glycosylphosphatidylinositol(GPI)-anchor biosynthesis | 0 | 0 | 0 | 0 |
| Glyoxylate and dicarboxylate metabolism | 0 | 0 | 0 | 0 |
| GnRH signaling pathway | 0 | 0 | 0 | 0 |
| HIF-1 signaling pathway | 0 | 0 | 0 | 0 |
| HTLV-I infection | 0 | 0 | 0 | 0 |
| Hepatitis B | 0 | 0 | 0 | 0 |
| Hepatitis C | 0 | 0 | 0 | 0 |
| Herpes simplex infection | 0 | 0 | 0 | 0 |
| Histidine metabolism | 0 | 0 | 0 | 0 |
| Huntington's disease | 0 | 0 | 0 | 0 |
| Hypertrophic cardiomyopathy (HCM) | 0 | 0 | 0 | 0 |
| Influenza A | 0 | 0 | 0 | 0 |
| Insulin signaling pathway | 0 | 0 | 0 | 0 |
| Legionellosis | 0 | 0 | 0 | 0 |
| Leishmaniasis | 0 | 0 | 0 | 0 |
| Leukocyte transendothelial migration | 0 | 0 | 0 | 0 |
| Malaria | 0 | 0 | 0 | 0 |
| Measles | 0 | 0 | 0 | 0 |
| N-Glycan biosynthesis | 0 | 0 | 0 | 0 |
| NF-kappa B signaling pathway | 0 | 0 | 0 | 0 |
| NOD-like receptor signaling pathway | 0 | 0 | 0 | 0 |
| Neuroactive ligand-receptor interaction | 0 | 0 | 0 | 0 |
| Non-small cell lung cancer | 0 | 0 | 0 | 0 |
| Oocyte meiosis | 0 | 0 | 0 | 0 |
| Osteoclast differentiation | 0 | 0 | 0 | 0 |
| PPAR signaling pathway | 0 | 0 | 0 | 0 |
| Pancreatic cancer | 0 | 0 | 0 | 0 |
| Pancreatic secretion | 0 | 0 | 0 | 0 |
| Pantothenate and CoA biosynthesis | 0 | 0 | 0 | 0 |
| Parkinson's disease | 0 | 0 | 0 | 0 |
| Pertussis | 0 | 0 | 0 | 0 |
| Phenylalanine metabolism | 0 | 0 | 0 | 0 |
| Porphyrin and chlorophyll metabolism | 0 | 0 | 0 | 0 |
| Primary bile acid biosynthesis | 0 | 0 | 0 | 0 |
| Prion diseases | 0 | 0 | 0 | 0 |
| Prostate cancer | 0 | 0 | 0 | 0 |
| RIG-I-like receptor signaling pathway | 0 | 0 | 0 | 0 |
| Regulation of actin cytoskeleton | 0 | 0 | 0 | 0 |
| Renal cell carcinoma | 0 | 0 | 0 | 0 |
| Rheumatoid arthritis | 0 | 0 | 0 | 0 |
| Riboflavin metabolism | 0 | 0 | 0 | 0 |
| Salivary secretion | 0 | 0 | 0 | 0 |
| Salmonella infection | 0 | 0 | 0 | 0 |
| Selenocompound metabolism | 0 | 0 | 0 | 0 |
| Shigellosis | 0 | 0 | 0 | 0 |
| Small cell lung cancer | 0 | 0 | 0 | 0 |
| Staphylococcus aureus infection | 0 | 0 | 0 | 0 |
| Starch and sucrose metabolism | 0 | 0 | 0 | 0 |
| Sulfur metabolism | 0 | 0 | 0 | 0 |
| Synaptic vesicle cycle | 0 | 0 | 0 | 0 |
| Systemic lupus erythematosus | 0 | 0 | 0 | 0 |
| T cell receptor signaling pathway | 0 | 0 | 0 | 0 |
| Taurine and hypotaurine metabolism | 0 | 0 | 0 | 0 |
| Terpenoid backbone biosynthesis | 0 | 0 | 0 | 0 |
| Thyroid cancer | 0 | 0 | 0 | 0 |
| Toll-like receptor signaling pathway | 0 | 0 | 0 | 0 |
| Toxoplasmosis | 0 | 0 | 0 | 0 |
| Tuberculosis | 0 | 0 | 0 | 0 |
| Type II diabetes mellitus | 0 | 0 | 0 | 0 |
| Vascular smooth muscle contraction | 0 | 0 | 0 | 0 |
| Vasopressin-regulated water reabsorption | 0 | 0 | 0 | 0 |
| Vibrio cholerae infection | 0 | 0 | 0 | 0 |
| Viral myocarditis | 0 | 0 | 0 | 0 |
| mTOR signaling pathway | 0 | 0 | 0 | 0 |
| p53 signaling pathway | 0 | 0 | 0 | 0 |
| Cell cycle | 0 | 0.01 | 0 | 0.015864662 |
| Chemokine signaling pathway | 0 | 0.01 | 0 | 0.015864662 |
| Fat digestion and absorption | 0 | 0.01 | 0 | 0.015864662 |
| Glutamatergic synapse | 0 | 0.01 | 0 | 0.015864662 |
| Graft-versus-host disease | 0 | 0.01 | 0 | 0.015864662 |
| Melanogenesis | 0 | 0.01 | 0 | 0.015864662 |
| Mineral absorption | 0 | 0.01 | 0 | 0.015864662 |
| Natural killer cell mediated cytotoxicity | 0 | 0.01 | 0 | 0.015864662 |
| Notch signaling pathway | 0 | 0.01 | 0 | 0.015864662 |
| Pathogenic Escherichia coli infection | 0 | 0.01 | 0 | 0.015864662 |
| Proximal tubule bicarbonate reclamation | 0 | 0.01 | 0 | 0.015864662 |
| Steroid biosynthesis | 0 | 0.01 | 0 | 0.015864662 |
| Transcriptional misregulation in cancer | 0 | 0.01 | 0 | 0.015864662 |
| Ubiquinone and other terpenoid-quinone biosynthesis | 0 | 0.01 | 0 | 0.015864662 |
| VEGF signaling pathway | 0 | 0.01 | 0 | 0.015864662 |
| beta-Alanine metabolism | 0 | 0.01 | 0 | 0.015864662 |
| Arachidonic acid metabolism | 0 | 0.02 | 0 | 0.02951049 |
| Butanoate metabolism | 0 | 0.02 | 0 | 0.02951049 |
| Citrate cycle (TCA cycle) | 0 | 0.02 | 0 | 0.02951049 |
| Fc epsilon RI signaling pathway | 0 | 0.02 | 0 | 0.02951049 |
| Neurotrophin signaling pathway | 0 | 0.02 | 0 | 0.02951049 |
| Phenylalanine, tyrosine and tryptophan biosynthesis | 0 | 0.02 | 0 | 0.02951049 |
| Phototransduction | 0 | 0.02 | 0 | 0.02951049 |
| Propanoate metabolism | 0 | 0.02 | 0 | 0.02951049 |
| Tight junction | 0 | 0.02 | 0 | 0.02951049 |
| Tryptophan metabolism | 0 | 0.02 | 0 | 0.02951049 |
| Adherens junction | 0 | 0.03 | 0 | 0.042483221 |
| Chronic myeloid leukemia | 0 | 0.03 | 0 | 0.042483221 |
| Glycosphingolipid biosynthesis - globo series | 0 | 0.03 | 0 | 0.042483221 |
| Intestinal immune network for IgA production | 0 | 0.03 | 0 | 0.042483221 |
| Melanoma | 0 | 0.03 | 0 | 0.042483221 |
| Serotonergic synapse | 0 | 0.03 | 0 | 0.042483221 |
| Jak-STAT signaling pathway | 0 | 0.04 | 0 | 0.055526316 |
| Morphine addiction | 0 | 0.04 | 0 | 0.055526316 |
| Vitamin B6 metabolism | 0 | 0.04 | 0 | 0.055526316 |
| D-Glutamine and D-glutamate metabolism | 0 | 0.05 | 0 | 0.067197452 |
| Glutathione metabolism | 0 | 0.05 | 0 | 0.067197452 |
| Hedgehog signaling pathway | 0 | 0.05 | 0 | 0.067197452 |
| Inositol phosphate metabolism | 0 | 0.05 | 0 | 0.067197452 |
| Pentose phosphate pathway | 0 | 0.05 | 0 | 0.067197452 |
| Focal adhesion | 0 | 0.06 | 0 | 0.079125 |
| Long-term depression | 0 | 0.06 | 0 | 0.079125 |
| Retrograde endocannabinoid signaling | 0 | 0.06 | 0 | 0.079125 |
| Amphetamine addiction | 0 | 0.07 | 0 | 0.090613497 |
| Dopaminergic synapse | 0 | 0.07 | 0 | 0.090613497 |
| ECM-receptor interaction | 0 | 0.07 | 0 | 0.090613497 |

**Table S6. Results of the micrographite analysis of the paths in the pathways**. The paths presented in the following table have been selected to compose the meta-pathway.

| **Name of the pathway** | **Max Score** | **Involved Genes and miRNAs** |
| --- | --- | --- |
| Dilated cardiomyopathy | 117.9 | 112, 2778, hsa-miR-182-5p, hsa-miR-96-5p, 107, 108, 109, 111, 112, 113, 114, 115, 196883, 2778, 107, 108, 109, 111, 112, 113, 114, 115, 196883, 5566, hsa-miR-146b-5p, 107, 108, 109, 111, 112, 113, 114, 115, 196883, 5566, 5567, 5568, 5613, 5566, 5567, 5568, 5613, 775, hsa-miR-133a, 5350, 5566, 5567, 5568, 5613, 10368, 5566, 5567, 5568, 5613, 10369, 5566, 5567, 5568, 5613, 27091, 5566, 5567, 5568, 5613, 27092, 5566, 5567, 5568, 5613, 5566, 5567, 5568, 55799, 5613, 5566, 5567, 5568, 5613, 59283, 5566, 5567, 5568, 5613, 59284, 5566, 5567, 5568, 5613, 59285, 5566, 5567, 5568, 5613, 6262, 5566, 5567, 5568, 5613, 776, 5566, 5567, 5568, 5613, 778, 5566, 5567, 5568, 5613, 779, 5566, 5567, 5568, 5613, 781, 5566, 5567, 5568, 5613, 782, 5566, 5567, 5568, 5613, 783, 5566, 5567, 5568, 5613, 784, 5566, 5567, 5568, 5613, 785, 5566, 5567, 5568, 5613, 786, 5566, 5567, 5568, 5613, 9254, 5566, 5567, 5568, 5613, 93589 |
| Tuberculosis | 99.0 | 3458, 3459, hsa-miR-155-5p, 10332, 23365, 30835, 387, hsa-miR-122-5p, hsa-miR-155-5p, hsa-miR-185-5p, hsa-miR-31-5p, 10332, 30835, 387, 5600, 572, 7189, 8767, hsa-miR-122-5p, hsa-miR-155-5p, 10332, 30835, 387, 5600, 572, 5894, 7189, 8767, hsa-miR-155-5p, hsa-miR-195-5p, 10332, 1432, 30835, 5600, 572, 5894, 7189, 8767, hsa-miR-155-5p, hsa-miR-195-5p, hsa-miR-29b-3p, 1432, 4790, 5600, 572, 5894, 5970, 6772, 7189, 8767, hsa-miR-155-5p, hsa-miR-195-5p, hsa-miR-29b-3p, hsa-miR-34a-5p, hsa-miR-34b-5p, 4261, 4790, 5970, 6772, hsa-miR-155-5p, hsa-miR-34a-5p, hsa-miR-34b-5p, 1385, 4261, hsa-miR-103a-3p, hsa-miR-182-5p, hsa-miR-203a, hsa-miR-34b-5p, 1385, 4261, 4800, 4801, 4802, 5993, 5994, 8625, 1385, 3108, 4800, 4801, 4802, 5993, 5994, 8625, hsa-miR-638, 1385, 3109, 4800, 4801, 4802, 5993, 5994, 8625, 1385, 3111, 4800, 4801, 4802, 5993, 5994, 8625, 1385, 3112, 4800, 4801, 4802, 5993, 5994, 8625, 1385, 3113, 4800, 4801, 4802, 5993, 5994, 8625, 1385, 3115, 4800, 4801, 4802, 5993, 5994, 8625, 1385, 3117, 4800, 4801, 4802, 5993, 5994, 8625, 1385, 3118, 4800, 4801, 4802, 5993, 5994, 8625, 1385, 3119, 4800, 4801, 4802, 5993, 5994, 8625, 1385, 3122, 4800, 4801, 4802, 5993, 5994, 8625, 1385, 3123, 4800, 4801, 4802, 5993, 5994, 8625, 1385, 3127, 4800, 4801, 4802, 5993, 5994, 8625, 1385, 4800, 4801, 4802, 5993, 5994, 8625, 972 |
| Toll-like receptor signaling pathway | 77.5 | 10454, 7189, 8737, hsa-miR-155-5p, 114609, 148022, 3654, 4615, 7189, 8737, hsa-miR-146a-5p, hsa-miR-146b-5p, hsa-miR-155-5p, 114609, 1147, 1326, 148022, 3654, 4615, 5594, 5595, 7189, hsa-miR-146a-5p, hsa-miR-146b-5p, hsa-miR-155-5p, 114609, 1147, 1326, 148022, 4615, 5594, 5595, 7189, hsa-miR-146a-5p, hsa-miR-146b-5p, hsa-miR-155-5p, hsa-miR-221-3p, 114609, 1147, 1326, 1432, 4615, 5594, 5595, 5600, 5603, 5606, 6300, 7189, hsa-miR-146a-5p, hsa-miR-146b-5p, hsa-miR-155-5p, hsa-miR-221-3p, 1147, 1326, 1432, 2353, 5594, 5595, 5599, 5600, 5601, 5602, 5603, 5606, 6300, 7189, hsa-miR-146a-5p, hsa-miR-146b-5p, hsa-miR-155-5p, hsa-miR-221-3p, 1147, 1326, 1432, 2353, 3725, 5594, 5595, 5599, 5600, 5601, 5602, 5603, 5606, 6300, 7189, hsa-miR-146a-5p, hsa-miR-146b-5p, hsa-miR-155-5p, 1147, 1326, 2353, 3725, 5599, 5601, 5602, 5606, 7189, hsa-miR-146a-5p, hsa-miR-146b-5p, hsa-miR-17-5p, 1147, 1326, 2353, 3725, 4790, 4792, 7189, hsa-miR-146a-5p, hsa-miR-146b-5p, hsa-miR-17-5p, 2353, 3663, 3725, 4790, 4792, 7189, hsa-miR-146a-5p, hsa-miR-17-5p, 2353, 3663, 3725, 4790, 4792, 5970, hsa-miR-17-5p, 2353, 3576, 3663, 3725, 4790, 5970, hsa-miR-17-5p, hsa-miR-520b, 2353, 3663, 3725, 4790, 5970, 7124, hsa-miR-19a-3p, 2353, 3553, 3663, 3725, 4790, 5970, hsa-miR-21-5p, 2353, 3569, 3663, 3725, 4790, 5970, hsa-let-7a-5p, 2353, 3592, 3663, 3725, 4790, 5970, 2353, 3593, 3663, 3725, 4790, 5970, 2353, 3663, 3725, 4790, 5970, 6348, 2353, 3663, 3725, 4790, 5970, 6351, 2353, 3663, 3725, 4790, 5970, 6352 |
| Amphetamine addiction | 75.9 | 6531, 815, 816, 817, 818, hsa-miR-34a-5p, 23411, 2354, 3065, 5499, 5500, 5501, 815, 816, 817, 818, hsa-miR-34a-5p, 23411, 2354, 3065, 5499, 5500, 5501, hsa-miR-181b-5p, hsa-miR-34a-5p, 5499, 5500, 5501, hsa-miR-125b-5p, hsa-miR-181b-5p, 5566, 5567, 5568, 5613, hsa-miR-125b-5p, hsa-miR-181b-5p, 2903, 5566, 5567, 5568, 5613, hsa-miR-125b-5p, 5566, 5567, 5568, 5613, 775, hsa-miR-133a, 111, 5566, 5567, 5568, 5613, 116443, 5566, 5567, 5568, 5613, 116444, 5566, 5567, 5568, 5613, 2890, 5566, 5567, 5568, 5613, 2892, 5566, 5567, 5568, 5613, 2893, 5566, 5567, 5568, 5613, 2902, 5566, 5567, 5568, 5613, 2904, 5566, 5567, 5568, 5613, 2905, 5566, 5567, 5568, 5613, 2906, 5566, 5567, 5568, 5613, 5566, 5567, 5568, 5613, 776 |
| Fc gamma R-mediated phagocytosis | 72.9 | 2213, hsa-miR-155-5p, hsa-miR-184, 10000, 207, 208, 23533, 5290, 5291, 5293, 5294, 5295, 5296, 8503, hsa-miR-155-5p, hsa-miR-184, 10000, 207, 208, 23533, 4651, 5290, 5291, 5293, 5294, 5295, 5296, 8503, hsa-miR-155-5p, 10000, 207, 208, 23533, 382, 5290, 5291, 5293, 5294, 5295, 5296, 5337, 5338, 5580, 5581, 6850, 8503, 8611, 8612, 8613, 9846, hsa-miR-122-5p, hsa-miR-126-3p, hsa-miR-29a-3p, hsa-miR-29b-3p, hsa-miR-29c-3p, 23396, 382, 5337, 5338, 5580, 5581, 5879, 5880, 6850, 7454, 8394, 8395, 8611, 8612, 8613, 8976, 9846, hsa-miR-122-5p, hsa-miR-126-3p, hsa-miR-29a-3p, hsa-miR-29b-3p, hsa-miR-29c-3p, 10451, 23396, 5580, 5581, 5879, 5880, 6850, 7409, 7410, 7454, 8394, 8395, 8976, 9846, hsa-miR-122-5p, hsa-miR-126-3p, hsa-miR-29a-3p, hsa-miR-29b-3p, hsa-miR-29c-3p, 10451, 1794, 5580, 5581, 5879, 5880, 6850, 7409, 7410, 7454, 8976, 9846, hsa-miR-122-5p, hsa-miR-126-3p, 1794, 5580, 5581, 5879, 5880, 5894, 6850, 7454, 8976, 9846, hsa-miR-126-3p, 1794, 5879, 5880, 5894, 6850, 7454, 8976, 9846, hsa-miR-126-3p, hsa-miR-335-5p, 1794, 2212, 2214, 5894, 6850, 9846, hsa-miR-126-3p, hsa-miR-335-5p, 2212, 2214, 5894, 6850, hsa-miR-335-5p, hsa-miR-762, 5604, 5894, hsa-miR-335-5p, hsa-miR-762, 5594, 5604, hsa-miR-199b-3p, hsa-miR-28-5p, hsa-miR-335-5p, hsa-miR-762, 5594, 5595, 5604, 123745, 5594, 5595, 255189, 5594, 5595, 283748, 5594, 5595, 5321, 5594, 5595 |
| Regulation of actin cytoskeleton | 72.5 | 1128, 23365, 55970, 8874, 9138, 9459, 23365, 387, 50649, 55970, 6714, 8874, 9138, 9459, hsa-miR-122-5p, hsa-miR-185-5p, hsa-miR-31-5p, 387, 50649, 55970, 6714, 8874, 9459, 998, hsa-miR-122-5p, hsa-miR-185-5p, hsa-miR-29a-3p, hsa-miR-29b-3p, hsa-miR-29c-3p, hsa-miR-31-5p, 10451, 22808, 3265, 3845, 387, 4893, 50649, 55970, 6237, 6714, 7074, 7409, 7410, 8874, 9459, 998, hsa-miR-122-5p, hsa-miR-126-3p, hsa-miR-21-5p, hsa-miR-29a-3p, hsa-miR-29b-3p, hsa-miR-29c-3p, hsa-miR-31-5p, 10451, 22801, 22808, 3265, 3845, 387, 4893, 50649, 55970, 6237, 6714, 7074, 7409, 7410, 8874, 9459, 998, hsa-miR-122-5p, hsa-miR-126-3p, hsa-miR-21-5p, hsa-miR-29a-3p, hsa-miR-31-5p, 10451, 1398, 1399, 22801, 22808, 3265, 3680, 3845, 387, 4893, 50649, 55970, 5747, 5879, 5880, 5881, 6237, 6714, 7074, 7409, 7410, 8874, 9459, 9564, 998, hsa-miR-122-5p, hsa-miR-126-3p, hsa-miR-21-5p, hsa-miR-31-5p, 10298, 1398, 1399, 22801, 22808, 3265, 3680, 3845, 387, 4893, 5058, 5062, 5063, 55970, 56924, 57144, 5747, 5879, 5880, 5881, 6237, 6714, 8874, 9459, 9564, 998, hsa-miR-126-3p, hsa-miR-21-5p, hsa-miR-31-5p, 10298, 1398, 1399, 22801, 22808, 3265, 3680, 3688, 3690, 3696, 3845, 387, 4893, 5058, 5062, 5063, 55970, 56924, 57144, 5747, 5879, 5880, 5881, 6237, 6714, 9564, 998, hsa-miR-126-3p, hsa-miR-138-5p, hsa-miR-21-5p, hsa-miR-31-5p, 10298, 2263, 22808, 3265, 3688, 3690, 3696, 3845, 387, 4893, 5058, 5062, 5063, 5594, 55970, 5604, 5605, 56924, 57144, 5879, 5880, 5881, 6093, 6237, 6654, 6655, 9475, 998, hsa-miR-126-3p, hsa-miR-138-5p, hsa-miR-145-5p, hsa-miR-21-5p, hsa-miR-31-5p, 10298, 2263, 3265, 3690, 3696, 3845, 387, 4627, 4628, 4893, 5058, 5062, 5063, 5594, 5604, 5605, 56924, 57144, 5879, 5880, 5881, 6093, 6654, 6655, 79784, 9475, 998, hsa-miR-126-3p, hsa-miR-145-5p, hsa-miR-31-5p, 10298, 3265, 3690, 3845, 387, 4627, 4628, 4893, 5058, 5062, 5063, 5594, 5604, 5605, 56924, 57144, 5879, 5880, 5881, 6093, 6654, 6655, 71, 79784, 9475, 998, hsa-miR-126-3p, hsa-miR-145-5p, hsa-miR-31-5p, 10298, 387, 4627, 4628, 5058, 5062, 5063, 5594, 5604, 5605, 56924, 57144, 5879, 5880, 5881, 60, 6093, 71, 79784, 9475, 998, hsa-miR-31-5p, 55845, 5594, 5604, 5605, 5879, 5880, 5881, 60, 71, 8936, 998, hsa-miR-31-5p, hsa-miR-335-5p, 10163, 55845, 5879, 5880, 5881, 60, 71, 8936, 998, hsa-miR-31-5p, hsa-miR-335-5p, 10163, 55845, 60, 71, 7454, 8936, 8976, 998, hsa-miR-31-5p, hsa-miR-335-5p, 10163, 55845, 60, 71, 7454, 81873, 8936, 8976, hsa-miR-335-5p, 10093, 10163, 55845, 60, 71, 7454, 8936, 8976, 10094, 10163, 55845, 60, 71, 7454, 8936, 8976, 10095, 10163, 55845, 60, 71, 7454, 8936, 8976, 10109, 10163, 55845, 60, 71, 7454, 8936, 8976, 10163, 10552, 55845, 60, 71, 7454, 8936, 8976 |
| Herpes simplex infection | 71.9 | 4049, 8740, 8764, 7188, 8764, hsa-miR-502-3p, 7185, 7186, 7187, 7188, 8764, 10454, 23118, 5601, 6885, 7185, 7186, 7187, 7188, hsa-miR-106b-5p, hsa-miR-17-5p, hsa-miR-20a-5p, hsa-miR-93-5p, 10454, 23118, 3551, 5599, 5601, 5602, 6885, 7185, 7186, 7187, 7188, 7874, hsa-miR-17-5p, 10454, 23118, 3551, 5599, 5601, 5602, 6885, 7874, hsa-miR-146a-5p, hsa-miR-155-5p, hsa-miR-17-5p, 10454, 148022, 23118, 3551, 5599, 5601, 5602, 6885, 7874, hsa-miR-146a-5p, hsa-miR-155-5p, 10454, 148022, 23118, 3551, 6885, 7189, 7874, hsa-miR-146a-5p, hsa-miR-155-5p, 1017, 10454, 148022, 23118, 3551, 6885, 7189, 7874, hsa-miR-146a-5p, 1017, 10454, 23118, 6885, 7189, hsa-miR-10a-5p, 1017, 1457, 1459, 1460, hsa-miR-10a-5p, 1457, 1459, 1460, 708, hsa-miR-10a-5p, 6426, 6732, 708, hsa-miR-10a-5p, hsa-miR-10b-5p, hsa-miR-140-3p, hsa-miR-7-5p, 10929, 6732, 708, 6427, 6732, 708, 6428, 6732, 708, 6429, 6732, 708, 6430, 6732, 708, 6431, 6732, 708, 6432, 6732, 708, 6732, 708, 8683 |
| Insulin signaling pathway | 71.3 | 8835, hsa-miR-194-5p, 122809, 3643, 5770, 5792, 8651, 8835, 9021, 10000, 122809, 207, 208, 3643, 5770, 5792, 10000, 207, 208, 3643, 3667, 5770, 5792, 8471, 8660, hsa-miR-126-3p, hsa-miR-145-5p, hsa-miR-7-5p, 10000, 207, 208, 3643, 3667, 5792, 6654, 6655, 8471, 8660, hsa-miR-126-3p, hsa-miR-145-5p, hsa-miR-7-5p, 10000, 207, 208, 3643, 3667, 5584, 5590, 6654, 6655, 8471, 8660, hsa-miR-126-3p, hsa-miR-145-5p, hsa-miR-7-5p, 10000, 207, 208, 2475, 3667, 5170, 5584, 5590, 6654, 6655, 8471, 8660, hsa-miR-126-3p, hsa-miR-145-5p, hsa-miR-7-5p, 10000, 207, 208, 23533, 2475, 3636, 3667, 5170, 51763, 5290, 5291, 5293, 5294, 5295, 5296, 5584, 5590, 8471, 8503, 8660, hsa-miR-126-3p, 10000, 207, 208, 23533, 2475, 3636, 5170, 51763, 5290, 5291, 5293, 5294, 5295, 5296, 5584, 5590, 6009, 8503, hsa-miR-199a-3p, hsa-miR-373-3p, hsa-miR-520c-3p, hsa-miR-99a-5p, hsa-miR-99b-5p, 10000, 207, 208, 6009, 7248, 7249, 10000, 207, 208, 572, hsa-miR-127-3p, 10000, 207, 208, 2932, 5499, 5500, 5501, 5506, 5507, 5509, 79660, 2932, 2997, 5499, 5500, 5501, 5506, 5507, 5509, 79660, 2932, 2998, 5499, 5500, 5501, 5506, 5507, 5509, 79660, 5499, 5500, 5501, 5506, 5507, 5509, 79660, 808, hsa-miR-1, 163688, 51806, 5255, 5256, 5257, 5260, 5261, 5499, 5500, 5501, 5506, 5507, 5509, 79660, 801, 805, 808, 810, 163688, 51806, 5255, 5256, 5257, 5260, 5261, 5834, 801, 805, 808, 810, 163688, 51806, 5255, 5256, 5257, 5260, 5261, 5836, 801, 805, 808, 810, 163688, 51806, 5255, 5256, 5257, 5260, 5261, 5837, 801, 805, 808, 810 |
| Dopaminergic synapse | 70.7 | 163688, 51806, 5530, 5532, 5533, 801, 805, 808, 810, 5530, 5532, 5533, 808, hsa-miR-1, 5499, 5500, 5501, 5530, 5532, 5533, 5566, 5567, 5568, 5613, 84152, hsa-miR-1, 2931, 2932, 5499, 5500, 5501, 5566, 5567, 5568, 5613, 84152, hsa-miR-1, 10000, 1432, 207, 208, 2931, 2932, 5566, 5567, 5568, 5613, hsa-miR-1, 10000, 1432, 207, 208, 23236, 5330, 5331, 5332, 5566, 5567, 5568, 5613, hsa-miR-1, hsa-miR-122-5p, hsa-miR-222-3p, 1432, 23236, 5330, 5331, 5332, 5566, 5567, 5568, 5600, 5613, hsa-miR-122-5p, hsa-miR-222-3p, 1432, 23236, 5330, 5331, 5332, 5566, 5567, 5568, 5599, 5600, 5601, 5602, 5603, 5613, 6300, hsa-miR-222-3p, 5566, 5567, 5568, 5601, 5613, hsa-miR-106b-5p, hsa-miR-17-5p, hsa-miR-20a-5p, hsa-miR-93-5p, 10488, 5566, 5567, 5568, 5613, 1386, 5566, 5567, 5568, 5613, 1388, 5566, 5567, 5568, 5613, 148327, 5566, 5567, 5568, 5613, 468, 5566, 5567, 5568, 5613, 5566, 5567, 5568, 5613, 64764, 5566, 5567, 5568, 5613, 84699, 5566, 5567, 5568, 5613, 90993, 5566, 5567, 5568, 5613, 9586 |
| Prostate cancer | 70.3 | 10000, 1017, 1027, 1870, 207, 208, 3479, 4790, 5728, 5970, 898, 9134, hsa-let-7a-5p, hsa-let-7b-5p, hsa-miR-103a-3p, hsa-miR-106a-5p, hsa-miR-106b-5p, hsa-miR-125a-5p, hsa-miR-125b-5p, hsa-miR-128, hsa-miR-132-3p, hsa-miR-143-3p, hsa-miR-145-5p, hsa-miR-146a-5p, hsa-miR-15a-5p, hsa-miR-16-5p, hsa-miR-17-5p, hsa-miR-181a-5p, hsa-miR-181c-5p, hsa-miR-181d, hsa-miR-192-5p, hsa-miR-194-5p, hsa-miR-195-5p, hsa-miR-203a, hsa-miR-20a-5p, hsa-miR-21-5p, hsa-miR-217, hsa-miR-221-3p, hsa-miR-223-3p, hsa-miR-26a-5p, hsa-miR-28-5p, hsa-miR-29a-3p, hsa-miR-29b-3p, hsa-miR-29c-3p, hsa-miR-34a-5p, hsa-miR-34b-5p, hsa-miR-34c-5p, hsa-miR-365a-3p, hsa-miR-424-5p, hsa-miR-449a, hsa-miR-503-5p, hsa-miR-519d, hsa-miR-93-5p, hsa-miR-98-5p, 10000, 1017, 1027, 1870, 207, 208, 3479, 5728, 5970, 898, 9134, hsa-let-7a-5p, hsa-let-7b-5p, hsa-miR-103a-3p, hsa-miR-106a-5p, hsa-miR-106b-5p, hsa-miR-125a-5p, hsa-miR-125b-5p, hsa-miR-126-3p, hsa-miR-128, hsa-miR-132-3p, hsa-miR-143-3p, hsa-miR-145-5p, hsa-miR-146a-5p, hsa-miR-16-5p, hsa-miR-17-5p, hsa-miR-181a-5p, hsa-miR-181c-5p, hsa-miR-181d, hsa-miR-192-5p, hsa-miR-194-5p, hsa-miR-195-5p, hsa-miR-203a, hsa-miR-20a-5p, hsa-miR-21-5p, hsa-miR-217, hsa-miR-221-3p, hsa-miR-223-3p, hsa-miR-26a-5p, hsa-miR-28-5p, hsa-miR-29a-3p, hsa-miR-29b-3p, hsa-miR-29c-3p, hsa-miR-34a-5p, hsa-miR-34b-5p, hsa-miR-34c-5p, hsa-miR-424-5p, hsa-miR-449a, hsa-miR-503-5p, hsa-miR-519d, hsa-miR-93-5p, hsa-miR-98-5p, 10000, 1017, 1027, 1870, 1950, 207, 208, 2260, 3479, 5154, 5155, 56034, 5728, 5970, 7039, 80310, 898, 9134, hsa-let-7a-5p, hsa-let-7b-5p, hsa-miR-103a-3p, hsa-miR-106a-5p, hsa-miR-106b-5p, hsa-miR-125a-5p, hsa-miR-125b-5p, hsa-miR-126-3p, hsa-miR-128, hsa-miR-132-3p, hsa-miR-143-3p, hsa-miR-145-5p, hsa-miR-146a-5p, hsa-miR-16-5p, hsa-miR-17-5p, hsa-miR-181a-5p, hsa-miR-181c-5p, hsa-miR-181d, hsa-miR-192-5p, hsa-miR-194-5p, hsa-miR-195-5p, hsa-miR-203a, hsa-miR-20a-5p, hsa-miR-21-5p, hsa-miR-217, hsa-miR-221-3p, hsa-miR-223-3p, hsa-miR-26a-5p, hsa-miR-28-5p, hsa-miR-29a-3p, hsa-miR-29b-3p, hsa-miR-29c-3p, hsa-miR-34a-5p, hsa-miR-34c-5p, hsa-miR-424-5p, hsa-miR-519d, hsa-miR-93-5p, hsa-miR-98-5p, 10000, 1017, 1027, 1870, 1950, 1956, 2064, 207, 208, 2260, 2263, 3479, 3480, 3645, 5154, 5155, 5156, 5159, 56034, 5728, 5970, 7039, 80310, 898, 9134, hsa-let-7a-5p, hsa-let-7b-5p, hsa-miR-103a-3p, hsa-miR-106a-5p, hsa-miR-106b-5p, hsa-miR-125a-5p, hsa-miR-125b-5p, hsa-miR-126-3p, hsa-miR-128, hsa-miR-132-3p, hsa-miR-143-3p, hsa-miR-145-5p, hsa-miR-146a-5p, hsa-miR-17-5p, hsa-miR-181a-5p, hsa-miR-181c-5p, hsa-miR-181d, hsa-miR-192-5p, hsa-miR-194-5p, hsa-miR-195-5p, hsa-miR-203a, hsa-miR-20a-5p, hsa-miR-21-5p, hsa-miR-217, hsa-miR-221-3p, hsa-miR-223-3p, hsa-miR-26a-5p, hsa-miR-28-5p, hsa-miR-29a-3p, hsa-miR-29b-3p, hsa-miR-29c-3p, hsa-miR-335-5p, hsa-miR-34a-5p, hsa-miR-34c-5p, hsa-miR-424-5p, hsa-miR-519d, hsa-miR-93-5p, hsa-miR-98-5p, 1017, 1027, 1950, 1956, 2064, 2260, 2263, 3479, 3480, 3645, 5154, 5155, 5156, 5159, 56034, 5970, 7039, 80310, 898, 9134, hsa-let-7a-5p, hsa-let-7b-5p, hsa-miR-103a-3p, hsa-miR-126-3p, hsa-miR-128, hsa-miR-143-3p, hsa-miR-145-5p, hsa-miR-146a-5p, hsa-miR-181a-5p, hsa-miR-181c-5p, hsa-miR-181d, hsa-miR-195-5p, hsa-miR-21-5p, hsa-miR-217, hsa-miR-28-5p, hsa-miR-335-5p, hsa-miR-34a-5p, hsa-miR-424-5p, hsa-miR-7-5p, 1017, 1027, 1956, 2064, 2260, 2263, 3480, 3645, 5156, 5159, 5970, 6654, 6655, 898, 9134, hsa-let-7a-5p, hsa-let-7b-5p, hsa-miR-103a-3p, hsa-miR-126-3p, hsa-miR-143-3p, hsa-miR-145-5p, hsa-miR-181a-5p, hsa-miR-181c-5p, hsa-miR-181d, hsa-miR-195-5p, hsa-miR-217, hsa-miR-28-5p, hsa-miR-335-5p, hsa-miR-34a-5p, hsa-miR-424-5p, hsa-miR-7-5p, 1017, 1027, 5970, 6654, 6655, 898, 9134, hsa-let-7a-5p, hsa-let-7b-5p, hsa-miR-103a-3p, hsa-miR-124-3p, hsa-miR-126-3p, hsa-miR-143-3p, hsa-miR-145-5p, hsa-miR-181a-5p, hsa-miR-181c-5p, hsa-miR-181d, hsa-miR-195-5p, hsa-miR-217, hsa-miR-28-5p, hsa-miR-335-5p, hsa-miR-34a-5p, hsa-miR-424-5p, hsa-miR-7-5p, 3845, 6654, 6655, hsa-let-7a-5p, hsa-let-7b-5p, hsa-miR-124-3p, hsa-miR-126-3p, hsa-miR-143-3p, hsa-miR-145-5p, hsa-miR-181a-5p, hsa-miR-181c-5p, hsa-miR-181d, hsa-miR-195-5p, hsa-miR-217, hsa-miR-28-5p, hsa-miR-335-5p, hsa-miR-34a-5p, hsa-miR-424-5p, hsa-miR-7-5p, 3265, 3845, 6654, 6655, hsa-let-7a-5p, hsa-let-7b-5p, hsa-miR-124-3p, hsa-miR-143-3p, hsa-miR-145-5p, hsa-miR-181a-5p, hsa-miR-181d, hsa-miR-195-5p, hsa-miR-28-5p, hsa-miR-335-5p, hsa-miR-34a-5p, hsa-miR-424-5p, hsa-miR-7-5p, 3265, 3845, 4893, 6654, 6655, hsa-let-7a-5p, hsa-let-7b-5p, hsa-miR-124-3p, hsa-miR-145-5p, hsa-miR-195-5p, hsa-miR-28-5p, hsa-miR-335-5p, hsa-miR-34a-5p, hsa-miR-424-5p, hsa-miR-7-5p, 3265, 3845, 4893, 5894, hsa-miR-124-3p, hsa-miR-195-5p, hsa-miR-28-5p, hsa-miR-335-5p, hsa-miR-34a-5p, hsa-miR-424-5p, hsa-miR-7-5p, 3265, 369, 3845, 4893, 5894, 673, hsa-miR-124-3p, hsa-miR-28-5p, hsa-miR-335-5p, hsa-miR-34a-5p, hsa-miR-424-5p, 369, 5604, 5894, 673, hsa-miR-124-3p, hsa-miR-28-5p, hsa-miR-335-5p, hsa-miR-34a-5p, hsa-miR-424-5p, 369, 5604, 5605, 5894, 673, hsa-miR-124-3p, hsa-miR-28-5p, hsa-miR-335-5p, 5594, 5604, 5605, hsa-miR-124-3p, hsa-miR-28-5p, hsa-miR-335-5p, 5594, 5595, 5604, 5605, hsa-miR-124-3p, 3320, 3326, 367, 5594, 5595, 7184, hsa-miR-124-3p, hsa-miR-488-5p, 354, 367 |

**Table S7. Re-analysis of the meta-pathway.** The first path has been considered to compose the network in Supplementary Figure 3.

| **Max Score** | **Involved Genes and miRNAs** |
| --- | --- |
| 238.42 | 10451, 1794, 387, 50649, 5879, 5880, 7074, 7409, 7410, 8874, 9459, hsa-miR-122-5p, 10451, 387, 50649, 5879, 5880, 5881, 7074, 7409, 7410, 8874, 9459, hsa-miR-122-5p, 10451, 387, 5879, 5880, 5881, 6850, 7074, 7409, 7410, 8874, 9459, hsa-miR-122-5p, hsa-miR-21-5p, hsa-miR-31-5p, 387, 5879, 5880, 5881, 6850, 8874, 9459, 998, hsa-miR-122-5p, hsa-miR-155-5p, hsa-miR-21-5p, hsa-miR-29a-3p, hsa-miR-29b-3p, hsa-miR-29c-3p, hsa-miR-31-5p, 3265, 3845, 387, 4893, 5879, 5880, 5881, 6654, 6655, 6850, 8874, 9459, 998, hsa-miR-122-5p, hsa-miR-145-5p, hsa-miR-155-5p, hsa-miR-21-5p, hsa-miR-29a-3p, hsa-miR-29b-3p, hsa-miR-29c-3p, 3265, 3845, 387, 4893, 5594, 5879, 5880, 5894, 6654, 6655, 6850, 8611, 8612, 8613, 998, hsa-miR-122-5p, hsa-miR-126-3p, hsa-miR-145-5p, hsa-miR-155-5p, hsa-miR-21-5p, hsa-miR-29a-3p, hsa-miR-29b-3p, hsa-miR-29c-3p, hsa-miR-7-5p, 3265, 382, 3845, 4893, 5337, 5338, 5594, 5595, 5879, 5880, 5894, 6654, 6655, 6850, 8611, 8612, 8613, 998, hsa-miR-122-5p, hsa-miR-126-3p, hsa-miR-145-5p, hsa-miR-155-5p, hsa-miR-21-5p, hsa-miR-29a-3p, hsa-miR-29b-3p, hsa-miR-29c-3p, hsa-miR-34a-5p, hsa-miR-424-5p, hsa-miR-7-5p, 3265, 382, 3845, 4893, 5337, 5338, 5594, 5595, 5894, 6654, 6655, 6850, 8611, 8612, 8613, hsa-let-7a-5p, hsa-miR-122-5p, hsa-miR-126-3p, hsa-miR-145-5p, hsa-miR-155-5p, hsa-miR-21-5p, hsa-miR-29a-3p, hsa-miR-29b-3p, hsa-miR-29c-3p, hsa-miR-34a-5p, hsa-miR-424-5p, hsa-miR-7-5p, 10000, 1950, 207, 208, 3265, 3479, 382, 3845, 5154, 5155, 5337, 5338, 5594, 5595, 56034, 5728, 5894, 6654, 6655, 6850, 7039, 80310, 8611, 8612, 8613, hsa-let-7a-5p, hsa-miR-122-5p, hsa-miR-126-3p, hsa-miR-145-5p, hsa-miR-155-5p, hsa-miR-21-5p, hsa-miR-29a-3p, hsa-miR-29b-3p, hsa-miR-29c-3p, hsa-miR-34a-5p, hsa-miR-424-5p, hsa-miR-519d, hsa-miR-7-5p, 10000, 10454, 1950, 207, 208, 23118, 3265, 3479, 3845, 5154, 5155, 5594, 5595, 56034, 5728, 5894, 6654, 6655, 6885, 7039, 7189, 80310, hsa-let-7a-5p, hsa-miR-103a-3p, hsa-miR-122-5p, hsa-miR-124-3p, hsa-miR-126-3p, hsa-miR-145-5p, hsa-miR-146a-5p, hsa-miR-155-5p, hsa-miR-21-5p, hsa-miR-217, hsa-miR-221-3p, hsa-miR-26a-5p, hsa-miR-29a-3p, hsa-miR-34a-5p, hsa-miR-34b-5p, hsa-miR-424-5p, hsa-miR-519d, hsa-miR-7-5p, 10000, 10454, 1950, 207, 208, 23118, 3479, 5154, 5155, 5566, 5567, 5568, 5594, 5595, 56034, 5613, 5728, 5894, 6654, 6655, 6885, 7039, 7189, 80310, hsa-let-7a-5p, hsa-miR-103a-3p, hsa-miR-122-5p, hsa-miR-124-3p, hsa-miR-146a-5p, hsa-miR-155-5p, hsa-miR-21-5p, hsa-miR-217, hsa-miR-221-3p, hsa-miR-26a-5p, hsa-miR-29a-3p, hsa-miR-34b-5p, hsa-miR-519d, 10000, 1950, 207, 208, 3479, 5154, 5155, 5566, 5567, 5568, 56034, 5613, 6654, 6655, 7039, 80310, hsa-miR-125b-5p, 10000, 163688, 207, 208, 51806, 5566, 5567, 5568, 5613, 801, 805, 808, 810, 84152, hsa-miR-125b-5p, 2903, 5566, 5567, 5568, 5613, hsa-miR-125b-5p, 5566, 5567, 5568, 5613, 775, hsa-miR-133a, 10368, 5566, 5567, 5568, 5613, 10369, 5566, 5567, 5568, 5613, 10488, 5566, 5567, 5568, 5613, 116443, 5566, 5567, 5568, 5613, 116444, 5566, 5567, 5568, 5613, 1386, 5566, 5567, 5568, 5613, 1388, 5566, 5567, 5568, 5613, 148327, 5566, 5567, 5568, 5613, 27091, 5566, 5567, 5568, 5613, 27092, 5566, 5567, 5568, 5613, 2890, 5566, 5567, 5568, 5613, 2892, 5566, 5567, 5568, 5613, 2893, 5566, 5567, 5568, 5613, 2902, 5566, 5567, 5568, 5613, 2904, 5566, 5567, 5568, 5613, 2905, 5566, 5567, 5568, 5613, 2906, 5566, 5567, 5568, 5613, 468, 5566, 5567, 5568, 5613, 5350, 5566, 5567, 5568, 5613, 5566, 5567, 5568, 55799, 5613, 5566, 5567, 5568, 5613, 59283, 5566, 5567, 5568, 5613, 59284, 5566, 5567, 5568, 5613, 59285, 5566, 5567, 5568, 5613, 6262, 5566, 5567, 5568, 5613, 64764, 5566, 5567, 5568, 5613, 776, 5566, 5567, 5568, 5613, 778, 5566, 5567, 5568, 5613, 779, 5566, 5567, 5568, 5613, 781, 5566, 5567, 5568, 5613, 782, 5566, 5567, 5568, 5613, 783, 5566, 5567, 5568, 5613, 784, 5566, 5567, 5568, 5613, 785, 5566, 5567, 5568, 5613, 786, 5566, 5567, 5568, 5613, 84699, 5566, 5567, 5568, 5613, 90993, 5566, 5567, 5568, 5613, 9254, 5566, 5567, 5568, 5613, 93589, 5566, 5567, 5568, 5613, 9586 |
| 129.87 | 10451, 1794, 387, 50649, 5879, 5880, 7074, 7409, 7410, 8874, 9459, hsa-miR-122-5p, 10451, 387, 50649, 5879, 5880, 5881, 7074, 7409, 7410, 8874, 9459, hsa-miR-122-5p, 10451, 387, 5879, 5880, 5881, 6850, 7074, 7409, 7410, 8874, 9459, hsa-miR-122-5p, hsa-miR-21-5p, hsa-miR-31-5p, 387, 5879, 5880, 5881, 6850, 8874, 9459, 998, hsa-miR-122-5p, hsa-miR-155-5p, hsa-miR-21-5p, hsa-miR-29a-3p, hsa-miR-29b-3p, hsa-miR-29c-3p, hsa-miR-31-5p, 3265, 3845, 387, 4893, 5879, 5880, 5881, 6654, 6655, 6850, 8874, 9459, 998, hsa-miR-122-5p, hsa-miR-145-5p, hsa-miR-155-5p, hsa-miR-21-5p, hsa-miR-29a-3p, hsa-miR-29b-3p, hsa-miR-29c-3p, 3265, 3845, 387, 4893, 5594, 5879, 5880, 5894, 6654, 6655, 6850, 8611, 8612, 8613, 998, hsa-miR-122-5p, hsa-miR-126-3p, hsa-miR-145-5p, hsa-miR-155-5p, hsa-miR-21-5p, hsa-miR-29a-3p, hsa-miR-29b-3p, hsa-miR-29c-3p, hsa-miR-7-5p, 3265, 382, 3845, 4893, 5337, 5338, 5594, 5595, 5879, 5880, 5894, 6654, 6655, 6850, 8611, 8612, 8613, 998, hsa-miR-122-5p, hsa-miR-126-3p, hsa-miR-145-5p, hsa-miR-155-5p, hsa-miR-21-5p, hsa-miR-29a-3p, hsa-miR-29b-3p, hsa-miR-29c-3p, hsa-miR-34a-5p, hsa-miR-424-5p, hsa-miR-7-5p, 3265, 382, 3845, 4893, 5337, 5338, 5594, 5595, 5894, 6654, 6655, 6850, 8611, 8612, 8613, hsa-let-7a-5p, hsa-miR-122-5p, hsa-miR-126-3p, hsa-miR-145-5p, hsa-miR-155-5p, hsa-miR-21-5p, hsa-miR-29a-3p, hsa-miR-29b-3p, hsa-miR-29c-3p, hsa-miR-34a-5p, hsa-miR-424-5p, hsa-miR-7-5p, 10000, 1950, 207, 208, 3265, 3479, 382, 3845, 5154, 5155, 5337, 5338, 5594, 5595, 56034, 5728, 5894, 6654, 6655, 6850, 7039, 80310, 8611, 8612, 8613, hsa-let-7a-5p, hsa-miR-122-5p, hsa-miR-126-3p, hsa-miR-145-5p, hsa-miR-155-5p, hsa-miR-21-5p, hsa-miR-29a-3p, hsa-miR-29b-3p, hsa-miR-29c-3p, hsa-miR-34a-5p, hsa-miR-424-5p, hsa-miR-519d, hsa-miR-7-5p, 10000, 10454, 1950, 207, 208, 23118, 3265, 3479, 3845, 5154, 5155, 5594, 5595, 56034, 5728, 5894, 6654, 6655, 6885, 7039, 7189, 80310, hsa-let-7a-5p, hsa-miR-103a-3p, hsa-miR-122-5p, hsa-miR-124-3p, hsa-miR-126-3p, hsa-miR-145-5p, hsa-miR-146a-5p, hsa-miR-155-5p, hsa-miR-21-5p, hsa-miR-217, hsa-miR-221-3p, hsa-miR-26a-5p, hsa-miR-29a-3p, hsa-miR-34a-5p, hsa-miR-34b-5p, hsa-miR-424-5p, hsa-miR-519d, hsa-miR-7-5p, 10000, 10454, 1950, 207, 208, 23118, 3479, 5154, 5155, 5566, 5567, 5568, 5594, 5595, 56034, 5613, 5728, 5894, 6654, 6655, 6885, 7039, 7189, 80310, hsa-let-7a-5p, hsa-miR-103a-3p, hsa-miR-122-5p, hsa-miR-124-3p, hsa-miR-146a-5p, hsa-miR-155-5p, hsa-miR-21-5p, hsa-miR-217, hsa-miR-221-3p, hsa-miR-26a-5p, hsa-miR-29a-3p, hsa-miR-34b-5p, hsa-miR-519d, 10454, 23118, 5566, 5567, 5568, 5594, 5595, 5599, 5601, 5602, 5613, 5728, 5894, 6885, 7189, hsa-let-7a-5p, hsa-miR-103a-3p, hsa-miR-122-5p, hsa-miR-124-3p, hsa-miR-146a-5p, hsa-miR-155-5p, hsa-miR-17-5p, hsa-miR-21-5p, hsa-miR-217, hsa-miR-221-3p, hsa-miR-26a-5p, hsa-miR-29a-3p, hsa-miR-34b-5p, hsa-miR-519d, 2353, 3725, 5566, 5567, 5568, 5594, 5595, 5599, 5601, 5602, 5613, 5894, 7189, 8767, hsa-let-7a-5p, hsa-miR-103a-3p, hsa-miR-122-5p, hsa-miR-124-3p, hsa-miR-146a-5p, hsa-miR-155-5p, hsa-miR-17-5p, hsa-miR-21-5p, hsa-miR-221-3p, hsa-miR-34b-5p, 2353, 3725, 5566, 5567, 5568, 5613, 5894, 7189, 8767, hsa-let-7a-5p, hsa-miR-103a-3p, hsa-miR-124-3p, hsa-miR-146a-5p, hsa-miR-146b-5p, hsa-miR-17-5p, hsa-miR-21-5p, hsa-miR-221-3p, hsa-miR-34b-5p, 107, 108, 109, 111, 112, 113, 114, 115, 196883, 5566, 5567, 5568, 5613, hsa-miR-103a-3p, hsa-miR-146b-5p, hsa-miR-34b-5p, 107, 108, 109, 111, 112, 113, 114, 115, 196883, 2778, hsa-miR-103a-3p, hsa-miR-34b-5p, 112, 1385, 2778, 4261, hsa-miR-103a-3p, hsa-miR-182-5p, hsa-miR-34b-5p, 1385, 4261, hsa-miR-103a-3p, hsa-miR-182-5p, hsa-miR-203a, hsa-miR-34b-5p, 1385, 4261, 4800, 4801, 4802, 5993, 5994, 8625, 1385, 3108, 4800, 4801, 4802, 5993, 5994, 8625, hsa-miR-638, 1385, 3109, 4800, 4801, 4802, 5993, 5994, 8625, 1385, 3111, 4800, 4801, 4802, 5993, 5994, 8625, 1385, 3112, 4800, 4801, 4802, 5993, 5994, 8625, 1385, 3113, 4800, 4801, 4802, 5993, 5994, 8625, 1385, 3115, 4800, 4801, 4802, 5993, 5994, 8625, 1385, 3117, 4800, 4801, 4802, 5993, 5994, 8625, 1385, 3118, 4800, 4801, 4802, 5993, 5994, 8625, 1385, 3119, 4800, 4801, 4802, 5993, 5994, 8625, 1385, 3122, 4800, 4801, 4802, 5993, 5994, 8625, 1385, 3123, 4800, 4801, 4802, 5993, 5994, 8625, 1385, 3127, 4800, 4801, 4802, 5993, 5994, 8625, 1385, 4800, 4801, 4802, 5993, 5994, 8625, 972 |
| 81.79 | 10451, 1794, 387, 50649, 5879, 5880, 7074, 7409, 7410, 8874, 9459, hsa-miR-122-5p, 10451, 387, 50649, 5879, 5880, 5881, 7074, 7409, 7410, 8874, 9459, hsa-miR-122-5p, 10451, 387, 5879, 5880, 5881, 6850, 7074, 7409, 7410, 8874, 9459, hsa-miR-122-5p, hsa-miR-21-5p, hsa-miR-31-5p, 387, 5879, 5880, 5881, 6850, 8874, 9459, 998, hsa-miR-122-5p, hsa-miR-155-5p, hsa-miR-21-5p, hsa-miR-29a-3p, hsa-miR-29b-3p, hsa-miR-29c-3p, hsa-miR-31-5p, 3265, 3845, 387, 4893, 5879, 5880, 5881, 6654, 6655, 6850, 8874, 9459, 998, hsa-miR-122-5p, hsa-miR-145-5p, hsa-miR-155-5p, hsa-miR-21-5p, hsa-miR-29a-3p, hsa-miR-29b-3p, hsa-miR-29c-3p, 3265, 3845, 387, 4893, 5594, 5879, 5880, 5894, 6654, 6655, 6850, 8611, 8612, 8613, 998, hsa-miR-122-5p, hsa-miR-126-3p, hsa-miR-145-5p, hsa-miR-155-5p, hsa-miR-21-5p, hsa-miR-29a-3p, hsa-miR-29b-3p, hsa-miR-29c-3p, hsa-miR-7-5p, 3265, 382, 3845, 4893, 5337, 5338, 5594, 5595, 5879, 5880, 5894, 6654, 6655, 6850, 8611, 8612, 8613, 998, hsa-miR-122-5p, hsa-miR-126-3p, hsa-miR-145-5p, hsa-miR-155-5p, hsa-miR-21-5p, hsa-miR-29a-3p, hsa-miR-29b-3p, hsa-miR-29c-3p, hsa-miR-34a-5p, hsa-miR-424-5p, hsa-miR-7-5p, 3265, 382, 3845, 4893, 5337, 5338, 5594, 5595, 5894, 6654, 6655, 6850, 8611, 8612, 8613, hsa-let-7a-5p, hsa-miR-122-5p, hsa-miR-126-3p, hsa-miR-145-5p, hsa-miR-155-5p, hsa-miR-21-5p, hsa-miR-29a-3p, hsa-miR-29b-3p, hsa-miR-29c-3p, hsa-miR-34a-5p, hsa-miR-424-5p, hsa-miR-7-5p, 10000, 1950, 207, 208, 3265, 3479, 382, 3845, 5154, 5155, 5337, 5338, 5594, 5595, 56034, 5728, 5894, 6654, 6655, 6850, 7039, 80310, 8611, 8612, 8613, hsa-let-7a-5p, hsa-miR-122-5p, hsa-miR-126-3p, hsa-miR-145-5p, hsa-miR-155-5p, hsa-miR-21-5p, hsa-miR-29a-3p, hsa-miR-29b-3p, hsa-miR-29c-3p, hsa-miR-34a-5p, hsa-miR-424-5p, hsa-miR-519d, hsa-miR-7-5p, 10000, 10454, 1950, 207, 208, 23118, 3265, 3479, 3845, 5154, 5155, 5594, 5595, 56034, 5728, 5894, 6654, 6655, 6885, 7039, 7189, 80310, hsa-let-7a-5p, hsa-miR-103a-3p, hsa-miR-122-5p, hsa-miR-124-3p, hsa-miR-126-3p, hsa-miR-145-5p, hsa-miR-146a-5p, hsa-miR-155-5p, hsa-miR-21-5p, hsa-miR-217, hsa-miR-221-3p, hsa-miR-26a-5p, hsa-miR-29a-3p, hsa-miR-34a-5p, hsa-miR-34b-5p, hsa-miR-424-5p, hsa-miR-519d, hsa-miR-7-5p, 10000, 10454, 1950, 207, 208, 23118, 3479, 5154, 5155, 5566, 5567, 5568, 5594, 5595, 56034, 5613, 5728, 5894, 6654, 6655, 6885, 7039, 7189, 80310, hsa-let-7a-5p, hsa-miR-103a-3p, hsa-miR-122-5p, hsa-miR-124-3p, hsa-miR-146a-5p, hsa-miR-155-5p, hsa-miR-21-5p, hsa-miR-217, hsa-miR-221-3p, hsa-miR-26a-5p, hsa-miR-29a-3p, hsa-miR-34b-5p, hsa-miR-519d, 10454, 23118, 5566, 5567, 5568, 5594, 5595, 5599, 5601, 5602, 5613, 5728, 5894, 6885, 7189, hsa-let-7a-5p, hsa-miR-103a-3p, hsa-miR-122-5p, hsa-miR-124-3p, hsa-miR-146a-5p, hsa-miR-155-5p, hsa-miR-17-5p, hsa-miR-21-5p, hsa-miR-217, hsa-miR-221-3p, hsa-miR-26a-5p, hsa-miR-29a-3p, hsa-miR-34b-5p, hsa-miR-519d, 2353, 3725, 5566, 5567, 5568, 5594, 5595, 5599, 5601, 5602, 5613, 5894, 7189, 8767, hsa-let-7a-5p, hsa-miR-103a-3p, hsa-miR-122-5p, hsa-miR-124-3p, hsa-miR-146a-5p, hsa-miR-155-5p, hsa-miR-17-5p, hsa-miR-21-5p, hsa-miR-221-3p, hsa-miR-34b-5p, 2353, 3725, 5566, 5567, 5568, 5613, 5894, 7189, 8767, hsa-let-7a-5p, hsa-miR-103a-3p, hsa-miR-124-3p, hsa-miR-146a-5p, hsa-miR-146b-5p, hsa-miR-17-5p, hsa-miR-21-5p, hsa-miR-221-3p, hsa-miR-34b-5p, 107, 108, 109, 111, 112, 113, 114, 115, 196883, 5566, 5567, 5568, 5613, hsa-miR-103a-3p, hsa-miR-146b-5p, hsa-miR-34b-5p, 107, 108, 109, 111, 112, 113, 114, 115, 196883, 2778, hsa-miR-103a-3p, hsa-miR-34b-5p, 112, 1385, 2778, 4261, hsa-miR-103a-3p, hsa-miR-182-5p, hsa-miR-34b-5p, 1385, 4261, hsa-miR-103a-3p, hsa-miR-182-5p, hsa-miR-203a, hsa-miR-34b-5p, 1385, 4261, 4800, 4801, 4802, 5993, 5994, 8625, 4261, 6772 |
| 74.49 | 10451, 1794, 387, 50649, 5879, 5880, 7074, 7409, 7410, 8874, 9459, hsa-miR-122-5p, 10451, 387, 50649, 5879, 5880, 5881, 7074, 7409, 7410, 8874, 9459, hsa-miR-122-5p, 10451, 387, 5879, 5880, 5881, 6850, 7074, 7409, 7410, 8874, 9459, hsa-miR-122-5p, hsa-miR-21-5p, hsa-miR-31-5p, 387, 5879, 5880, 5881, 6850, 8874, 9459, 998, hsa-miR-122-5p, hsa-miR-155-5p, hsa-miR-21-5p, hsa-miR-29a-3p, hsa-miR-29b-3p, hsa-miR-29c-3p, hsa-miR-31-5p, 3265, 3845, 387, 4893, 5879, 5880, 5881, 6654, 6655, 6850, 8874, 9459, 998, hsa-miR-122-5p, hsa-miR-145-5p, hsa-miR-155-5p, hsa-miR-21-5p, hsa-miR-29a-3p, hsa-miR-29b-3p, hsa-miR-29c-3p, 3265, 3845, 387, 4893, 5594, 5879, 5880, 5894, 6654, 6655, 6850, 8611, 8612, 8613, 998, hsa-miR-122-5p, hsa-miR-126-3p, hsa-miR-145-5p, hsa-miR-155-5p, hsa-miR-21-5p, hsa-miR-29a-3p, hsa-miR-29b-3p, hsa-miR-29c-3p, hsa-miR-7-5p, 3265, 382, 3845, 4893, 5337, 5338, 5594, 5595, 5879, 5880, 5894, 6654, 6655, 6850, 8611, 8612, 8613, 998, hsa-miR-122-5p, hsa-miR-126-3p, hsa-miR-145-5p, hsa-miR-155-5p, hsa-miR-21-5p, hsa-miR-29a-3p, hsa-miR-29b-3p, hsa-miR-29c-3p, hsa-miR-34a-5p, hsa-miR-424-5p, hsa-miR-7-5p, 3265, 382, 3845, 4893, 5337, 5338, 5594, 5595, 5894, 6654, 6655, 6850, 8611, 8612, 8613, hsa-let-7a-5p, hsa-miR-122-5p, hsa-miR-126-3p, hsa-miR-145-5p, hsa-miR-155-5p, hsa-miR-21-5p, hsa-miR-29a-3p, hsa-miR-29b-3p, hsa-miR-29c-3p, hsa-miR-34a-5p, hsa-miR-424-5p, hsa-miR-7-5p, 10000, 1950, 207, 208, 3265, 3479, 382, 3845, 5154, 5155, 5337, 5338, 5594, 5595, 56034, 5728, 5894, 6654, 6655, 6850, 7039, 80310, 8611, 8612, 8613, hsa-let-7a-5p, hsa-miR-122-5p, hsa-miR-126-3p, hsa-miR-145-5p, hsa-miR-155-5p, hsa-miR-21-5p, hsa-miR-29a-3p, hsa-miR-29b-3p, hsa-miR-29c-3p, hsa-miR-34a-5p, hsa-miR-424-5p, hsa-miR-519d, hsa-miR-7-5p, 10000, 10454, 1950, 207, 208, 23118, 3265, 3479, 3845, 5154, 5155, 5594, 5595, 56034, 5728, 5894, 6654, 6655, 6885, 7039, 7189, 80310, hsa-let-7a-5p, hsa-miR-103a-3p, hsa-miR-122-5p, hsa-miR-124-3p, hsa-miR-126-3p, hsa-miR-145-5p, hsa-miR-146a-5p, hsa-miR-155-5p, hsa-miR-21-5p, hsa-miR-217, hsa-miR-221-3p, hsa-miR-26a-5p, hsa-miR-29a-3p, hsa-miR-34a-5p, hsa-miR-34b-5p, hsa-miR-424-5p, hsa-miR-519d, hsa-miR-7-5p, 10000, 10454, 1950, 207, 208, 23118, 3479, 5154, 5155, 5566, 5567, 5568, 5594, 5595, 56034, 5613, 5728, 5894, 6654, 6655, 6885, 7039, 7189, 80310, hsa-let-7a-5p, hsa-miR-103a-3p, hsa-miR-122-5p, hsa-miR-124-3p, hsa-miR-146a-5p, hsa-miR-155-5p, hsa-miR-21-5p, hsa-miR-217, hsa-miR-221-3p, hsa-miR-26a-5p, hsa-miR-29a-3p, hsa-miR-34b-5p, hsa-miR-519d, 10454, 23118, 5566, 5567, 5568, 5594, 5595, 5599, 5601, 5602, 5613, 5728, 5894, 6885, 7189, hsa-let-7a-5p, hsa-miR-103a-3p, hsa-miR-122-5p, hsa-miR-124-3p, hsa-miR-146a-5p, hsa-miR-155-5p, hsa-miR-17-5p, hsa-miR-21-5p, hsa-miR-217, hsa-miR-221-3p, hsa-miR-26a-5p, hsa-miR-29a-3p, hsa-miR-34b-5p, hsa-miR-519d, 2353, 3725, 5566, 5567, 5568, 5594, 5595, 5599, 5601, 5602, 5613, 5894, 7189, 8767, hsa-let-7a-5p, hsa-miR-103a-3p, hsa-miR-122-5p, hsa-miR-124-3p, hsa-miR-146a-5p, hsa-miR-155-5p, hsa-miR-17-5p, hsa-miR-21-5p, hsa-miR-221-3p, hsa-miR-34b-5p, 3320, 3326, 367, 5594, 5595, 7184, hsa-miR-124-3p, hsa-miR-488-5p, 123745, 5594, 5595, 255189, 5594, 5595, 283748, 5594, 5595, 5321, 5594, 5595 |
| 74.15 | 10451, 1794, 387, 50649, 5879, 5880, 7074, 7409, 7410, 8874, 9459, hsa-miR-122-5p, 10451, 387, 50649, 5879, 5880, 5881, 7074, 7409, 7410, 8874, 9459, hsa-miR-122-5p, 10451, 387, 5879, 5880, 5881, 6850, 7074, 7409, 7410, 8874, 9459, hsa-miR-122-5p, hsa-miR-21-5p, hsa-miR-31-5p, 387, 5879, 5880, 5881, 6850, 8874, 9459, 998, hsa-miR-122-5p, hsa-miR-155-5p, hsa-miR-21-5p, hsa-miR-29a-3p, hsa-miR-29b-3p, hsa-miR-29c-3p, hsa-miR-31-5p, 3265, 3845, 387, 4893, 5879, 5880, 5881, 6654, 6655, 6850, 8874, 9459, 998, hsa-miR-122-5p, hsa-miR-145-5p, hsa-miR-155-5p, hsa-miR-21-5p, hsa-miR-29a-3p, hsa-miR-29b-3p, hsa-miR-29c-3p, 3265, 3845, 387, 4893, 5594, 5879, 5880, 5894, 6654, 6655, 6850, 8611, 8612, 8613, 998, hsa-miR-122-5p, hsa-miR-126-3p, hsa-miR-145-5p, hsa-miR-155-5p, hsa-miR-21-5p, hsa-miR-29a-3p, hsa-miR-29b-3p, hsa-miR-29c-3p, hsa-miR-7-5p, 3265, 382, 3845, 4893, 5337, 5338, 5594, 5595, 5879, 5880, 5894, 6654, 6655, 6850, 8611, 8612, 8613, 998, hsa-miR-122-5p, hsa-miR-126-3p, hsa-miR-145-5p, hsa-miR-155-5p, hsa-miR-21-5p, hsa-miR-29a-3p, hsa-miR-29b-3p, hsa-miR-29c-3p, hsa-miR-34a-5p, hsa-miR-424-5p, hsa-miR-7-5p, 3265, 382, 3845, 4893, 5337, 5338, 5594, 5595, 5894, 6654, 6655, 6850, 8611, 8612, 8613, hsa-let-7a-5p, hsa-miR-122-5p, hsa-miR-126-3p, hsa-miR-145-5p, hsa-miR-155-5p, hsa-miR-21-5p, hsa-miR-29a-3p, hsa-miR-29b-3p, hsa-miR-29c-3p, hsa-miR-34a-5p, hsa-miR-424-5p, hsa-miR-7-5p, 10000, 1950, 207, 208, 3265, 3479, 382, 3845, 5154, 5155, 5337, 5338, 5594, 5595, 56034, 5728, 5894, 6654, 6655, 6850, 7039, 80310, 8611, 8612, 8613, hsa-let-7a-5p, hsa-miR-122-5p, hsa-miR-126-3p, hsa-miR-145-5p, hsa-miR-155-5p, hsa-miR-21-5p, hsa-miR-29a-3p, hsa-miR-29b-3p, hsa-miR-29c-3p, hsa-miR-34a-5p, hsa-miR-424-5p, hsa-miR-519d, hsa-miR-7-5p, 10000, 10454, 1950, 207, 208, 23118, 3265, 3479, 3845, 5154, 5155, 5594, 5595, 56034, 5728, 5894, 6654, 6655, 6885, 7039, 7189, 80310, hsa-let-7a-5p, hsa-miR-103a-3p, hsa-miR-122-5p, hsa-miR-124-3p, hsa-miR-126-3p, hsa-miR-145-5p, hsa-miR-146a-5p, hsa-miR-155-5p, hsa-miR-21-5p, hsa-miR-217, hsa-miR-221-3p, hsa-miR-26a-5p, hsa-miR-29a-3p, hsa-miR-34a-5p, hsa-miR-34b-5p, hsa-miR-424-5p, hsa-miR-519d, hsa-miR-7-5p, 10000, 10454, 1950, 207, 208, 23118, 3479, 5154, 5155, 5566, 5567, 5568, 5594, 5595, 56034, 5613, 5728, 5894, 6654, 6655, 6885, 7039, 7189, 80310, hsa-let-7a-5p, hsa-miR-103a-3p, hsa-miR-122-5p, hsa-miR-124-3p, hsa-miR-146a-5p, hsa-miR-155-5p, hsa-miR-21-5p, hsa-miR-217, hsa-miR-221-3p, hsa-miR-26a-5p, hsa-miR-29a-3p, hsa-miR-34b-5p, hsa-miR-519d, 10454, 23118, 5566, 5567, 5568, 5594, 5595, 5599, 5601, 5602, 5613, 5728, 5894, 6885, 7189, hsa-let-7a-5p, hsa-miR-103a-3p, hsa-miR-122-5p, hsa-miR-124-3p, hsa-miR-146a-5p, hsa-miR-155-5p, hsa-miR-17-5p, hsa-miR-21-5p, hsa-miR-217, hsa-miR-221-3p, hsa-miR-26a-5p, hsa-miR-29a-3p, hsa-miR-34b-5p, hsa-miR-519d, 2353, 3725, 5566, 5567, 5568, 5594, 5595, 5599, 5601, 5602, 5613, 5894, 7189, 8767, hsa-let-7a-5p, hsa-miR-103a-3p, hsa-miR-122-5p, hsa-miR-124-3p, hsa-miR-146a-5p, hsa-miR-155-5p, hsa-miR-17-5p, hsa-miR-21-5p, hsa-miR-221-3p, hsa-miR-34b-5p, 2353, 3725, 5566, 5567, 5568, 5613, 5894, 7189, 8767, hsa-let-7a-5p, hsa-miR-103a-3p, hsa-miR-124-3p, hsa-miR-146a-5p, hsa-miR-146b-5p, hsa-miR-17-5p, hsa-miR-21-5p, hsa-miR-221-3p, hsa-miR-34b-5p, 107, 108, 109, 111, 112, 113, 114, 115, 196883, 5566, 5567, 5568, 5613, hsa-miR-103a-3p, hsa-miR-146b-5p, hsa-miR-34b-5p, 107, 108, 109, 111, 112, 113, 114, 115, 196883, 2778, hsa-miR-103a-3p, hsa-miR-34b-5p, 112, 1385, 2778, 4261, hsa-miR-103a-3p, hsa-miR-182-5p, hsa-miR-34b-5p, 112, 2778, hsa-miR-182-5p, hsa-miR-96-5p |
| 71.00 | 10451, 1794, 387, 50649, 5879, 5880, 7074, 7409, 7410, 8874, 9459, hsa-miR-122-5p, 10451, 387, 50649, 5879, 5880, 5881, 7074, 7409, 7410, 8874, 9459, hsa-miR-122-5p, 10451, 387, 5879, 5880, 5881, 6850, 7074, 7409, 7410, 8874, 9459, hsa-miR-122-5p, hsa-miR-21-5p, hsa-miR-31-5p, 387, 5879, 5880, 5881, 6850, 8874, 9459, 998, hsa-miR-122-5p, hsa-miR-155-5p, hsa-miR-21-5p, hsa-miR-29a-3p, hsa-miR-29b-3p, hsa-miR-29c-3p, hsa-miR-31-5p, 3265, 3845, 387, 4893, 5879, 5880, 5881, 6654, 6655, 6850, 8874, 9459, 998, hsa-miR-122-5p, hsa-miR-145-5p, hsa-miR-155-5p, hsa-miR-21-5p, hsa-miR-29a-3p, hsa-miR-29b-3p, hsa-miR-29c-3p, 3265, 3845, 387, 4893, 5594, 5879, 5880, 5894, 6654, 6655, 6850, 8611, 8612, 8613, 998, hsa-miR-122-5p, hsa-miR-126-3p, hsa-miR-145-5p, hsa-miR-155-5p, hsa-miR-21-5p, hsa-miR-29a-3p, hsa-miR-29b-3p, hsa-miR-29c-3p, hsa-miR-7-5p, 3265, 382, 3845, 4893, 5337, 5338, 5594, 5595, 5879, 5880, 5894, 6654, 6655, 6850, 8611, 8612, 8613, 998, hsa-miR-122-5p, hsa-miR-126-3p, hsa-miR-145-5p, hsa-miR-155-5p, hsa-miR-21-5p, hsa-miR-29a-3p, hsa-miR-29b-3p, hsa-miR-29c-3p, hsa-miR-34a-5p, hsa-miR-424-5p, hsa-miR-7-5p, 3265, 382, 3845, 4893, 5337, 5338, 5594, 5595, 5894, 6654, 6655, 6850, 8611, 8612, 8613, hsa-let-7a-5p, hsa-miR-122-5p, hsa-miR-126-3p, hsa-miR-145-5p, hsa-miR-155-5p, hsa-miR-21-5p, hsa-miR-29a-3p, hsa-miR-29b-3p, hsa-miR-29c-3p, hsa-miR-34a-5p, hsa-miR-424-5p, hsa-miR-7-5p, 10000, 1950, 207, 208, 3265, 3479, 382, 3845, 5154, 5155, 5337, 5338, 5594, 5595, 56034, 5728, 5894, 6654, 6655, 6850, 7039, 80310, 8611, 8612, 8613, hsa-let-7a-5p, hsa-miR-122-5p, hsa-miR-126-3p, hsa-miR-145-5p, hsa-miR-155-5p, hsa-miR-21-5p, hsa-miR-29a-3p, hsa-miR-29b-3p, hsa-miR-29c-3p, hsa-miR-34a-5p, hsa-miR-424-5p, hsa-miR-519d, hsa-miR-7-5p, 10000, 10454, 1950, 207, 208, 23118, 3265, 3479, 3845, 5154, 5155, 5594, 5595, 56034, 5728, 5894, 6654, 6655, 6885, 7039, 7189, 80310, hsa-let-7a-5p, hsa-miR-103a-3p, hsa-miR-122-5p, hsa-miR-124-3p, hsa-miR-126-3p, hsa-miR-145-5p, hsa-miR-146a-5p, hsa-miR-155-5p, hsa-miR-21-5p, hsa-miR-217, hsa-miR-221-3p, hsa-miR-26a-5p, hsa-miR-29a-3p, hsa-miR-34a-5p, hsa-miR-34b-5p, hsa-miR-424-5p, hsa-miR-519d, hsa-miR-7-5p, 10000, 10454, 1950, 207, 208, 23118, 3479, 5154, 5155, 5566, 5567, 5568, 5594, 5595, 56034, 5613, 5728, 5894, 6654, 6655, 6885, 7039, 7189, 80310, hsa-let-7a-5p, hsa-miR-103a-3p, hsa-miR-122-5p, hsa-miR-124-3p, hsa-miR-146a-5p, hsa-miR-155-5p, hsa-miR-21-5p, hsa-miR-217, hsa-miR-221-3p, hsa-miR-26a-5p, hsa-miR-29a-3p, hsa-miR-34b-5p, hsa-miR-519d, 10454, 23118, 5566, 5567, 5568, 5594, 5595, 5599, 5601, 5602, 5613, 5728, 5894, 6885, 7189, hsa-let-7a-5p, hsa-miR-103a-3p, hsa-miR-122-5p, hsa-miR-124-3p, hsa-miR-146a-5p, hsa-miR-155-5p, hsa-miR-17-5p, hsa-miR-21-5p, hsa-miR-217, hsa-miR-221-3p, hsa-miR-26a-5p, hsa-miR-29a-3p, hsa-miR-34b-5p, hsa-miR-519d, 2353, 3725, 5566, 5567, 5568, 5594, 5595, 5599, 5601, 5602, 5613, 5894, 7189, 8767, hsa-let-7a-5p, hsa-miR-103a-3p, hsa-miR-122-5p, hsa-miR-124-3p, hsa-miR-146a-5p, hsa-miR-155-5p, hsa-miR-17-5p, hsa-miR-21-5p, hsa-miR-221-3p, hsa-miR-34b-5p, 2353, 3725, 5566, 5567, 5568, 5613, 5894, 7189, 8767, hsa-let-7a-5p, hsa-miR-103a-3p, hsa-miR-124-3p, hsa-miR-146a-5p, hsa-miR-146b-5p, hsa-miR-17-5p, hsa-miR-21-5p, hsa-miR-221-3p, hsa-miR-34b-5p, 2353, 3725, 4790, 5894, 5970, 7189, 8767, hsa-let-7a-5p, hsa-miR-124-3p, hsa-miR-146a-5p, hsa-miR-146b-5p, hsa-miR-17-5p, hsa-miR-21-5p, 2353, 3663, 3725, 4790, 5970, 7189, hsa-let-7a-5p, hsa-miR-146a-5p, hsa-miR-17-5p, hsa-miR-21-5p, 2353, 3569, 3663, 3725, 4790, 5970, hsa-let-7a-5p, 2353, 3663, 3725, 4790, 5970, 7124, hsa-miR-19a-3p, 2353, 3592, 3663, 3725, 4790, 5970, 2353, 3593, 3663, 3725, 4790, 5970 |
| 69.70 | 10451, 1794, 387, 50649, 5879, 5880, 7074, 7409, 7410, 8874, 9459, hsa-miR-122-5p, 10451, 387, 50649, 5879, 5880, 5881, 7074, 7409, 7410, 8874, 9459, hsa-miR-122-5p, 10451, 387, 5879, 5880, 5881, 6850, 7074, 7409, 7410, 8874, 9459, hsa-miR-122-5p, hsa-miR-21-5p, hsa-miR-31-5p, 387, 5879, 5880, 5881, 6850, 8874, 9459, 998, hsa-miR-122-5p, hsa-miR-155-5p, hsa-miR-21-5p, hsa-miR-29a-3p, hsa-miR-29b-3p, hsa-miR-29c-3p, hsa-miR-31-5p, 3265, 3845, 387, 4893, 5879, 5880, 5881, 6654, 6655, 6850, 8874, 9459, 998, hsa-miR-122-5p, hsa-miR-145-5p, hsa-miR-155-5p, hsa-miR-21-5p, hsa-miR-29a-3p, hsa-miR-29b-3p, hsa-miR-29c-3p, 3265, 3845, 387, 4893, 5594, 5879, 5880, 5894, 6654, 6655, 6850, 8611, 8612, 8613, 998, hsa-miR-122-5p, hsa-miR-126-3p, hsa-miR-145-5p, hsa-miR-155-5p, hsa-miR-21-5p, hsa-miR-29a-3p, hsa-miR-29b-3p, hsa-miR-29c-3p, hsa-miR-7-5p, 3265, 382, 3845, 4893, 5337, 5338, 5594, 5595, 5879, 5880, 5894, 6654, 6655, 6850, 8611, 8612, 8613, 998, hsa-miR-122-5p, hsa-miR-126-3p, hsa-miR-145-5p, hsa-miR-155-5p, hsa-miR-21-5p, hsa-miR-29a-3p, hsa-miR-29b-3p, hsa-miR-29c-3p, hsa-miR-34a-5p, hsa-miR-424-5p, hsa-miR-7-5p, 3265, 382, 3845, 4893, 5337, 5338, 5594, 5595, 5894, 6654, 6655, 6850, 8611, 8612, 8613, hsa-let-7a-5p, hsa-miR-122-5p, hsa-miR-126-3p, hsa-miR-145-5p, hsa-miR-155-5p, hsa-miR-21-5p, hsa-miR-29a-3p, hsa-miR-29b-3p, hsa-miR-29c-3p, hsa-miR-34a-5p, hsa-miR-424-5p, hsa-miR-7-5p, 10000, 1950, 207, 208, 3265, 3479, 382, 3845, 5154, 5155, 5337, 5338, 5594, 5595, 56034, 5728, 5894, 6654, 6655, 6850, 7039, 80310, 8611, 8612, 8613, hsa-let-7a-5p, hsa-miR-122-5p, hsa-miR-126-3p, hsa-miR-145-5p, hsa-miR-155-5p, hsa-miR-21-5p, hsa-miR-29a-3p, hsa-miR-29b-3p, hsa-miR-29c-3p, hsa-miR-34a-5p, hsa-miR-424-5p, hsa-miR-519d, hsa-miR-7-5p, 10000, 10454, 1950, 207, 208, 23118, 3265, 3479, 3845, 5154, 5155, 5594, 5595, 56034, 5728, 5894, 6654, 6655, 6885, 7039, 7189, 80310, hsa-let-7a-5p, hsa-miR-103a-3p, hsa-miR-122-5p, hsa-miR-124-3p, hsa-miR-126-3p, hsa-miR-145-5p, hsa-miR-146a-5p, hsa-miR-155-5p, hsa-miR-21-5p, hsa-miR-217, hsa-miR-221-3p, hsa-miR-26a-5p, hsa-miR-29a-3p, hsa-miR-34a-5p, hsa-miR-34b-5p, hsa-miR-424-5p, hsa-miR-519d, hsa-miR-7-5p, 10000, 10454, 1950, 207, 208, 23118, 3479, 5154, 5155, 5566, 5567, 5568, 5594, 5595, 56034, 5613, 5728, 5894, 6654, 6655, 6885, 7039, 7189, 80310, hsa-let-7a-5p, hsa-miR-103a-3p, hsa-miR-122-5p, hsa-miR-124-3p, hsa-miR-146a-5p, hsa-miR-155-5p, hsa-miR-21-5p, hsa-miR-217, hsa-miR-221-3p, hsa-miR-26a-5p, hsa-miR-29a-3p, hsa-miR-34b-5p, hsa-miR-519d, 10454, 23118, 5566, 5567, 5568, 5594, 5595, 5599, 5601, 5602, 5613, 5728, 5894, 6885, 7189, hsa-let-7a-5p, hsa-miR-103a-3p, hsa-miR-122-5p, hsa-miR-124-3p, hsa-miR-146a-5p, hsa-miR-155-5p, hsa-miR-17-5p, hsa-miR-21-5p, hsa-miR-217, hsa-miR-221-3p, hsa-miR-26a-5p, hsa-miR-29a-3p, hsa-miR-34b-5p, hsa-miR-519d, 2353, 3725, 5566, 5567, 5568, 5594, 5595, 5599, 5601, 5602, 5613, 5894, 7189, 8767, hsa-let-7a-5p, hsa-miR-103a-3p, hsa-miR-122-5p, hsa-miR-124-3p, hsa-miR-146a-5p, hsa-miR-155-5p, hsa-miR-17-5p, hsa-miR-21-5p, hsa-miR-221-3p, hsa-miR-34b-5p, 2353, 3725, 5566, 5567, 5568, 5613, 5894, 7189, 8767, hsa-let-7a-5p, hsa-miR-103a-3p, hsa-miR-124-3p, hsa-miR-146a-5p, hsa-miR-146b-5p, hsa-miR-17-5p, hsa-miR-21-5p, hsa-miR-221-3p, hsa-miR-34b-5p, 2353, 3725, 4790, 5894, 5970, 7189, 8767, hsa-let-7a-5p, hsa-miR-124-3p, hsa-miR-146a-5p, hsa-miR-146b-5p, hsa-miR-17-5p, hsa-miR-21-5p, 2353, 3663, 3725, 4790, 5970, 7189, hsa-let-7a-5p, hsa-miR-146a-5p, hsa-miR-17-5p, hsa-miR-21-5p, 2353, 3553, 3663, 3725, 4790, 5970, hsa-miR-21-5p |
| 68.87 | 10451, 1794, 387, 50649, 5879, 5880, 7074, 7409, 7410, 8874, 9459, hsa-miR-122-5p, 10451, 387, 50649, 5879, 5880, 5881, 7074, 7409, 7410, 8874, 9459, hsa-miR-122-5p, 10451, 387, 5879, 5880, 5881, 6850, 7074, 7409, 7410, 8874, 9459, hsa-miR-122-5p, hsa-miR-21-5p, hsa-miR-31-5p, 387, 5879, 5880, 5881, 6850, 8874, 9459, 998, hsa-miR-122-5p, hsa-miR-155-5p, hsa-miR-21-5p, hsa-miR-29a-3p, hsa-miR-29b-3p, hsa-miR-29c-3p, hsa-miR-31-5p, 3265, 3845, 387, 4893, 5879, 5880, 5881, 6654, 6655, 6850, 8874, 9459, 998, hsa-miR-122-5p, hsa-miR-145-5p, hsa-miR-155-5p, hsa-miR-21-5p, hsa-miR-29a-3p, hsa-miR-29b-3p, hsa-miR-29c-3p, 3265, 3845, 387, 4893, 5594, 5879, 5880, 5894, 6654, 6655, 6850, 8611, 8612, 8613, 998, hsa-miR-122-5p, hsa-miR-126-3p, hsa-miR-145-5p, hsa-miR-155-5p, hsa-miR-21-5p, hsa-miR-29a-3p, hsa-miR-29b-3p, hsa-miR-29c-3p, hsa-miR-7-5p, 3265, 382, 3845, 4893, 5337, 5338, 5594, 5595, 5879, 5880, 5894, 6654, 6655, 6850, 8611, 8612, 8613, 998, hsa-miR-122-5p, hsa-miR-126-3p, hsa-miR-145-5p, hsa-miR-155-5p, hsa-miR-21-5p, hsa-miR-29a-3p, hsa-miR-29b-3p, hsa-miR-29c-3p, hsa-miR-34a-5p, hsa-miR-424-5p, hsa-miR-7-5p, 3265, 382, 3845, 4893, 5337, 5338, 5594, 5595, 5894, 6654, 6655, 6850, 8611, 8612, 8613, hsa-let-7a-5p, hsa-miR-122-5p, hsa-miR-126-3p, hsa-miR-145-5p, hsa-miR-155-5p, hsa-miR-21-5p, hsa-miR-29a-3p, hsa-miR-29b-3p, hsa-miR-29c-3p, hsa-miR-34a-5p, hsa-miR-424-5p, hsa-miR-7-5p, 10000, 1950, 207, 208, 3265, 3479, 382, 3845, 5154, 5155, 5337, 5338, 5594, 5595, 56034, 5728, 5894, 6654, 6655, 6850, 7039, 80310, 8611, 8612, 8613, hsa-let-7a-5p, hsa-miR-122-5p, hsa-miR-126-3p, hsa-miR-145-5p, hsa-miR-155-5p, hsa-miR-21-5p, hsa-miR-29a-3p, hsa-miR-29b-3p, hsa-miR-29c-3p, hsa-miR-34a-5p, hsa-miR-424-5p, hsa-miR-519d, hsa-miR-7-5p, 10000, 10454, 1950, 207, 208, 23118, 3265, 3479, 3845, 5154, 5155, 5594, 5595, 56034, 5728, 5894, 6654, 6655, 6885, 7039, 7189, 80310, hsa-let-7a-5p, hsa-miR-103a-3p, hsa-miR-122-5p, hsa-miR-124-3p, hsa-miR-126-3p, hsa-miR-145-5p, hsa-miR-146a-5p, hsa-miR-155-5p, hsa-miR-21-5p, hsa-miR-217, hsa-miR-221-3p, hsa-miR-26a-5p, hsa-miR-29a-3p, hsa-miR-34a-5p, hsa-miR-34b-5p, hsa-miR-424-5p, hsa-miR-519d, hsa-miR-7-5p, 10000, 10454, 1950, 207, 208, 23118, 3479, 5154, 5155, 5566, 5567, 5568, 5594, 5595, 56034, 5613, 5728, 5894, 6654, 6655, 6885, 7039, 7189, 80310, hsa-let-7a-5p, hsa-miR-103a-3p, hsa-miR-122-5p, hsa-miR-124-3p, hsa-miR-146a-5p, hsa-miR-155-5p, hsa-miR-21-5p, hsa-miR-217, hsa-miR-221-3p, hsa-miR-26a-5p, hsa-miR-29a-3p, hsa-miR-34b-5p, hsa-miR-519d, 10454, 23118, 5566, 5567, 5568, 5594, 5595, 5599, 5601, 5602, 5613, 5728, 5894, 6885, 7189, hsa-let-7a-5p, hsa-miR-103a-3p, hsa-miR-122-5p, hsa-miR-124-3p, hsa-miR-146a-5p, hsa-miR-155-5p, hsa-miR-17-5p, hsa-miR-21-5p, hsa-miR-217, hsa-miR-221-3p, hsa-miR-26a-5p, hsa-miR-29a-3p, hsa-miR-34b-5p, hsa-miR-519d, 2353, 3725, 5566, 5567, 5568, 5594, 5595, 5599, 5601, 5602, 5613, 5894, 7189, 8767, hsa-let-7a-5p, hsa-miR-103a-3p, hsa-miR-122-5p, hsa-miR-124-3p, hsa-miR-146a-5p, hsa-miR-155-5p, hsa-miR-17-5p, hsa-miR-21-5p, hsa-miR-221-3p, hsa-miR-34b-5p, 2353, 3725, 5566, 5567, 5568, 5613, 5894, 7189, 8767, hsa-let-7a-5p, hsa-miR-103a-3p, hsa-miR-124-3p, hsa-miR-146a-5p, hsa-miR-146b-5p, hsa-miR-17-5p, hsa-miR-21-5p, hsa-miR-221-3p, hsa-miR-34b-5p, 2353, 3725, 4790, 5894, 5970, 7189, 8767, hsa-let-7a-5p, hsa-miR-124-3p, hsa-miR-146a-5p, hsa-miR-146b-5p, hsa-miR-17-5p, hsa-miR-21-5p, 2353, 3663, 3725, 4790, 5970, 7189, hsa-let-7a-5p, hsa-miR-146a-5p, hsa-miR-17-5p, hsa-miR-21-5p, 2353, 3576, 3663, 3725, 4790, 5970, hsa-miR-17-5p, hsa-miR-520b |
| 68.57 | 10451, 1794, 387, 50649, 5879, 5880, 7074, 7409, 7410, 8874, 9459, hsa-miR-122-5p, 10451, 387, 50649, 5879, 5880, 5881, 7074, 7409, 7410, 8874, 9459, hsa-miR-122-5p, 10451, 387, 5879, 5880, 5881, 6850, 7074, 7409, 7410, 8874, 9459, hsa-miR-122-5p, hsa-miR-21-5p, hsa-miR-31-5p, 387, 5879, 5880, 5881, 6850, 8874, 9459, 998, hsa-miR-122-5p, hsa-miR-155-5p, hsa-miR-21-5p, hsa-miR-29a-3p, hsa-miR-29b-3p, hsa-miR-29c-3p, hsa-miR-31-5p, 3265, 3845, 387, 4893, 5879, 5880, 5881, 6654, 6655, 6850, 8874, 9459, 998, hsa-miR-122-5p, hsa-miR-145-5p, hsa-miR-155-5p, hsa-miR-21-5p, hsa-miR-29a-3p, hsa-miR-29b-3p, hsa-miR-29c-3p, 3265, 3845, 387, 4893, 5594, 5879, 5880, 5894, 6654, 6655, 6850, 8611, 8612, 8613, 998, hsa-miR-122-5p, hsa-miR-126-3p, hsa-miR-145-5p, hsa-miR-155-5p, hsa-miR-21-5p, hsa-miR-29a-3p, hsa-miR-29b-3p, hsa-miR-29c-3p, hsa-miR-7-5p, 3265, 382, 3845, 4893, 5337, 5338, 5594, 5595, 5879, 5880, 5894, 6654, 6655, 6850, 8611, 8612, 8613, 998, hsa-miR-122-5p, hsa-miR-126-3p, hsa-miR-145-5p, hsa-miR-155-5p, hsa-miR-21-5p, hsa-miR-29a-3p, hsa-miR-29b-3p, hsa-miR-29c-3p, hsa-miR-34a-5p, hsa-miR-424-5p, hsa-miR-7-5p, 3265, 382, 3845, 4893, 5337, 5338, 5594, 5595, 5894, 6654, 6655, 6850, 8611, 8612, 8613, hsa-let-7a-5p, hsa-miR-122-5p, hsa-miR-126-3p, hsa-miR-145-5p, hsa-miR-155-5p, hsa-miR-21-5p, hsa-miR-29a-3p, hsa-miR-29b-3p, hsa-miR-29c-3p, hsa-miR-34a-5p, hsa-miR-424-5p, hsa-miR-7-5p, 10000, 1950, 207, 208, 3265, 3479, 382, 3845, 5154, 5155, 5337, 5338, 5594, 5595, 56034, 5728, 5894, 6654, 6655, 6850, 7039, 80310, 8611, 8612, 8613, hsa-let-7a-5p, hsa-miR-122-5p, hsa-miR-126-3p, hsa-miR-145-5p, hsa-miR-155-5p, hsa-miR-21-5p, hsa-miR-29a-3p, hsa-miR-29b-3p, hsa-miR-29c-3p, hsa-miR-34a-5p, hsa-miR-424-5p, hsa-miR-519d, hsa-miR-7-5p, 10000, 10454, 1950, 207, 208, 23118, 3265, 3479, 3845, 5154, 5155, 5594, 5595, 56034, 5728, 5894, 6654, 6655, 6885, 7039, 7189, 80310, hsa-let-7a-5p, hsa-miR-103a-3p, hsa-miR-122-5p, hsa-miR-124-3p, hsa-miR-126-3p, hsa-miR-145-5p, hsa-miR-146a-5p, hsa-miR-155-5p, hsa-miR-21-5p, hsa-miR-217, hsa-miR-221-3p, hsa-miR-26a-5p, hsa-miR-29a-3p, hsa-miR-34a-5p, hsa-miR-34b-5p, hsa-miR-424-5p, hsa-miR-519d, hsa-miR-7-5p, 10000, 10454, 1950, 207, 208, 23118, 3479, 5154, 5155, 5566, 5567, 5568, 5594, 5595, 56034, 5613, 5728, 5894, 6654, 6655, 6885, 7039, 7189, 80310, hsa-let-7a-5p, hsa-miR-103a-3p, hsa-miR-122-5p, hsa-miR-124-3p, hsa-miR-146a-5p, hsa-miR-155-5p, hsa-miR-21-5p, hsa-miR-217, hsa-miR-221-3p, hsa-miR-26a-5p, hsa-miR-29a-3p, hsa-miR-34b-5p, hsa-miR-519d, 10454, 23118, 5566, 5567, 5568, 5594, 5595, 5599, 5601, 5602, 5613, 5728, 5894, 6885, 7189, hsa-let-7a-5p, hsa-miR-103a-3p, hsa-miR-122-5p, hsa-miR-124-3p, hsa-miR-146a-5p, hsa-miR-155-5p, hsa-miR-17-5p, hsa-miR-21-5p, hsa-miR-217, hsa-miR-221-3p, hsa-miR-26a-5p, hsa-miR-29a-3p, hsa-miR-34b-5p, hsa-miR-519d, 2353, 3725, 5566, 5567, 5568, 5594, 5595, 5599, 5601, 5602, 5613, 5894, 7189, 8767, hsa-let-7a-5p, hsa-miR-103a-3p, hsa-miR-122-5p, hsa-miR-124-3p, hsa-miR-146a-5p, hsa-miR-155-5p, hsa-miR-17-5p, hsa-miR-21-5p, hsa-miR-221-3p, hsa-miR-34b-5p, 2353, 3725, 5566, 5567, 5568, 5613, 5894, 7189, 8767, hsa-let-7a-5p, hsa-miR-103a-3p, hsa-miR-124-3p, hsa-miR-146a-5p, hsa-miR-146b-5p, hsa-miR-17-5p, hsa-miR-21-5p, hsa-miR-221-3p, hsa-miR-34b-5p, 2353, 3725, 4790, 5894, 5970, 7189, 8767, hsa-let-7a-5p, hsa-miR-124-3p, hsa-miR-146a-5p, hsa-miR-146b-5p, hsa-miR-17-5p, hsa-miR-21-5p, 4790, 4792, 5894, 5970, 7189, 8767, hsa-miR-124-3p, hsa-miR-146a-5p, hsa-miR-146b-5p, 1147, 1326, 4790, 4792, 5894, 7189, 8767, hsa-miR-146a-5p, hsa-miR-146b-5p |
| 66.41 | 10451, 1794, 387, 50649, 5879, 5880, 7074, 7409, 7410, 8874, 9459, hsa-miR-122-5p, 10451, 387, 50649, 5879, 5880, 5881, 7074, 7409, 7410, 8874, 9459, hsa-miR-122-5p, 10451, 387, 5879, 5880, 5881, 6850, 7074, 7409, 7410, 8874, 9459, hsa-miR-122-5p, hsa-miR-21-5p, hsa-miR-31-5p, 387, 5879, 5880, 5881, 6850, 8874, 9459, 998, hsa-miR-122-5p, hsa-miR-155-5p, hsa-miR-21-5p, hsa-miR-29a-3p, hsa-miR-29b-3p, hsa-miR-29c-3p, hsa-miR-31-5p, 3265, 3845, 387, 4893, 5879, 5880, 5881, 6654, 6655, 6850, 8874, 9459, 998, hsa-miR-122-5p, hsa-miR-145-5p, hsa-miR-155-5p, hsa-miR-21-5p, hsa-miR-29a-3p, hsa-miR-29b-3p, hsa-miR-29c-3p, 3265, 3845, 387, 4893, 5594, 5879, 5880, 5894, 6654, 6655, 6850, 8611, 8612, 8613, 998, hsa-miR-122-5p, hsa-miR-126-3p, hsa-miR-145-5p, hsa-miR-155-5p, hsa-miR-21-5p, hsa-miR-29a-3p, hsa-miR-29b-3p, hsa-miR-29c-3p, hsa-miR-7-5p, 3265, 382, 3845, 4893, 5337, 5338, 5594, 5595, 5879, 5880, 5894, 6654, 6655, 6850, 8611, 8612, 8613, 998, hsa-miR-122-5p, hsa-miR-126-3p, hsa-miR-145-5p, hsa-miR-155-5p, hsa-miR-21-5p, hsa-miR-29a-3p, hsa-miR-29b-3p, hsa-miR-29c-3p, hsa-miR-34a-5p, hsa-miR-424-5p, hsa-miR-7-5p, 3265, 382, 3845, 4893, 5337, 5338, 5594, 5595, 5879, 5880, 5894, 6850, 7454, 8611, 8612, 8613, 8976, 998, hsa-miR-34a-5p, hsa-miR-424-5p, 3265, 3845, 4893, 5594, 5595, 5604, 5605, 5894, 6850, 7454, 8976, hsa-miR-34a-5p, hsa-miR-424-5p, 5594, 5604, 5605, 6850, 7454, 8976, hsa-miR-335-5p, 10163, 55845, 60, 71, 7454, 81873, 8936, 8976, hsa-miR-335-5p, 10093, 10163, 55845, 60, 71, 7454, 8936, 8976, 10094, 10163, 55845, 60, 71, 7454, 8936, 8976, 10095, 10163, 55845, 60, 71, 7454, 8936, 8976, 10109, 10163, 55845, 60, 71, 7454, 8936, 8976, 10163, 10552, 55845, 60, 71, 7454, 8936, 8976 |

**Table S8. 118 Deregulated Pathway elements of Primary Myelofibrosis compared to peripheral blood samples.**

| **Entrez Gene ID** | **Gene Symbol** |
| --- | --- |
| 10000 | AKT3 |
| 10368 | CACNG3 |
| 10369 | CACNG2 |
| 10451 | VAV3 |
| 10454 | TAB1 |
| 10488 | CREB3 |
| 116443 | GRIN3A |
| 116444 | GRIN3B |
| 1386 | ATF2 |
| 1388 | ATF6B |
| 148327 | CREB3L4 |
| 163688 | CALML6 |
| 1794 | DOCK2 |
| 1950 | EGF |
| 207 | AKT1 |
| 208 | AKT2 |
| 23118 | TAB2 |
| 27091 | CACNG5 |
| 27092 | CACNG4 |
| 2890 | GRIA1 |
| 2892 | GRIA3 |
| 2893 | GRIA4 |
| 2902 | GRIN1 |
| 2903 | GRIN2A |
| 2904 | GRIN2B |
| 2905 | GRIN2C |
| 2906 | GRIN2D |
| 3265 | HRAS |
| 3479 | IGF1 |
| 382 | ARF6 |
| 3845 | KRAS |
| 387 | RHOA |
| 468 | ATF4 |
| 4893 | NRAS |
| 50649 | ARHGEF4 |
| 5154 | PDGFA |
| 5155 | PDGFB |
| 51806 | CALML5 |
| 5337 | PLD1 |
| 5338 | PLD2 |
| 5350 | PLN |
| 5566 | PRKACA |
| 5567 | PRKACB |
| 5568 | PRKACG |
| 55799 | CACNA2D3 |
| 5594 | MAPK1 |
| 5595 | MAPK3 |
| 56034 | PDGFC |
| 5613 | PRKX |
| 5728 | PTEN |
| 5879 | RAC1 |
| 5880 | RAC2 |
| 5881 | RAC3 |
| 5894 | RAF1 |
| 59283 | CACNG8 |
| 59284 | CACNG7 |
| 59285 | CACNG6 |
| 6262 | RYR2 |
| 64764 | CREB3L2 |
| 6654 | SOS1 |
| 6655 | SOS2 |
| 6850 | SYK |
| 6885 | MAP3K7 |
| 7039 | TGFA |
| 7074 | TIAM1 |
| 7189 | TRAF6 |
| 7409 | VAV1 |
| 7410 | VAV2 |
| 775 | CACNA1C |
| 776 | CACNA1D |
| 778 | CACNA1F |
| 779 | CACNA1S |
| 781 | CACNA2D1 |
| 782 | CACNB1 |
| 783 | CACNB2 |
| 784 | CACNB3 |
| 785 | CACNB4 |
| 786 | CACNG1 |
| 801 | CALM1 |
| 80310 | PDGFD |
| 805 | CALM2 |
| 808 | CALM3 |
| 810 | CALML3 |
| 84152 | PPP1R1B |
| 84699 | CREB3L3 |
| 8611 | PPAP2A |
| 8612 | PPAP2C |
| 8613 | PPAP2B |
| 8874 | ARHGEF7 |
| 90993 | CREB3L1 |
| 9254 | CACNA2D2 |
| 93589 | CACNA2D4 |
| 9459 | ARHGEF6 |
| 9586 | CREB5 |
| 998 | CDC42 |
| hsa-let-7a-5p | hsa-let-7a-5p |
| hsa-miR-103a-3p | hsa-miR-103a-3p |
| hsa-miR-122-5p | hsa-miR-122-5p |
| hsa-miR-124-3p | hsa-miR-124-3p |
| hsa-miR-125b-5p | hsa-miR-125b-5p |
| hsa-miR-126-3p | hsa-miR-126-3p |
| hsa-miR-133a | hsa-miR-133a |
| hsa-miR-145-5p | hsa-miR-145-5p |
| hsa-miR-146a-5p | hsa-miR-146a-5p |
| hsa-miR-155-5p | hsa-miR-155-5p |
| hsa-miR-21-5p | hsa-miR-21-5p |
| hsa-miR-217 | hsa-miR-217 |
| hsa-miR-221-3p | hsa-miR-221-3p |
| hsa-miR-26a-5p | hsa-miR-26a-5p |
| hsa-miR-29a-3p | hsa-miR-29a-3p |
| hsa-miR-29b-3p | hsa-miR-29b-3p |
| hsa-miR-29c-3p | hsa-miR-29c-3p |
| hsa-miR-31-5p | hsa-miR-31-5p |
| hsa-miR-34a-5p | hsa-miR-34a-5p |
| hsa-miR-34b-5p | hsa-miR-34b-5p |
| hsa-miR-424-5p | hsa-miR-424-5p |
| hsa-miR-519d | hsa-miR-519d |
| hsa-miR-7-5p | hsa-miR-7-5p |

**3) Comparison between the two analyses with different normal references.**

**Figure S1. At the whole pathway level**


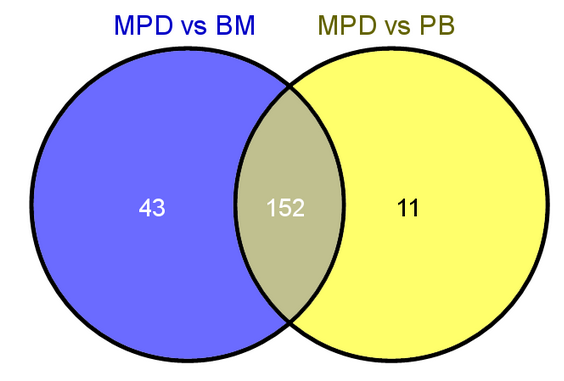


43 Elements only in "MPD vs PB":

Chagas disease (American trypanosomiasis)

Cocaine addiction

Drug metabolism - other enzymes

HTLV-I infection

Hepatitis B

Leukocyte transendothelial migration

Shigellosis

Taurine and hypotaurine metabolism

Cell cycle

Propanoate metabolism

Focal adhesion

11 Elements only in "MPD vs BM":

Acute myeloid leukemia

Alanine, aspartate and glutamate metabolism

Basal cell carcinoma

Cardiac muscle contraction

Chemical carcinogenesis

Circadian entrainment

Circadian rhythm

Drug metabolism - cytochrome P450

Epstein-Barr virus infection

Ether lipid metabolism

Fatty acid metabolism

GABAergic synapse

Glycerolipid metabolism

Glycerophospholipid metabolism

Hippo signaling pathway

Long-term potentiation

Lysine biosynthesis

Lysine degradation

Mucin type O-Glycan biosynthesis

Nicotinate and nicotinamide metabolism

Oxidative phosphorylation

Phosphatidylinositol signaling system

Progesterone-mediated oocyte maturation

Retinol metabolism

Sphingolipid metabolism

Synthesis and degradation of ketone bodies

TGF-beta signaling pathway

Type I diabetes mellitus

Tyrosine metabolism

Valine, leucine and isoleucine degradation

Viral carcinogenesis

Wnt signaling pathway

Glycosphingolipid biosynthesis - lacto and neolacto series

Metabolism of xenobiotics by cytochrome P450

One carbon pool by folate

Pyruvate metabolism

Steroid hormone biosynthesis

Taste transduction

Lipoic acid metabolism

Pyrimidine metabolism

Purine metabolism

alpha-Linolenic acid metabolism

Caffeine metabolism

**Figure S2. Comparison of the 2 lists of partecipants (genes and miRNAs)**


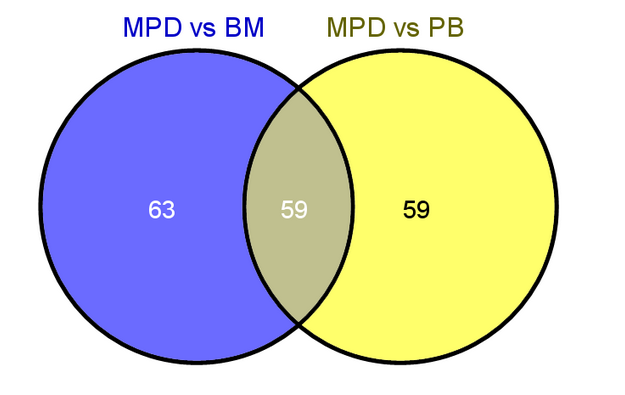


*63 elements only in "MPD vs BM":*

CLEC4M

CHUK

CREBBP

CRK

CRKL

DOCK1

ENO1

ENO2

ENO3

EP300

EPO

ARHGEF12

GAB1

ARHGEF26

ANGPT1

ANGPT2

CD209

HIF1A

HK1

HK3

RHOG

LDHA

ARNT

LTBR

NFKB1

NFKBIA

NOS3

NPPA

ANGPT4

PDK1

PFKFB1

PFKFB2

PFKFB3

PFKFB4

PFKL

PGF

PGK1

PIK3R2

PPP1CA

RELA

SLC2A1

TEK

TF

TIMP1

VEGFB

VEGFC

HKDC1

ELMO1

hsa-miR-106b-5p

hsa-miR-1244

hsa-miR-146b-5p

hsa-miR-17-5p

hsa-miR-182-5p

hsa-miR-185-5p

hsa-miR-195-5p

hsa-miR-19a-3p

hsa-miR-203a

hsa-miR-20a-5p

hsa-miR-22-3p

hsa-miR-222-3p

hsa-miR-375

hsa-miR-494

hsa-miR-93-5p

*59 Common elements in "MPD vs BM" and "MPD vs PB":*

AKT3

CACNG3

CACNG2

GRIN3A

GRIN3B

AKT1

AKT2

CACNG5

CACNG4

GRIA1

GRIA3

GRIA4

GRIN1

GRIN2B

GRIN2C

GRIN2D

RHOA

PLN

PRKACA

PRKACB

PRKACG

CACNA2D3

PRKX

PTEN

RAF1

CACNG8

CACNG7

CACNG6

RYR2

TRAF6

CACNA1C

CACNA1D

CACNA1F

CACNA1S

CACNA2D1

CACNB1

CACNB2

CACNB3

CACNB4

CACNG1

CACNA2D2

CACNA2D4

CDC42

hsa-miR-122-5p

hsa-miR-124-3p

hsa-miR-125b-5p

hsa-miR-133a

hsa-miR-145-5p

hsa-miR-146a-5p

hsa-miR-155-5p

hsa-miR-21-5p

hsa-miR-221-3p

hsa-miR-26a-5p

hsa-miR-29a-3p

hsa-miR-29b-3p

hsa-miR-31-5p

hsa-miR-34a-5p

hsa-miR-34b-5p

hsa-miR-519d

*59 Elements only in "MPD vs PB":*

VAV3

TAB1

CREB3

ATF2

ATF6B

CREB3L4

CALML6

DOCK2

EGF

TAB2

GRIN2A

HRAS

IGF1

ARF6

KRAS

ATF4

NRAS

ARHGEF4

PDGFA

PDGFB

CALML5

PLD1

PLD2

MAPK1

MAPK3

PDGFC

RAC1

RAC2

RAC3

CREB3L2

SOS1

SOS2

SYK

MAP3K7

TGFA

TIAM1

VAV1

VAV2

CALM1

PDGFD

CALM2

CALM3

CALML3

PPP1R1B

CREB3L3

PPAP2A

PPAP2C

PPAP2B

ARHGEF7

CREB3L1

ARHGEF6

CREB5

hsa-let-7a-5p

hsa-miR-103a-3p

hsa-miR-126-3p

hsa-miR-217

hsa-miR-29c-3p

hsa-miR-424-5p

hsa-miR-7-5p

**4) MAGIA results considering PMF samples separately with CTR PB and CTR BM samples.**

**Table S9.** Pearson correlation between miRNAs and gene-targets results (FDR threshold at 0.1) in the comparison PMF vs CTR PB

| **correlation** | **N° relations** | **N° miRNAs** | **N° genes** |
| --- | --- | --- | --- |
| -1 | 0 | 0 | 0 |
| -0.9 | 0 | 0 | 0 |
| -0.8 | 0 | 0 | 0 |
| -0.7 | 2 | 1 | 2 |
| -0.6 | 49 | 14 | 48 |
| -0.5 | 442 | 43 | 428 |

**Table S10.** Pearson correlation between miRNAs and gene-targets results (FDR threshold at 0.1) in the comparison PMF vs CTR BM

| **correlation** | **N° relations** | **N° miRNAs** | **N° genes** |
| --- | --- | --- | --- |
| -1 | 0 | 0 | 0 |
| -0.9 | 0 | 0 | 0 |
| -0.8 | 0 | 0 | 0 |
| -0.7 | 1 | 1 | 1 |
| -0.6 | 25 | 15 | 24 |
| -0.5 | 250 | 54 | 236 |

**5) MAGIA^2^ results considering PMF samples separately with CTR PB and CTR BM samples.**

**Table S11.** Numbers of identified circuits, and of involved genes, miRNAs and TFs obtained by MAGIA^2^

| **Comparison** | **N° circuits** | **N° mRNA** | **N° TF** | **N° miRNAs** |
| --- | --- | --- | --- | --- |
| PMF-BM | 340 | 236 | 21 | 29 |
| PMF-PB | 1537 | 603 | 18 | 24 |

**6) Interactions (edges) that belong to circuits results from MAGIA^2^ with correlation greater than 0.5 in the PMF vs PB comparison, visualized in the final network (Supplementary Figure 3)**

**Table S12.** In the first column there are the interactions. Entrez gene/miRNA ids represent the nodes.

Into the brackets is indicated if the correlation between the two nodes is positive or negative.

In the second column indicates the type of circuits to which the interactions belong: triplets1, TF regulates mRNA and miRNA, and miRNA regulates mRNA; triplets2, miRNA regulates mRNA and TF, and TF regulates mRNA.

| **Interactions** | **Circuit_type** |
| --- | --- |
| 4609 (negative) 57659 | triplets1 |
| 4609 (negative) 57659 | triplets1 |
| 4609 (negative) 57659 | triplets1 |
| 4609 (negative) 57659 | triplets1 |
| 4609 (positive) 26278 | triplets1 |
| 4609 (positive) 10527 | triplets1 |
| 1386 (positive) 9616 | triplets1 |
| 1050 (negative) 488 | triplets1 |
| 4609 (positive) 23463 | triplets1 |
| 4609 (positive) 23463 | triplets1 |
| 4609 (positive) 26278 | triplets1 |
| 4609 (positive) 10527 | triplets1 |
| 4609 (positive) 3312 | triplets1 |
| 4609 (negative) 80829 | triplets1 |
| 1386 (positive) 4209 | triplets1 |
| 4609 (negative) 22919 | triplets1 |
| 4609 (negative) 22919 | triplets1 |
| 4609 (negative) 22919 | triplets1 |
| 4609 (negative) 22919 | triplets1 |
| 4609 (positive) 23463 | triplets1 |
| 4609 (positive) 26278 | triplets1 |
| 4609 (positive) 10527 | triplets1 |
| 1386 (positive) 25852 | triplets1 |
| 4609 (positive) 26278 | triplets1 |
| 1050 (negative) 4092 | triplets1 |
| 1050 (negative) 4092 | triplets1 |
| 1386 (positive) 23119 | triplets1 |
| 1050 (positive) 23269 | triplets1 |
| 1050 (negative) 116931 | triplets1 |
| 4609 (positive) 23463 | triplets1 |
| 4609 (negative) 9586 | triplets1 |
| 4609 (positive) 23463 | triplets1 |
| 4609 (positive) 10527 | triplets1 |
| 4609 (negative) 80829 | triplets1 |
| 4609 (negative) 51621 | triplets1 |
| 1050 (negative) 4209 | triplets1 |
| 1050 (negative) 116931 | triplets1 |
| 1050 (positive) 9321 | triplets1 |
| 4609 (negative) 687 | triplets1 |
| 4609 (negative) 7799 | triplets1 |
| 4609 (negative) 9066 | triplets1 |
| 4609 (negative) 440730 | triplets1 |
| 4609 (positive) 6895 | triplets1 |
| 4609 (negative) 9586 | triplets1 |
| 4609 (negative) 4893 | triplets1 |
| 4609 (negative) 9586 | triplets1 |
| 4609 (positive) 10611 | triplets1 |
| 4609 (positive) 10611 | triplets1 |
| 4609 (negative) 10642 | triplets1 |
| 1050 (negative) 160760 | triplets1 |
| 4609 (negative) 9586 | triplets1 |
| 1050 (negative) 91584 | triplets1 |
| 1050 (negative) 8874 | triplets1 |
| 1386 (positive) 9658 | triplets1 |
| 1386 (positive) 687 | triplets1 |
| 4609 (negative) 8445 | triplets1 |
| 4609 (negative) 1026 | triplets1 |
| 1386 (positive) 3090 | triplets1 |
| 1386 (negative) 23129 | triplets1 |
| 4609 (negative) 1026 | triplets1 |
| 4609 (negative) 1026 | triplets1 |
| 1050 (negative) 8874 | triplets1 |
| 5966 (positive) 10725 | triplets1 |
| 5966 (positive) 80223 | triplets1 |
| 5966 (positive) 80829 | triplets1 |
| 1050 (positive) 9321 | triplets1 |
| 1050 (negative) 221833 | triplets1 |
| 4609 (positive) hsa-mir-106a-5p | triplets1 |
| 4609 (positive) hsa-mir-17-5p | triplets1 |
| 4609 (positive) hsa-mir-20a-5p | triplets1 |
| 4609 (positive) hsa-mir-20b-5p | triplets1 |
| 4609 (positive) hsa-mir-106a-5p | triplets1 |
| 4609 (positive) hsa-mir-20b-5p | triplets1 |
| 1386 (positive) hsa-let-7d-5p | triplets1 |
| 1050 (negative) hsa-let-7d-5p | triplets1 |
| 4609 (positive) hsa-mir-106a-5p | triplets1 |
| 4609 (positive) hsa-mir-20b-5p | triplets1 |
| 4609 (positive) hsa-mir-20b-5p | triplets1 |
| 4609 (positive) hsa-mir-106a-5p | triplets1 |
| 4609 (positive) hsa-mir-20b-5p | triplets1 |
| 4609 (positive) hsa-mir-106a-5p | triplets1 |
| 1386 (positive) hsa-let-7d-5p | triplets1 |
| 4609 (positive) hsa-mir-106a-5p | triplets1 |
| 4609 (positive) hsa-mir-20b-5p | triplets1 |
| 4609 (positive) hsa-mir-17-5p | triplets1 |
| 4609 (positive) hsa-mir-20a-5p | triplets1 |
| 4609 (positive) hsa-mir-20a-5p | triplets1 |
| 4609 (positive) hsa-mir-20a-5p | triplets1 |
| 4609 (positive) hsa-mir-20a-5p | triplets1 |
| 1386 (positive) hsa-let-7d-5p | triplets1 |
| 4609 (positive) hsa-mir-17-5p | triplets1 |
| 1050 (positive) hsa-mir-17-5p | triplets1 |
| 1050 (positive) hsa-mir-20a-5p | triplets1 |
| 1386 (positive) hsa-let-7d-5p | triplets1 |
| 1050 (negative) hsa-let-7d-5p | triplets1 |
| 1050 (positive) hsa-mir-20a-5p | triplets1 |
| 4609 (negative) hsa-let-7d-5p | triplets1 |
| 4609 (positive) hsa-mir-20a-5p | triplets1 |
| 4609 (positive) hsa-mir-17-5p | triplets1 |
| 4609 (positive) hsa-mir-17-5p | triplets1 |
| 4609 (positive) hsa-mir-17-5p | triplets1 |
| 4609 (positive) hsa-mir-19b-3p | triplets1 |
| 1050 (negative) hsa-let-7d-5p | triplets1 |
| 1050 (positive) hsa-mir-17-5p | triplets1 |
| 1050 (positive) hsa-mir-17-5p | triplets1 |
| 4609 (negative) hsa-let-7d-5p | triplets1 |
| 4609 (negative) hsa-let-7d-5p | triplets1 |
| 4609 (negative) hsa-let-7d-5p | triplets1 |
| 4609 (negative) hsa-let-7d-5p | triplets1 |
| 4609 (negative) hsa-let-7d-5p | triplets1 |
| 4609 (positive) hsa-mir-20b-5p | triplets1 |
| 4609 (negative) hsa-let-7d-5p | triplets1 |
| 4609 (positive) hsa-mir-106a-5p | triplets1 |
| 4609 (positive) hsa-mir-106a-5p | triplets1 |
| 4609 (positive) hsa-mir-20b-5p | triplets1 |
| 4609 (positive) hsa-mir-20b-5p | triplets1 |
| 1050 (negative) hsa-let-7d-5p | triplets1 |
| 4609 (positive) hsa-mir-17-5p | triplets1 |
| 1050 (negative) hsa-let-7d-5p | triplets1 |
| 1050 (positive) hsa-mir-17-5p | triplets1 |
| 1386 (positive) hsa-let-7d-5p | triplets1 |
| 1386 (positive) hsa-let-7d-5p | triplets1 |
| 4609 (negative) hsa-let-7d-5p | triplets1 |
| 4609 (positive) hsa-mir-20b-5p | triplets1 |
| 1386 (positive) hsa-let-7d-5p | triplets1 |
| 1386 (positive) hsa-let-7d-5p | triplets1 |
| 4609 (positive) hsa-mir-106a-5p | triplets1 |
| 4609 (positive) hsa-mir-17-5p | triplets1 |
| 1050 (negative) hsa-let-7d-5p | triplets1 |
| 5966 (negative) hsa-mir-17-5p | triplets1 |
| 5966 (negative) hsa-mir-17-5p | triplets1 |
| 5966 (negative) hsa-mir-17-5p | triplets1 |
| 1050 (positive) hsa-mir-20a-5p | triplets1 |
| 1050 (positive) hsa-mir-17-5p | triplets1 |
| 57659 (negative) hsa-mir-106a-5p | triplets1 |
| 57659 (negative) hsa-mir-17-5p | triplets1 |
| 57659 (negative) hsa-mir-20a-5p | triplets1 |
| 57659 (negative) hsa-mir-20b-5p | triplets1 |
| 26278 (positive) hsa-mir-106a-5p | triplets1 |
| 10527 (positive) hsa-mir-20b-5p | triplets1 |
| 9616 (positive) hsa-let-7d-5p | triplets1 |
| 488 (positive) hsa-let-7d-5p | triplets1 |
| 23463 (positive) hsa-mir-106a-5p | triplets1 |
| 23463 (positive) hsa-mir-20b-5p | triplets1 |
| 26278 (positive) hsa-mir-20b-5p | triplets1 |
| 10527 (positive) hsa-mir-106a-5p | triplets1 |
| 3312 (positive) hsa-mir-20b-5p | triplets1 |
| 80829 (negative) hsa-mir-106a-5p | triplets1 |
| 4209 (positive) hsa-let-7d-5p | triplets1 |
| 22919 (negative) hsa-mir-106a-5p | triplets1 |
| 22919 (negative) hsa-mir-20b-5p | triplets1 |
| 22919 (negative) hsa-mir-17-5p | triplets1 |
| 22919 (negative) hsa-mir-20a-5p | triplets1 |
| 23463 (positive) hsa-mir-20a-5p | triplets1 |
| 26278 (positive) hsa-mir-20a-5p | triplets1 |
| 10527 (positive) hsa-mir-20a-5p | triplets1 |
| 25852 (positive) hsa-let-7d-5p | triplets1 |
| 26278 (positive) hsa-mir-17-5p | triplets1 |
| 4092 (negative) hsa-mir-17-5p | triplets1 |
| 4092 (negative) hsa-mir-20a-5p | triplets1 |
| 23119 (positive) hsa-let-7d-5p | triplets1 |
| 23269 (negative) hsa-let-7d-5p | triplets1 |
| 116931 (negative) hsa-mir-20a-5p | triplets1 |
| 23463 (negative) hsa-let-7d-5p | triplets1 |
| 9586 (negative) hsa-mir-20a-5p | triplets1 |
| 23463 (positive) hsa-mir-17-5p | triplets1 |
| 10527 (positive) hsa-mir-17-5p | triplets1 |
| 80829 (negative) hsa-mir-17-5p | triplets1 |
| 51621 (negative) hsa-mir-19b-3p | triplets1 |
| 4209 (positive) hsa-let-7d-5p | triplets1 |
| 116931 (negative) hsa-mir-17-5p | triplets1 |
| 9321 (positive) hsa-mir-17-5p | triplets1 |
| 687 (positive) hsa-let-7d-5p | triplets1 |
| 7799 (positive) hsa-let-7d-5p | triplets1 |
| 9066 (positive) hsa-let-7d-5p | triplets1 |
| 440730 (positive) hsa-let-7d-5p | triplets1 |
| 6895 (negative) hsa-let-7d-5p | triplets1 |
| 9586 (negative) hsa-mir-20b-5p | triplets1 |
| 4893 (positive) hsa-let-7d-5p | triplets1 |
| 9586 (negative) hsa-mir-106a-5p | triplets1 |
| 10611 (positive) hsa-mir-106a-5p | triplets1 |
| 10611 (positive) hsa-mir-20b-5p | triplets1 |
| 10642 (negative) hsa-mir-20b-5p | triplets1 |
| 160760 (positive) hsa-let-7d-5p | triplets1 |
| 9586 (negative) hsa-mir-17-5p | triplets1 |
| 91584 (positive) hsa-let-7d-5p | triplets1 |
| 8874 (negative) hsa-mir-17-5p | triplets1 |
| 9658 (positive) hsa-let-7d-5p | triplets1 |
| 687 (positive) hsa-let-7d-5p | triplets1 |
| 8445 (positive) hsa-let-7d-5p | triplets1 |
| 1026 (negative) hsa-mir-20b-5p | triplets1 |
| 3090 (positive) hsa-let-7d-5p | triplets1 |
| 23129 (negative) hsa-let-7d-5p | triplets1 |
| 1026 (negative) hsa-mir-106a-5p | triplets1 |
| 1026 (negative) hsa-mir-17-5p | triplets1 |
| 8874 (positive) hsa-let-7d-5p | triplets1 |
| 10725 (negative) hsa-mir-17-5p | triplets1 |
| 80223 (negative) hsa-mir-17-5p | triplets1 |
| 80829 (negative) hsa-mir-17-5p | triplets1 |
| 9321 (positive) hsa-mir-20a-5p | triplets1 |
| 221833 (negative) hsa-mir-17-5p | triplets1 |
| 3659 (positive) 10746 | triplets2 |
| 3659 (negative) 26278 | triplets2 |
| 3659 (negative) 9321 | triplets2 |
| 3659 (negative) 27042 | triplets2 |
| 3659 (positive) 53344 | triplets2 |
| 3659 (positive) 23143 | triplets2 |
| 3659 (positive) 220965 | triplets2 |
| 3659 (positive) 10746 | triplets2 |
| 3659 (negative) 26278 | triplets2 |
| 3659 (negative) 9321 | triplets2 |
| 3659 (negative) 27042 | triplets2 |
| 3659 (positive) 53344 | triplets2 |
| 3659 (positive) 200424 | triplets2 |
| 3659 (positive) 169792 | triplets2 |
| 3659 (positive) 668 | triplets2 |
| 3659 (positive) 668 | triplets2 |
| 3659 (negative) 57496 | triplets2 |
| 3659 (negative) 57496 | triplets2 |
| 55810 (negative) 10622 | triplets2 |
| 55810 (negative) 10622 | triplets2 |
| 55810 (positive) 8754 | triplets2 |
| 3659 (negative) 26278 | triplets2 |
| 55810 (positive) 8754 | triplets2 |
| 3659 (negative) 27042 | triplets2 |
| 3659 (negative) 53944 | triplets2 |
| 55810 (positive) 56603 | triplets2 |
| 55810 (negative) 10622 | triplets2 |
| 55810 (negative) 10611 | triplets2 |
| 55810 (negative) 10622 | triplets2 |
| 3659 (negative) 53944 | triplets2 |
| 3659 (positive) 22941 | triplets2 |
| 3659 (positive) 22941 | triplets2 |
| 3659 (negative) 53944 | triplets2 |
| 55810 (positive) 56603 | triplets2 |
| 55810 (negative) 10611 | triplets2 |
| 55810 (positive) 56603 | triplets2 |
| 55810 (positive) 8754 | triplets2 |
| 55810 (positive) 56603 | triplets2 |
| 55810 (positive) 8754 | triplets2 |
| 3659 (negative) 9321 | triplets2 |
| 3659 (positive) 53344 | triplets2 |
| 3659 (positive) 220965 | triplets2 |
| 3659 (positive) 668 | triplets2 |
| 3659 (positive) 22941 | triplets2 |
| hsa-mir-106a-5p (negative) 3659 | triplets2 |
| hsa-mir-106a-5p (negative) 3659 | triplets2 |
| hsa-mir-106a-5p (negative) 3659 | triplets2 |
| hsa-mir-106a-5p (negative) 3659 | triplets2 |
| hsa-mir-106a-5p (negative) 3659 | triplets2 |
| hsa-mir-106a-5p (negative) 3659 | triplets2 |
| hsa-mir-106a-5p (negative) 3659 | triplets2 |
| hsa-mir-17-5p (negative) 3659 | triplets2 |
| hsa-mir-17-5p (negative) 3659 | triplets2 |
| hsa-mir-17-5p (negative) 3659 | triplets2 |
| hsa-mir-17-5p (negative) 3659 | triplets2 |
| hsa-mir-17-5p (negative) 3659 | triplets2 |
| hsa-mir-17-5p (negative) 3659 | triplets2 |
| hsa-mir-106a-5p (negative) 3659 | triplets2 |
| hsa-mir-106a-5p (negative) 3659 | triplets2 |
| hsa-mir-17-5p (negative) 3659 | triplets2 |
| hsa-mir-106a-5p (negative) 3659 | triplets2 |
| hsa-mir-17-5p (negative) 3659 | triplets2 |
| hsa-mir-20a-5p (negative) 55810 | triplets2 |
| hsa-mir-17-5p (negative) 55810 | triplets2 |
| hsa-mir-17-5p (negative) 55810 | triplets2 |
| hsa-mir-20a-5p (negative) 3659 | triplets2 |
| hsa-mir-106a-5p (negative) 55810 | triplets2 |
| hsa-mir-20a-5p (negative) 3659 | triplets2 |
| hsa-mir-106a-5p (negative) 3659 | triplets2 |
| hsa-mir-17-5p (negative) 55810 | triplets2 |
| hsa-mir-106a-5p (negative) 55810 | triplets2 |
| hsa-mir-20b-5p (negative) 55810 | triplets2 |
| hsa-mir-20b-5p (negative) 55810 | triplets2 |
| hsa-mir-17-5p (negative) 3659 | triplets2 |
| hsa-mir-106a-5p (negative) 3659 | triplets2 |
| hsa-mir-17-5p (negative) 3659 | triplets2 |
| hsa-mir-20a-5p (negative) 3659 | triplets2 |
| hsa-mir-106a-5p (negative) 55810 | triplets2 |
| hsa-mir-106a-5p (negative) 55810 | triplets2 |
| hsa-mir-20a-5p (negative) 55810 | triplets2 |
| hsa-mir-20b-5p (negative) 55810 | triplets2 |
| hsa-mir-20b-5p (negative) 55810 | triplets2 |
| hsa-mir-20a-5p (negative) 55810 | triplets2 |
| hsa-mir-20a-5p (negative) 3659 | triplets2 |
| hsa-mir-20a-5p (negative) 3659 | triplets2 |
| hsa-mir-20a-5p (negative) 3659 | triplets2 |
| hsa-mir-20a-5p (negative) 3659 | triplets2 |
| hsa-mir-20a-5p (negative) 3659 | triplets2 |
| 10746 (negative) hsa-mir-106a-5p | triplets2 |
| 26278 (positive) hsa-mir-106a-5p | triplets2 |
| 9321 (positive) hsa-mir-106a-5p | triplets2 |
| 27042 (positive) hsa-mir-106a-5p | triplets2 |
| 53344 (negative) hsa-mir-106a-5p | triplets2 |
| 23143 (negative) hsa-mir-106a-5p | triplets2 |
| 220965 (negative) hsa-mir-106a-5p | triplets2 |
| 10746 (negative) hsa-mir-17-5p | triplets2 |
| 26278 (positive) hsa-mir-17-5p | triplets2 |
| 9321 (positive) hsa-mir-17-5p | triplets2 |
| 27042 (positive) hsa-mir-17-5p | triplets2 |
| 53344 (negative) hsa-mir-17-5p | triplets2 |
| 200424 (negative) hsa-mir-17-5p | triplets2 |
| 169792 (negative) hsa-mir-106a-5p | triplets2 |
| 668 (negative) hsa-mir-106a-5p | triplets2 |
| 668 (negative) hsa-mir-17-5p | triplets2 |
| 57496 (positive) hsa-mir-106a-5p | triplets2 |
| 57496 (positive) hsa-mir-17-5p | triplets2 |
| 10622 (positive) hsa-mir-20a-5p | triplets2 |
| 10622 (positive) hsa-mir-17-5p | triplets2 |
| 8754 (negative) hsa-mir-17-5p | triplets2 |
| 26278 (positive) hsa-mir-20a-5p | triplets2 |
| 8754 (negative) hsa-mir-106a-5p | triplets2 |
| 27042 (positive) hsa-mir-20a-5p | triplets2 |
| 53944 (positive) hsa-mir-106a-5p | triplets2 |
| 56603 (negative) hsa-mir-17-5p | triplets2 |
| 10622 (positive) hsa-mir-106a-5p | triplets2 |
| 10611 (positive) hsa-mir-20b-5p | triplets2 |
| 10622 (positive) hsa-mir-20b-5p | triplets2 |
| 53944 (positive) hsa-mir-17-5p | triplets2 |
| 22941 (negative) hsa-mir-106a-5p | triplets2 |
| 22941 (negative) hsa-mir-17-5p | triplets2 |
| 53944 (positive) hsa-mir-20a-5p | triplets2 |
| 56603 (negative) hsa-mir-106a-5p | triplets2 |
| 10611 (positive) hsa-mir-106a-5p | triplets2 |
| 56603 (negative) hsa-mir-20a-5p | triplets2 |
| 8754 (negative) hsa-mir-20b-5p | triplets2 |
| 56603 (negative) hsa-mir-20b-5p | triplets2 |
| 8754 (negative) hsa-mir-20a-5p | triplets2 |
| 9321 (positive) hsa-mir-20a-5p | triplets2 |
| 53344 (negative) hsa-mir-20a-5p | triplets2 |
| 220965 (negative) hsa-mir-20a-5p | triplets2 |
| 668 (negative) hsa-mir-20a-5p | triplets2 |
| 22941 (negative) hsa-mir-20a-5p | triplets2 |

**Figure S3. PMF network.** The network gives a non redundant and comprehensive picture of most modulated paths in the two PMF vs CTR comparisons, of the impact of miRNAs on pathway genes, and of connected TF-miRNA-gene mixed circuits discovered in the study. Genes are reported as round rectangles, transcriptional factors as diamonds and miRNAs as triangles. Node colours represent the fold-change (FC) of the gene expressions in the PMF *vs* BM (node inner colour) and PMF *vs*
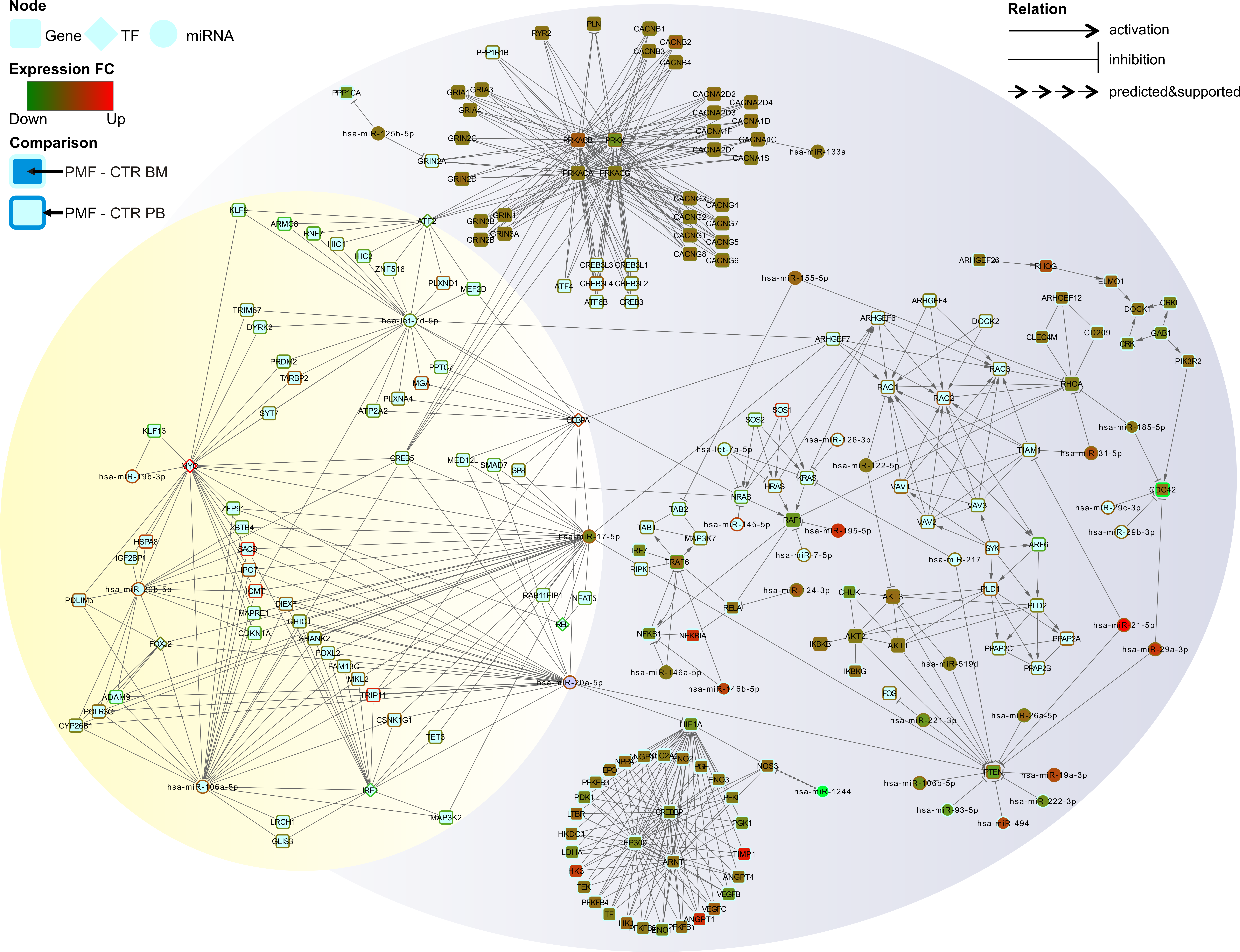
 PB (node border colour). The type of edges depends on the type of interaction: arrow for activation, T arrow in case of inhibition, arrow line for miRNA-target predicted/supported interactions.

**7) Cluster analysis of expression profiles of miRNAs and genes included in the final PMF network do not show clustering of PMF patients by mutation.**

**Figure S4.** Heatmap obtained by PMF samples clustering according to Euclidean distance and complete clustering applied to miRNAs and genes included in the final PMF network. Samples are colored according to the carried mutation as shown in the legend (3N indicates triple negative).


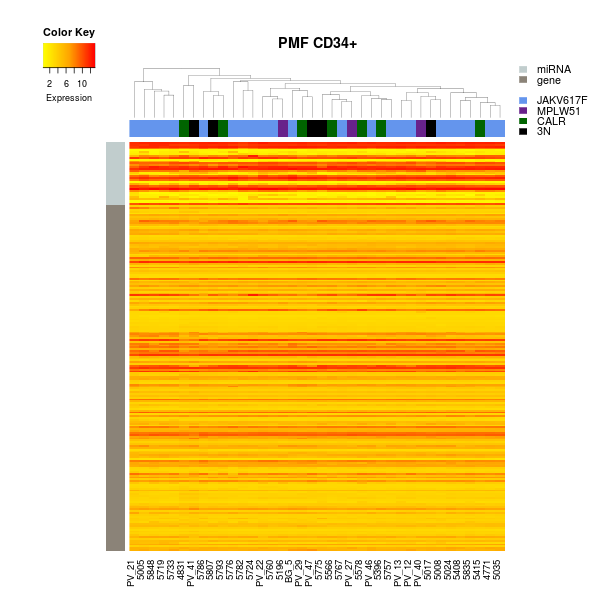


**Figure S5. Comparison fold-changes of miRNA and gene expression included in the PMF network in three subgroups of patients homogeneous for the carried mutation and in triple negatives.** As shown in the legend, the fold-changes observed in PMF vs CTR BM and PMF vs CTR PB comparisons are respectively shown by the colors of the center and of the border of nodes.


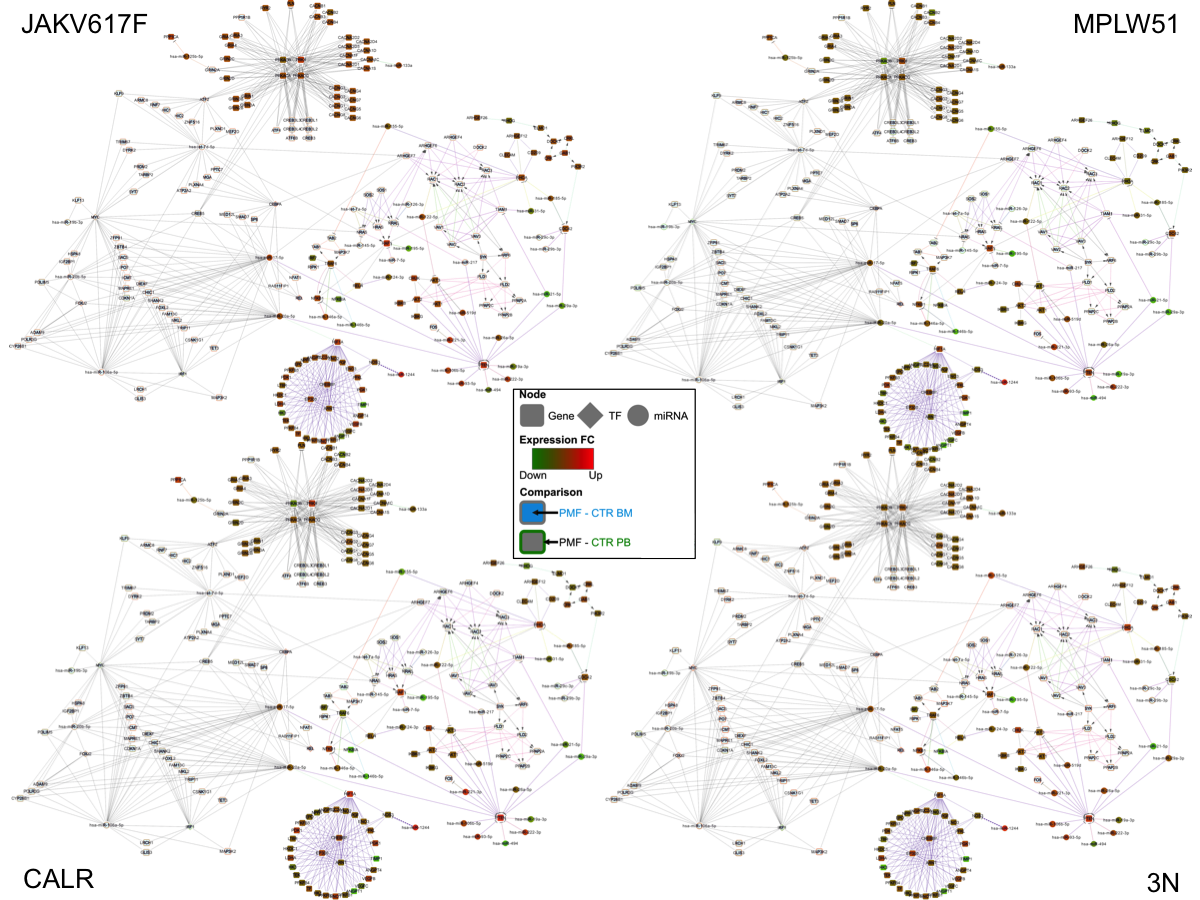

Supplement: Supplementary Information [file bcj201647x1.doc]
